# Introduction of flavin anions into photoredox catalysis: acid–base equilibria of lumichrome allow photoreductions with an anion of an elusive 10-unsubstituted isoalloxazine†

**DOI:** 10.1039/d5sc01630d

**Published:** 2025-05-15

**Authors:** Dorota Prukała, Ekaterina Zubova, Eva Svobodová, Ludmila Šimková, Naisargi Varma, Josef Chudoba, Jiří Ludvík, Gotard Burdzinski, Iwona Gulaczyk, Marek Sikorski, Radek Cibulka

**Affiliations:** a Faculty of Chemistry, Adam Mickiewicz University 61-614 Poznań Poland sikorski@amu.edu.pl dorota.prukala@amu.edu.pl; b Department of Organic Chemistry, University of Chemistry and Technology, Prague 16628 Prague Czech Republic cibulkar@vscht.cz; c Department of Molecular Electrochemistry and Catalysis, J. Heyrovský Institute of Physical Chemistry, Czech Academy of Sciences 18223 Prague Czech Republic; d Central Laboratories, University of Chemistry and Technology, Prague 16628 Prague Czech Republic; e Faculty of Physics and Astronomy, Adam Mickiewicz University 61-614 Poznań Poland

## Abstract

Flavins have been established as effective catalysts in oxidative photoredox catalysis. Conversely, their use in reductive photocatalysis remains limited, mainly due to the relatively low stability of the transient flavin radicals (semiquinones), which are used in photoreductions. The fully reduced forms of flavins are also disadvantaged in photocatalysis because they absorb light in the UV rather than in the visible region. In this work, we present a new approach for reductive flavin photocatalysis that utilises a flavin (isoalloxazine) anion derived from the elusive 10-unsubstituted 3,7,8-trimethylisoalloxazine, an unstable tautomer of 3-methyllumichrome. We found the conditions under which this isoalloxazine anion is formed by *in situ* deprotonation/isomerisation from the readily available 3-methyllumichrome and we subsequently used it as a photoredox catalyst in the reductive dehalogenation of activated bromoarenes and their C–P coupling reaction with trimethyl phosphite to form an arylphosphonate. Steady-state and transient absorption spectroscopy, NMR and cyclic voltammetry investigations, together with quantum chemical calculations, showed that the anion of oxidised isoalloxazine has several advantages, compared to other forms of flavins used in photoreductions, such as high stability, even in the presence of oxygen, an absorption maximum in the visible region, thereby allowing the use of excitation light between 470 and 505 nm, and a relatively long-lived singlet excited-state.

## Abstract 2

Deprotonation of 3-methyllumichrome results in an anion of elusive 10-*H*-isoalloxazine which acts as a strong reducing agent upon blue/cyan light excitation.

## Graphical abstract

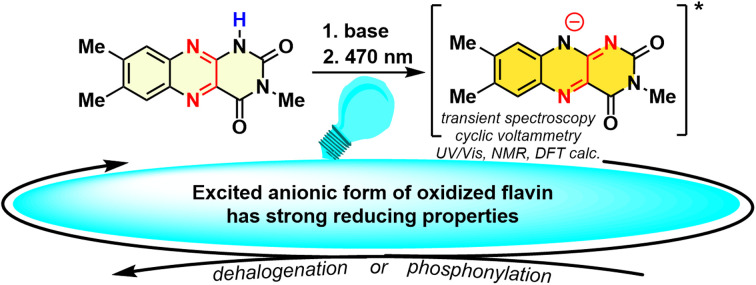

## Introduction

**Fig. 1:**
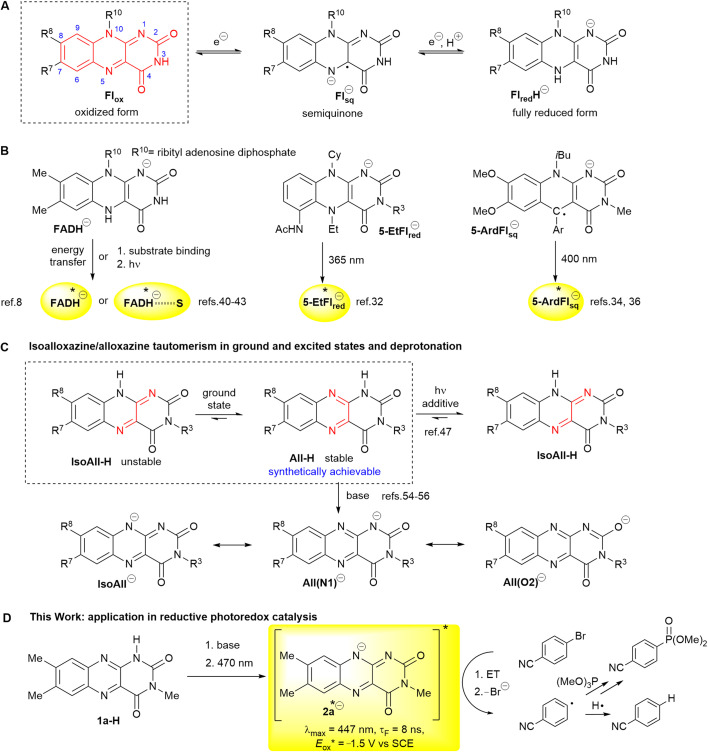
(A) Redox states of flavins with an isoalloxazine ring (in red) and its numbering (acid–base equilibria are omitted); (B) anions of flavins and their derivatives used in photochemical transformations either in enzyme reactions or in artificial systems; (C) isoalloxazine (IsoAll-H)/alloxazine (All-H) tautomerism and important resonance structures of the corresponding anion. For clarity, the structures are abbreviated according to the parent protonated structures; (D) application of the flavin-10-ide anion 2a^−^‡ in photoredox catalysis developed in this work.

Flavins are redox-active cofactors that are characterised by an isoalloxazine unit.^1–3^ Flavins exist in three redox states—fully oxidised, semiquinone (radical) and fully reduced forms—with several possibilities for protonation/deprotonation (Fig. 1A). Most of these states are photoactive; thus, flavins can participate in many photochemical events, such as bioluminescence, phototropism and the regulation of biological clocks.^4–7^ A few flavin-dependent photoenzymes are known to catalyse chemical transformations in nature^8,9^ or have been tested in photobiocatalysis studies.^10–17^

Flavins have also been established as efficient catalysts in photoredox catalysis, especially when carrying out oxidative transformations.^18–31^ This catalytic capacity arises particularly due to the stability of the oxidised form and its ability to become strongly oxidising upon excitation with visible light at around 450 nm. Conversely, the reduced forms of flavins are destined for reduction. Nevertheless, the use of flavins in photoreductions remains rather rare,^11,32–36^ largely because of the instability of the reduced forms, their short-lived excited states outside an enzyme,^37,38^ and the weak absorption of their fully reduced forms in the visible region.^39^ Only a few approaches have endeavoured to overcome these disadvantages (Fig. 1B). In photolyases, enzymes providing nucleic acid repair, the reduced deprotonated flavin adenine dinucleotide (FADH^−^) is excited through energy transfer from an antenna system.^8^ Zhao and Hyster utilised the coloured complexes of the reduced flavin cofactor with a substrate formed in the protein active site of an enzyme.^40–43^ Storch took advantage of steric hindrance by introducing substitutions at the 5- and 6-positions to prepare a stable reduced flavin analogue 5EtFl_red_^−^.^32^ In our group, anionic semiquinones of 5-deazaflavins 5ArdFl_sq_^−^ were stabilised by aryl substitution at the 5-position.^34,36^

We hypothesised that the use of an anion of the oxidised form might be a novel approach for incorporating flavins into photoreductive chemistry. The use of excited anions in reductions is advantageous since the negative charge facilitates oxidation and thus increases the reducing power of the catalyst.^44–46^ Moreover, the red shift of the absorption spectrum caused by deprotonation allows the utilisation of visible light instead of highly energetic UV light. We considered that a flavin-10-ide IsoAll^−^, which is a deprotonation product of the elusive N(10)-H isoalloxazine IsoAll-H, could serve as a suitable candidate for reductive visible light photoredox catalysis. IsoAll-H, on the one hand, is inaccessible because of its tautomerisation to N(1)–H alloxazine All-H (Fig. 1C), which is more stable by 54.9 kJ mol^−1^ (ref. 47). On the other hand, flavin-10-ide IsoAll^−^ might be generated from its stable alloxazine tautomer All-H, which was reported to undergo phototautomerisation and/or deprotonation under various conditions.^48–53^ Recently, most authors have proposed that, in a strongly basic environment (*e.g.* in water at pH > 14), the desired anion is formed even in the ground state.^54–56^ Note that IsoAll^−^ represents only one of the possible resonance forms (see Fig. 1C).‡We use designation 2a^−^ for anion formed from 1a-H keeping in mind existence of other resonance forms.

In this study, we present a detailed investigation of the behaviour of 3-methyllumichrome 1a-H and its derivatives under basic conditions with the aim of elucidating suitable conditions for its transformation to the desired anion 2a^−^ under conditions suitable for photoreductions (Fig. 1D). This investigation was conducted using a combination of various techniques, including UV-vis and fluorescence spectroscopy, NMR, electrochemistry and quantum chemical (DFT) calculations. This strategy allowed us to recognise the prevailing isoalloxazinic character of the anionic species and the suitability of its photophysical and electrochemical properties for photoredox catalysis. Finally, we introduced 2a^−^ into an unprecedented photoreductive system and confirmed its usefulness in reductive dehalogenation and a C–P coupling reaction.

## Results and discussion

**Fig. 2:**
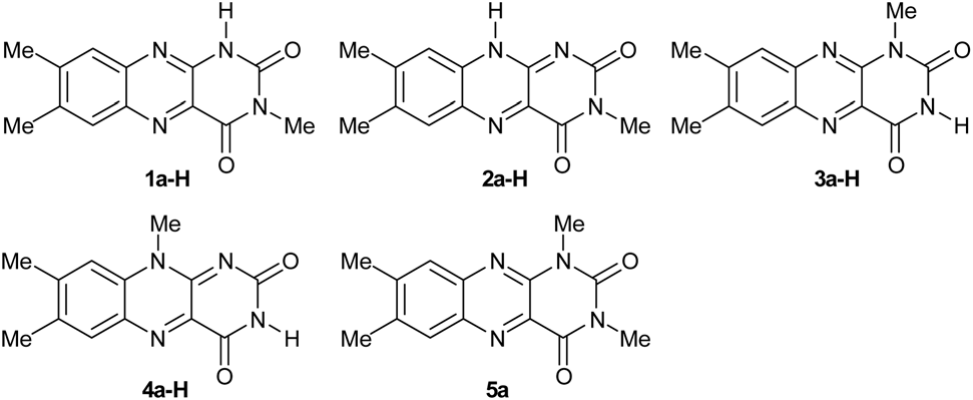
Isoalloxazines and alloxazines involved in the study. 2a-H represents a hypothetical structure, which was investigated using DFT calculations.

To clarify the alloxazine → isoalloxazine tautomerisation/de-protonation process for its subsequent use in photoredox catalysis, we selected 3-methyllumichrome (1a-H), which has a single acidic hydrogen atom N(1)–H that allows tautomerisation to 2a-H. For comparison, we also included alloxazine (3a-H) and isoalloxazine (4a-H), which contain acidic N(3)–H, and derivative 5a, which lacks acidic hydrogen (Fig. 2). When possible, the experiments were performed using acetonitrile as a suitable organic solvent. Alternatively, other dipolar aprotic solvents, such as *N*,*N*-dimethylformamide (DMF), dimethyl sulfoxide (DMSO) or their mixtures, were used if problems with solubility arose. We used tetrabutylammonium acetate (TBAOAc) as a non-nucleophilic base because it is a relatively strong base in acetonitrile [p*K*_a_ (acetic acid) = 22.2]^57^ and has good solubility in organic solvents.

**Fig. 3:**
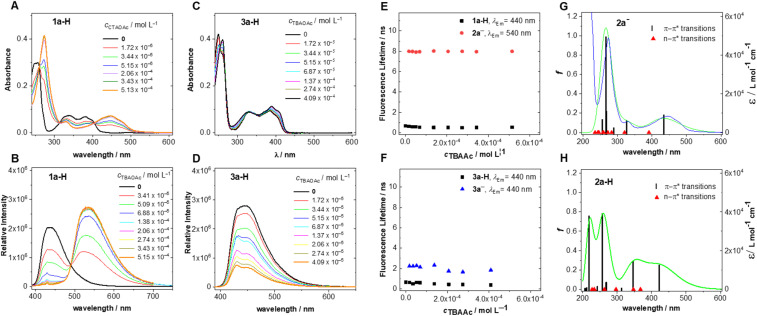
(A) Experimental UV-vis and (B) experimental fluorescence spectra of 1a-H in acetonitrile (*c* = 8.49 × 10^−6^ mol L^−1^, *λ*_ex_ = 380 nm) in the absence and presence of various amounts of TBAOAc; (C) experimental UV-vis spectra and (D) experimental fluorescence spectra of 3a-H in acetonitrile (*c* = 1.24 × 10^−5^ mol L^−1^, *λ*_ex_ = 380 nm) in the absence and presence of various amounts of TBAOAc; (E and F) plots of the fluorescence lifetimes of different forms of 1a-H and 3a-H, respectively, as a function of TBAOAc concentration and emission wavelength; *λ*_ex_ = 368 nm; (G and H) experimental absorption spectrum of anion formed from 1a-H in acetonitrile (blue line) and the corresponding results of calculations for 2a^−^ and the structure 2a-H; oscillator strengths, *f*, calculated as the lowest predicted S_0_ → S_i_ excitation energies (B3LYP/aug-cc-pVTZ), with acetonitrile included as a solvent in the model, and theoretical absorption spectra (green line).

As expected, the addition of TBAOAc to an acetonitrile solution of 1a-H caused significant changes in the UV-vis spectra (Fig. 3A) showing a clear isosbestic point. Characteristic alloxazinic-type absorption bands with maxima at 335 nm and 380 nm were replaced by a new band at 447 nm, which is typical of isoalloxazine structures. A significant change was observed in the fluorescence spectra. Upon excitation with 380 nm light, alloxazine 1a-H showed a fluorescence signal with the maximum at 437 nm, but this changed to one with a maximum at 534 nm in the presence of an excess of TBAOAc (Fig. 3B). In contrast, only a relatively small change in the UV-vis spectra and almost no change in the fluorescence spectra maximum were observed for 3a-H upon the addition of TBAOAc (Fig. 3C and D). No changes were observed in the absorption and emission spectra (see Fig. S4.1 and S4.2†) for the less acidic isoalloxazine 4a-H [for illustration, p*K*_a_ (3a-H) = 8.5,^58^ p*K*_a_ (4a-H) = 10.2 (ref. 59) in water] and, as expected, for 5a that lacked an acidic hydrogen.

These observations can be explained by a major change in the 1a-H structure upon deprotonation and a resulting formation of an anion stabilised by a resonance (see Fig. 1C), with a significant contribution of isoalloxazinic structure 2a^−^ (=IsoAll^−^, R^7^=R^8^=CH_3_). Conversely, deprotonation of 3a-H produced anion 3a^−^ with no conjugation to benzene and the central rings of alloxazine. Very importantly, this explanation was confirmed by theoretical calculations performed at the B3LYP/aug-cc-pVTZ level with the solvent (acetonitrile) involved. The spectra of 1a-H upon the addition of a base corresponded well to the theoretical spectrum of the anion (Fig. 3G) rather than to that of the hypothetical isoalloxazine tautomer 2a-H (Fig. 3H). This clearly demonstrates that not only tautomerisation but also deprotonation occurs in the ground state upon addition of a base.

The conclusions raised from the experimental absorption/emission spectra and theoretical calculations were also in agreement with the excitation spectra (see Fig. S4.3†) and, importantly, with the fluorescence lifetimes measured upon excitation with 368 nm light. The fluorescence kinetics of 1a-H in acetonitrile with different amounts of TBAOAc, recorded at the emission at 440 nm and 540 nm, gave monoexponential decays for both observed wavelengths (Fig. 3E). The shorter-lived component observed at 440 nm with a fluorescence lifetime *τ*_F_ = 0.66 ns corresponds to the neutral alloxazinic form of 1a-H (the literature value^60^ is *τ*_F_ = 0.64 ns). The longer-lived component observed at 540 nm, with a lifetime of about 8.0 ns, corresponds to the stabilised isoalloxazinic anion of 2a^−^.‡ Its lifetime is significantly longer than that of the alloxazinic anion formed from 3a-H (*τ*_F_ = 2.2 ns, Fig. 3F). Note that the fluorescence lifetimes for non-dissociated isoalloxazine 4a-H and alloxazine 5a in acetonitrile are 7.7 ns and 0.64 ns, respectively.^60^

**Fig. 4:**
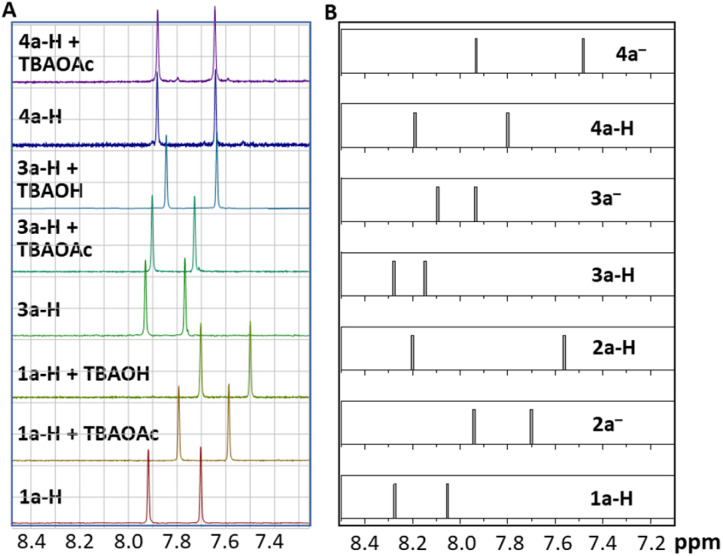
(A) Experimental chemical shifts of the aromatic protons in the ^1^H NMR spectra of selected flavins in CD_3_CN-DMSO-d_6_ (85 : 15, v/v) in the absence and presence of an excess of base (10 equiv.); (B) chemical shifts of selected neutral and deprotonated flavin derivatives obtained by DFT calculations (GIAO/B3LYP/aug-cc-pVTZ); for full theoretical spectra, see Fig. S7.7–S7.9 and Tables S7.5–S7.7.†

The behaviour of the model compounds was also investigated using ^1^H NMR spectroscopy and the results obtained in acetonitrile-d_3_-dimethyl sulfoxide-d_6_ mixture (85 : 15, *v*/*v*, used because of solubility) agreed with the absorption/emission spectra measurements. A significant change in the chemical shifts of the signals of aromatic protons H(6) and H(9) was observed upon the addition of TBAOAc to 1a-H, although even an addition of 10 equivalents was not sufficient for complete deprotonation (Fig. 4A and S7.1–S7.4†). Quantitative deprotonation was achieved by using a stronger base, such as sodium hydride (not shown in Fig. 4) or tetrabutylammonium hydroxide (TBAOH). Only a small but remarkable shift of aromatic signals was observed upon base addition to the solution of 3a-H (Fig. 4A and Table S7.3†).

Importantly, the described processes are reversible, as can be deduced from the proton signals that returned upon the addition of trifluoroacetic acid (Tables S7.3, S7.4 and Fig. S7.5, S7.6†). The less acidic 4a-H and 5a without acidic hydrogen showed no change in the shift of the signal in ^1^H NMR spectra with TBAOAc (see Table S7.3†). An attempt to deprotonate 4a-H with a stronger base, such as sodium hydride or TBAOH, resulted in flavin decomposition.

The trends observed in the ^1^H NMR experimental data were also in accord with the chemical shifts obtained using DFT calculations (Fig. 4B). Deprotonation of 1a-H was proposed to be accompanied by a significant shift of both the H(6) and H(9) protons by approximately 0.3 ppm to lower ppm values. Conversely, the hypothetical tautomerisation to 2a-H (without deprotonation) would be reflected by a large shift of 0.5 ppm for H(9), but only a small shift (<0.1 ppm) in the H(6) signal. Both the experimental and theoretical shifts of H(6) and H(9) caused by deprotonation of 3a-H were about 0.15 ppm (*i.e.* smaller than for 1a-H deprotonation), as in the experimental results. Note that the absolute values of the theoretical ^1^H NMR shifts are shifted compared to the experimental ones.

**Fig. 5:**
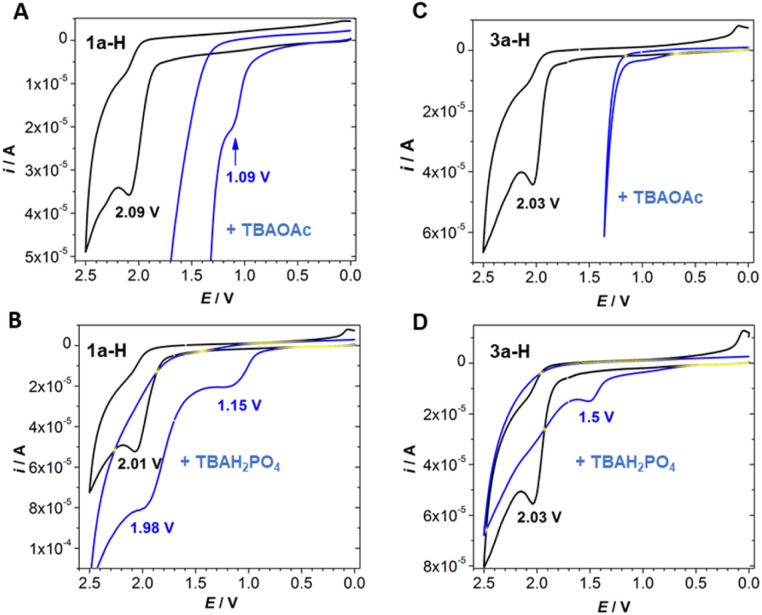
Cyclic voltammograms of 1a-H (A and B) and 3a-H (C and D) in the absence and presence of 50 equiv. of TBAOAc (data in A and C) or TBAH_2_PO_4_ (data in B and D). Data were obtained in acetonitrile in the presence of TBAPF_6_ (*c* = 0.1 mol L^−1^) using a glassy carbon working electrode, a saturated calomel reference electrode (SCE) and a platinum auxiliary electrode (see ESI S5† for details).

In general, the oxidation potentials of organic molecules are significantly influenced by deprotonation, and this is even more pronounced if the anion is stabilised by resonance. Typical examples are phenols, which have oxidation potentials of +1.19 V *vs.* SCE for neutral forms and −0.10 V *vs.* SCE for deprotonated forms [values for 2,4,6-tri(*tert*-butyl)phenol].^44^ A similar significant change in the oxidation potential was observed by cyclic voltammetry for 1a-H (Fig. 5A). We found an irreversible anodic wave with *E*_p_ = +2.09 V *vs.* SCE in an acetonitrile solution of 1a-H using a glassy carbon working electrode and a TBAPF_6_ supporting electrolyte. After TBAOAc addition, a new oxidation wave with *E*_p_ = +1.09 V *vs.* SCE appeared, which can be explained by the formation of a stabilised anion. Conversely, 3a-H, 4a-H and 5a, which are characterised by anodic waves with *E*_p_ = +2.03 V, +1.87 V and +2.06 V *vs.* SCE, respectively, showed no anodic wave after the addition of TBAOAc within the electrochemical window, which was narrowed to +1.3 V because of acetate oxidation (see Fig. 5C and S5.1†). Therefore, we performed experiments using a tetrabutylammonium dihydrogen phosphate (TBAH_2_PO_4_) base, which is not subject to oxidation. As expected, we observed a significantly less positive oxidation potential for the more stabilised anion of 1a-H (+1.15 V *vs.* SCE) than that of 3a-H (+1.50 V *vs.* SCE) (Fig. 5B and D).

**Fig. 6:**
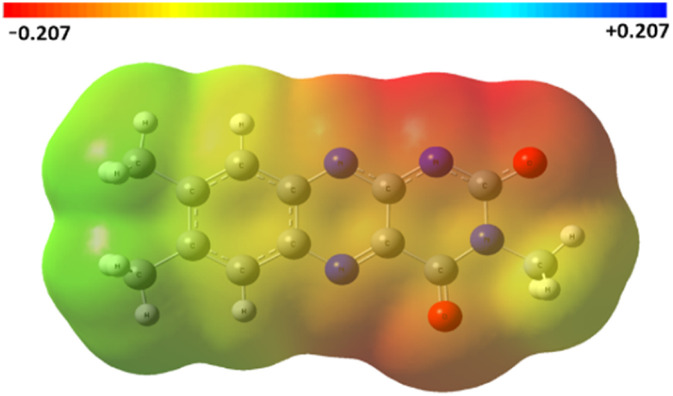
Electrostatic surface potential (ESP) for the anion formed from 1a-H, as calculated from the optimised geometries using the DFT-B3LYP method and the basis function aug-cc-pVTZ (for natural population analysis data, see Fig. S10.1 and Table S10.1;† for data related to the other anions derived from 3a-H and 4a-H, see Fig. S10.2, S10.3 and Tables S10.2, S10.3†).

A question arose regarding the structure of the anion formed by deprotonation of 1a-H. Based on DFT calculations focused on relative electron density, we can propose an almost equal contribution of resonance forms (see Fig. 6 for an illustration and ESI S10† for more details). This means that the contribution of the flavin-10-ide anion 2a^−^ to the resonance hybrid is significant. Thus, keeping in mind its absorption spectrum (Fig. 2A), we proposed its application in photoredox catalysis, even with light wavelengths above 450 nm.

To illustrate the suitability of 2a^−^‡ in reductive photoredox catalysis, we investigated its use for the dehalogenation of aryl halides, which is considered a benchmark reaction because of the negative redox potentials of these compounds. Considering the thermodynamic parameters (*i.e.* the reduction potential of aryl halide and excited state oxidation potential of 2a^−^ [
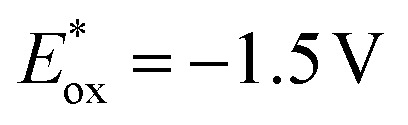
*vs.* SCE,§§
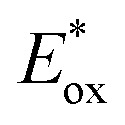
 of 2a^−^ in single excited state was estimated from ground state redox potential (*E*_p/2_ = 1.02 V; *i.e.* value from CV measurement of 1a-H in the presence of TBAOAc) and the value *E*^0–0^ = 2.52 eV according to equation 
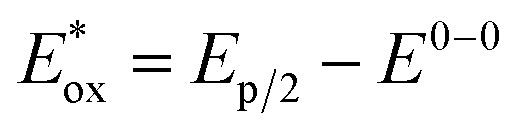
 (ref. 71). see also ESI S6†]), we suggested 4-bromobenzonitrile (*E*_red_ = −1.59 V *vs.* SCE)^61^ as a substrate for this purpose. The experiments were performed in the presence of a catalytic amount of 1a-H, a source of 2a^−^, Cs_2_CO_3_ as the base and γ-terpinene as the hydrogen atom source. We used DMF as a solvent instead of acetonitrile to maintain the catalyst dissolved at the concentrations needed for catalysis. To our delight, we observed an almost quantitative yield of the dehalogenated product, benzonitrile (7a), following 16 hours of LED irradiation at 470 nm (Table 1, entry 1). This result demonstrates the catalytic function of flavin anion 2a^−^, keeping in mind that the parent neutral alloxazine 1a-H does not absorb in this region. In contrast, the reaction was significantly less effective in the presence of the other derivatives 4a-H and 5a and was not effective with 3a-H (entries 2–4). A loss of effectiveness was also observed when using other bases, such as CsOAc and TBAH_2_PO_4_ (entries 5 and 6), longer wavelength light (entry 7) or in the absence of an additional hydrogen atom source (entry 8) – in the latter case DMF likely acts as a hydrogen atom donor.^62^

**Table 1:** Photoreductive dehalogenation catalysed by anion formed from 1a-H and the corresponding control experiments

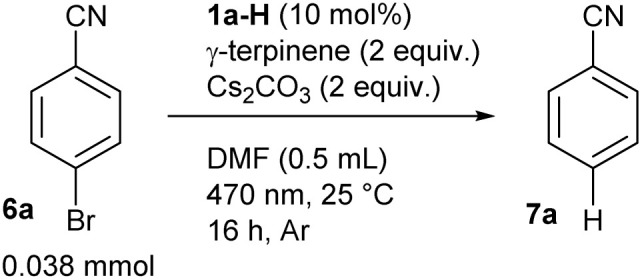		
Entry	Conditions alternation	Yield of dehalogenation product^a^ [%]
1	—	84
2	3a-H instead of 1a-H	5
3	4a-H instead of 1a-H	35
4	5a instead of 1a-H	30^b^
5	CsOAc instead of Cs_2_CO_3_	14
6	TBAH_2_PO_4_ instead of Cs_2_CO_3_	45
7	505 instead of 470 nm	34^b^
8	No γ-terpinene	28
9	No Cs_2_CO_3_	9
10	No flavin catalyst	0
11	No light	0
12	No light, 40 °C	0
13	Under air instead of Ar	82
14	1-Bromo-4-(trifluoromethyl)benzene (6b)	21
15	4-Bromoanisole (6c)	5
16	3-Bromofluorenone (6d)/505 nm	75

a Determined by GC-MS. Conditions: *c* (substrate) = 0.076 mol L^−1^; *c* (1a-H) = 7.6 × 10^−3^ mol L^−1^; *c* (γ-terpinene) = 0.152 mol L^−1^; *c* (base) = 0.152 mol L^−1^; for details see ESI S3 and S8.

b Average value.

Regarding the choice of base, CsOAc and TBAH_2_PO_4_ are weaker bases than Cs_2_CO_3_ and a larger excess would be necessary for quantitative conversion of 1a-H to its anionic form. Nevertheless, using 2 equivalents of TBAH_2_PO_4_ still resulted in the formation of a significant amount of product 7a (entry 6). Control experiments confirmed that a base, flavin catalyst and light are needed (entries 9–11). The reaction also did not proceed in the dark, even at elevated temperature (entries 11 and 12). Interestingly, conducting the reaction in air did not slow down the reaction (entry 13), which is unusual for reductive photoredox catalysis but is useful from a practical point of view. As expected, the less electron-deficient 1-bromo-4-(trifluoromethyl)benzene (6b; *E*_red_ = −2.18 V *vs.* SCE)^63^ and the electron-rich 4-bromoanisole (6c; *E*_red_ = −2.78 V *vs.* SCE)^64^ were beyond the limit of 2a^−^, keeping in mind its reductive power provided by the excited state oxidation potential (entries 14 and 15). On the other hand, dehalogenation products were obtained in high yield with 3-bromofluorenone (6d; *E*_red_ = −1.19 V *vs.* SCE)^65^ which is easier to reduce in terms of reduction potential (entry 16).

**Scheme 1:**
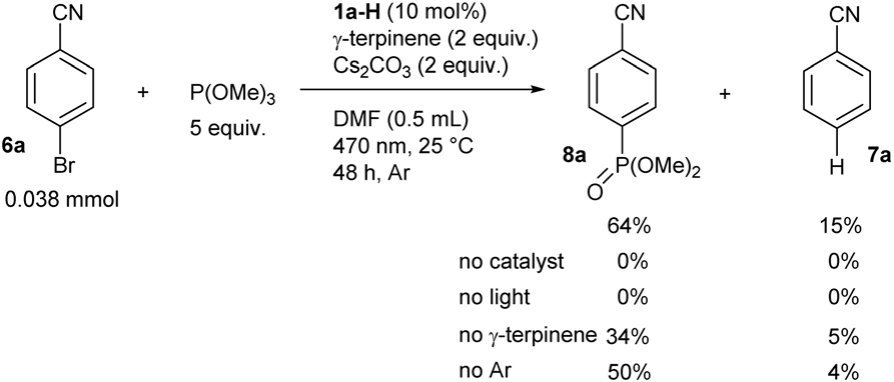
The reductive C–P coupling reaction provided by photoredox catalysis based on the anion formed from 1a-H (yields determined by GC-MS, see ESI S3 and S8† for details).

We also demonstrated the usefulness of our strategy in coupling reactions by performing a reaction using bromobenzonitrile (6a) and trimethyl phosphite under the developed conditions (Scheme 1). In addition to a minor production of dehalogenation product 7a, we observed a good yield of phenylphosphonate 8a resulting from the C–P coupling reaction. The reaction did not occur in the absence of the catalyst or light and showed a reduced yield in the absence of γ-terpinene, which is beneficial for catalyst regeneration (see below). Interestingly, coupling reaction occurred also under air with only slightly reduced yield.

**Fig. 7:**
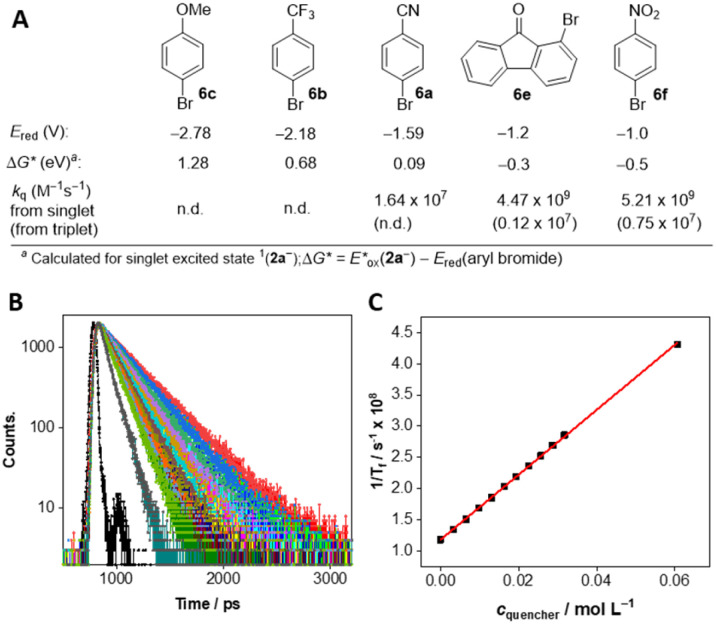
(A) Quenching rate constants (in units of M^−1^ s^−1^) for 2a^−^ in the singlet or triplet excited state by bromoarenes and the corresponding thermodynamic data for electron transfer from 2a^−^ in the singlet excited state. For references on reduction potentials, see text. Fluorescence lifetime quenching of 2a^−^ with 6f; (B) fluorescence decays of 2a^−^ at different concentration of 6f and (C) the corresponding Stern–Volmer plot.

We conducted time-resolved spectroscopy experiments to confirm the idea that excited anion 2a^−^* is involved in the dehalogenation reaction of 4-bromobenzonitrile (6a). As mentioned above, the anionic species 2a^−^, which is formed from 1a-H, is characterised by relatively long-lived fluorescence at 537 nm. The fluorescence lifetime measured at 540 nm (upon excitation at 449 nm) was slightly shortened by the addition of 6a, as evident from the Stern–Volmer plot (see Fig. S4.4†). The rate constant of electron transfer estimated from these measurements (*k*_q_ = 1.64 × 10^7^ M^−1^ s^−1^) is relatively low, but it corresponds to a Δ*G*_ET_ value around 0 obtained from redox potentials (see Fig. 7A). As expected, the fluorescence quenching was much more pronounced when using substrates with less negative reduction potentials, such as 1-bromofluorenone (6e) or 1-bromo-4-nitrobenzene (6f), with *k*_q_ values about 5 × 10^9^ M^−1^ s^−1^ demonstrating very effective photoinduced electron transfer (Fig. 7B, C and S4.5, S4.6†). Quenching of 2a^−^ in its triplet excited state was significantly slower (see rate constants in Fig. 7A and data in Fig. S4.7 and S4.8†) but its contribution cannot be avoided because of significantly longer triplet lifetime (57 μs compared to 8 ns for singlet excited state). On the other hand, we have some indications that singlet excited state of 2a^−^ plays major role in reductive dehalogenation: (i) triplet lifetime is significantly shortened under air (to 1.8 μs; see Fig. S4.9†) while catalytic performance of 2a^−^ is almost not affected compared to experiments under argon (*cf.* entries 1 and 13 in Table 1), (ii) quantum yield of intersystem crossing is relatively low (value of *Φ*_ISC_ was estimated as 0.08, see ESI S4†).

**Scheme 2:**
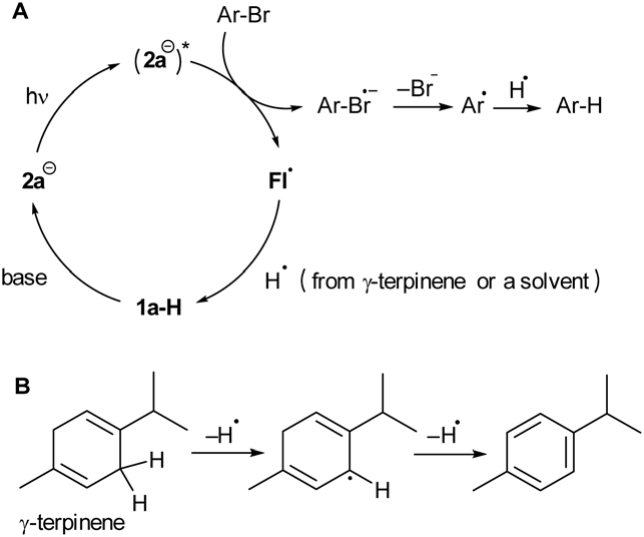
The proposed simplified mechanism of dehalogenation mediated by photoredox catalysis with 2a^−^ (A) involving γ-terpinene as a hydrogen source (B). Solvent cage formation involving radicals upon SET and following escape are omitted. Fl˙ means radical formed by one-electron oxidation of 2a^−^.

Based on the results of the catalytic experiments, as well as the spectral and electrochemical properties of 2a^−^ and the quenching studies of its excited states, we proposed a mechanism for the reductive dehalogenation provided by 1a-H under basic conditions (Scheme 2). 1a-H deprotonates to form 2a^−^, which is the only species that absorbs 470 nm light. Upon excitation, electron transfer occurs from (2a^−^)* to an aryl halide resulting to a solvent-caged radical pair. Upon successful cage escape,^66^ a phenyl radical is formed, which can subsequently take a hydrogen atom from either γ-terpinene or DMF. Alternatively, the phenyl radical reacts with trimethyl phosphite to form a C–P coupling product (not shown in Scheme 2). A flavin radical Fl^•^ generated by electron transfer accepts hydrogen to form 1a-H, which then enters the next catalytic cycle. The fact that the main electron transfer step of the catalytic cycle does not involve an excitation of a transient species, such as a radical anion (as in conPET processes with flavins^32–36^), explains (i) the high efficiency of dehalogenation of substrate 6a, which is at the border of thermodynamic feasibility and (ii) the high efficiency under an air atmosphere.

## Conclusions

We confirmed that an alloxazine like 1a-H, which is not substituted in position 1, undergoes deprotonation that leads to anion 2a^−^.‡ This anion is characterised by isoalloxazinic spectral properties in terms of its absorption and fluorescence spectra and fluorescence lifetime. Due to its anionic character, 2a^−^ becomes strongly reducing upon excitation by blue/cyan light, as demonstrated by reductive dehalogenation of bromobenzenes and C–P coupling reactions. Interestingly, the isoalloxazinic anion 2a^−^, with its negatively charged position N(10), was a more potent catalyst for reductive photocatalysis when compared with the anion 4a^−^ formed by N(3)–H deprotonation of isoalloxazine 4a-H.

This work is the first to demonstrate how to generate and simultaneously utilise the anion of a hypothetical isoalloxazine unsubstituted at position 10 in photoredox catalysis. It is also a demonstration of a new way, how to switch the oxidised flavins, still exclusively used as oxidants, to a stable reductive species. The reducing power of the excited anion 2a^−^, as expressed by its oxidation potential, does not yet reach the values achieved using alkali metals, as is the case with some excited flavin radicals or flavin reduced forms.^32,33,36^ However, the isoalloxazin-10-ide anion 2a^−^ has significant advantages, including its ease of generation, its excitation by visible light in the region of 450 to 505 nm, its high stability, and its efficiency in the presence of oxygen. These features make photocatalytic systems with 2a^−^ strong candidates for practical applications even on a larger scale. Moreover, the flavin structure offers room for modifications that would lead to further increases in the reducing capabilities of the respective anions. From general point of view, the use of an excited closed-shell anion in photoredox catalysis is advantageous compared to the use of excited open-shell organic radical anions which are characterised by short picosecond excited state lifetimes and tendency to (photo)degradation. Thus, we believe that our innovative approach towards the generation of stable flavin anionic species with interesting reducing properties, together with other recent cutting-edge discoveries in flavin catalysis,^67–70^ can initiate novel applications of flavins in synthetic organic chemistry.

## Author contributions

R. C., D. P. and M. S. wrote the paper with input from all of the authors. E. S. and D. P. performed synthesis of all used compounds. D. P. performed the stationary spectroscopy and fluorescence lifetime measurements. N. V. collected quenching experiments. Transient absorption data measurements were done by G. B and N. V. Experimental spectroscopy data were analysed by M. S. E. S. conducted ^1^H NMR studies. E. Z. conducted all photochemical reactions. J. C. did all mass spectrometry analysis. Electrochemical data were measured by L. S. and analysed by L. S. and J. L. Theoretical calculations were performed by I. G. The project was conceived by R. C., D. P. and M. S.

## Conflicts of interest

There are no conflicts to declare.

## Supplementary Material

SC-016-D5SC01630D-s001

## Data Availability

Data for this article, including raw data of cyclic voltammetry, UV-vis, fluorescence, NMR and mass spectra, description of all experiments are available at Zenodo at https://doi.org/10.5281/zenodo.14937054.
