# Supplementary material for: Introduction of flavin anions into photoredox catalysis: acid–base equilibria of lumichrome allow photoreductions with an anion of an elusive 10-unsubstituted isoalloxazine
Source: Chem Sci. 2025 May 15;16(25):11255–63. doi: 10.1039/d5sc01630d (PMC12094105; doi:10.1039/d5sc01630d)
Supplement: SC-016-D5SC01630D-s001 [file SC-016-D5SC01630D-s001.pdf]

Supplementary Information (SI) for Chemical Science.
This journal is © The Royal Society of Chemistry 2025

# Introduction of flavin anions into photoredox catalysis: Acid-base equilibria of lumichrome allow photoreductions with an anion of an elusive 10-unsubstituted isoalloxazine

Dorota Prukała,* Ekaterina Zubova, Eva Svobodová, Ludmila Šimková, Naisargi Varma, Josef Chudoba, Jiří Ludvík, Gotard Burdzinski, Iwona Gulaczyk, Marek Sikorski,* Radek Cibulka*

## Supplementary Information

## Content

S1. Materials and methods ........ 2
S2. Synthesis and characterization of flavin derivatives ........ 3
S3. Photoredox catalysis - experimental ........ 7
S4. UV-Vis, fluorescence and excitation spectra, fluorescence lifetime and UV-Vis transient absorption spectra measurements ........ 9
S5. CV measurements ........ 19
S6. Estimation of $E^*_{ox}$ of **2a**$^-$ in singlet excited state ........ 20
S7. Experimental and theoretical NMR data in the presence of base ........ 21
S8. Photoredox catalysis – analysis of reaction mixtures ........ 34
S9. NMR data for synthesized flavin derivatives ........ 47
S10. Natural population analysis data for anions ........ 52
S11. Cartesian coordinates ........ 55
S12. References ........ 60

# S1. Materials and methods

**Starting materials** were purchased from Merck (Sigma-Aldrich) and Fluorochem. The solvents were purified and dried using standard procedures. Commercially obtained reagents were used as received without further purification unless otherwise stated. The compound structures were drawn and named using ChemDraw. **Nuclear magnetic resonance** (NMR) spectra were recorded in $CD_3CN$, DMSO-$d_6$ , DMF-$d_6$, TFA-$d_3$ or in their mixtures on an Agilent 400-MR DDR2 (399.94 MHz for $^1$H, 100.58 MHz for $^{13}$C), JEOL-ECZL400G (400 MHz for $^1$H, 101 MHz for $^{13}$C), or Bruker Avance III 600 MHz (600 MHz for $^1$H and 151 MHz for $^{13}$C) at 298 K. Data for $^1$H NMR are reported as follows: chemical shift (δ ppm), multiplicity (s = singlet, d = doublet, t = triplet, q = quartet, m = multiplet, dd = doublet of doublets, dt = doublet of triplets, br = broad etc.), coupling constant (Hz), and integration. All NMR spectra were processed and assigned using MestreNova. **High-resolution mass spectra** were obtained on Q-Tof Micro (Waters), equipped with a quadrupole and time-of-flight (TOF) analyser and a multichannel plate (MCP) detector. **GC-MS analysis** were performed on gas chromatograph Trace Ultra (Themo Scientific) with a quadrupole mass spectrometer DSQ II (Thermo Scientific) equipped with DB-5ms capillary column (30 m x 0.25mm, film 0,25 um) or on gas chromatograph 7890 (Agilent) with an orbital trap mass spectrometer Exploris 240 (Thermo Scientific) equipped with DB-5ms capillary column (30 m x 0.25mm, film 0,25 um). SICRIT (Soft Ionization by Chemical Reaction In Transfer - Plasmion GmbH, Germany) was used in positive mode. The **melting points** were measured on a Boetius melting point apparatus and are not corrected. **Photoredox catalysis** was performed using Luxeon Star LED 470 nm (65 lm @ 700mA) with dominant peak wavelengths 460–485 nm in tempered aluminium block (for arrangement, see S3) and Luxeon Star LED 505 nm (76 lm @ 350mA) with dominant peak wavelengths 490–515 nm. **UV-Vis absorption spectra** were recorded on a UV-2550 spectrophotometer (Schimadzu). **Steady-state emission spectra and fluorescence excitation spectra** were recorded on a Jobin Yvon-SpexFluorolog 3-22 spectrofluorometer. Note that spectra were recorded using the same excitation and emission slits for uniform spectral resolution and sensitivity. **Fluorescence lifetime measurements** of all compounds were performed using the time-correlated single-photon counting (TCSPC) method. Decays were measured with the TCSPC Triple Illuminator as an accessory for the Fluorologs 3-22 steady-state spectrofluorometer that adds lifetime-capability in the time domain. The excitation source were NanoLED diodes ($\lambda_{exc}$ = 368 nm and 389 nm) from IBH. The instrument in this hardware configuration is capable of measuring lifetimes as short as 400 ps. Deconvolution of fluorescence decay curves was performed using IBH Consultants software. The experimental uncertainties in the lifetimes are 10 %. **Setup for UV-Vis transient absorption spectra measurements**: The triplet states were generated and analysed using laser flash photolysis employing a nanosecond Q-switched Nd: YAG laser in combination with an optical parametric oscillator (OPO) operating at 470 nm; the laser pulse energy was kept around 0.5 mJ/pulse. The sample's solution was deoxygenated by bubbling argon for 15 min before measurements. Spectra were determined using kinetics collected between 350–700nm with a 10 nm interval. See ref.[1] for details. **Electrochemical measurements** (cyclic voltammetry) were managed by a computer driven potentiostat PGSTAT101 (Autolab-Metrohm) using NOVA 1.11 software, for details see Chapter 5.

**Quantum chemical calculations:** Geometries of all the structures were fully optimized to minima, confirmed by frequency calculations having no imaginary frequencies, using Gaussian 16 software[2] package employing a DFT method with a B3LYP functional in the calculation together with cc-pVTZ and aug-cc-pVTZ basis set. Acetonitrile as a solvent was approximated by CPCM method. Absorption spectra were calculated at the same level of theory using TD-DFT approach (the first 15 excited states were calculated). The optimized geometries were used for energy calculation. The energies were calculated at DFT-B3LYP level of theory together with cc-pVTZ and aug-cc-pVTZ basis set in Gaussian 16 software package. Acetonitrile as a solvent was simulated using CPCM approach. The NMR calculations were also performed using the NMR GIAO method at the same level of theory (DFT-B3LYP with cc-pVTZ and aug-cc-pVTZ basis set) and with acetonitrile as a solvent in the CPCM model

# S2. Synthesis and characterization of flavin derivatives

**3-Methyllumichrome (1a-H)**

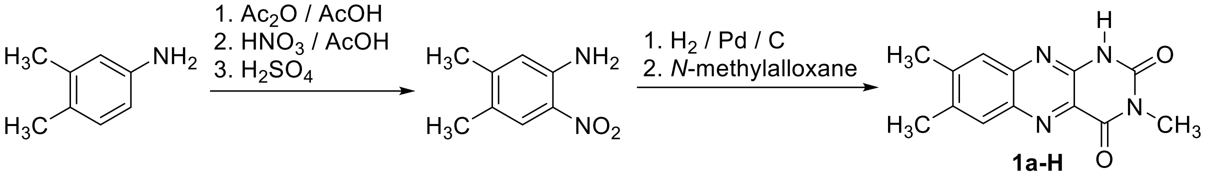


To the solution of 3,4-dimethylaniline (2.410 g, 19.88 mmol) in acetic acid (3.5 mL) acetic anhydride (3.5 ml, 31.74 mmol, 1.6 equiv.) was added. The reaction mixture was heated to reflux for 15 min and then added dropwise to water with ice. Precipitate (old pink color) was filtered off using Büchner funnel. The filtration cake was dissolved in acetic acid (6 mL) and added dropwise to the mixture of 65% nitric acid (12 ml, 266 mmol, 13 equiv.) and acetic acid (10 mL) cooled to +5 °C. The reaction mixture was allowed to warm to room temperature (90 min) and poured into water with ice (100 mL). Resulting precipitate was filtered off using Büchner funnel and washed with water (approximately 500 mL). After drying on air was obtained 2.337 g (56 %) of bright yellow crystals of 2-nitro-4,5-dimethylacetanilide (m.p. 99 – 103 °C, lit.[3] 101 - 104 °C)

$^{1}H$ NMR (400 MHz, $CDCl_3$) δ 10.29 (s, 1H), 8.53 (s, 1H), 7.97 (s, 1H), 2.34 (s, 3H), 2.28 (s, 3H), 2.27 (d, *J* = 0.7 Hz, 3H).

HRMS APCI pos m/z for $C_{10}H_{13}N_2O_3$ [M+H$^+$] calculated 209.09027, found 209.09158.

2-Nitro-4,5-dimethylacetanilide was dissolved in 96% sulfuric acid (15 mL) and heated to 90 °C for 30 min. After cooling to r.t., the reaction mixture was poured dropwise on ice. The precipitated orange crystals were filtered off using Büchner funnel and dried on air. It was obtained 1.380 g (74 %) of 3,4-dimethyl-6-nitroaniline, m.p. 136 – 139 °C, lit.[4] 140-141 °C.

$^{1}H$ NMR (400 MHz, $CDCl_3$) δ 7.87 (s, 1H), 6.59 (s, 1H), 2.22 (s, 3H), 2.18 (s, 3H).

HRMS APCI pos m/z for $C_8H_{11}N_2O_2$ [M+H$^+$] calculated 167.08150, found 167.08138.

3,4-Dimethyl-6-nitroaniline (1.380 g, 8.30 mmol) was dissolved in acetic acid (60 mL) and palladium on carbon (10%, 0.150 g) was added. Hydrogen (balloon) was bubbled through the reaction mixture until the color disappear (20 hours). Residual of palladium was filtered off through celite and the filtrate was added to the hot solution of *N*-methylalloxane (1.310 g, 8.39 mmol) and boric acid (0.620 g, 10.03 mmol) in acetic acid (60 mL). Reaction mixture was stirred in dark at r.t. for 4 hours. Bright yellow crystals were filtered off through frit (S4), washed with diethyl ether (10 ml) and dried on vacuo. It was obtained 1.270 g (60 %) of **1a-H**, m.p. 354 – 359 °C.

$^{1}H$ NMR (400 MHz, DMSO-*d*$_6$) δ 12.12 (s, 1H), 7.95 – 7.91 (m, 1H), 7.73 – 7.68 (m, 1H), 3.29 (s, 3H), 2.48 (d, *J* = 1.0 Hz, 3H), 2.47 – 2.44 (m, 3H).

$^{13}C$ NMR (101 MHz, DMSO-*D*$_6$) δ 160.46, 150.34, 145.16, 144.84, 141.78, 139.05, 138.58, 129.50, 128.75, 125.89, 27.74, 20.30, 19.67.

HRMS APCI pos m/z for $C_{13}H_{13}N_4O_2$ [M+H$^+$] calculated 257.10330, found 257.10336.

## 1-Methyllumichrome (3a-H)

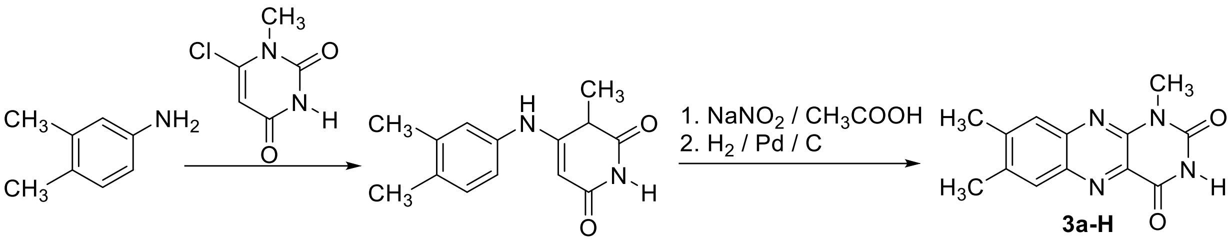


Mixture of 3,4-dimethylaniline (0.900 g, 5.60 mmol) and 1-methyl-6-chlorouracil (2.410 g, 19.90 mmol) was heated to 150 °C under stopper with calcium chloride for 2 hours. To the cooled mixture, diethyl ether (30 mL) was added and the mixture was sonicated. Product was filtered off, washed with diethyl ether, methanol and again with diethyl ether. Filtrate was evaporated, diethyl ether was added (100 mL) and precipitated crystals were filtered off. Crystals were collected. It was obtained 1.390 g (99 %) of 1-methyl-6-(3,4-dimethylanilino)uracil, m.p. 316 – 320 °C.
$^{1}$H NMR (400 MHz, DMSO-$d_6$) δ 10.62 (s, 1H), 7.20 (dd, *J* = 12.7, 8.1 Hz, 2H), 7.06 (d, *J* = 11.7 Hz, 3H), 7.03 (d, *J* = 2.3 Hz, 1H), 6.96 (dd, *J* = 7.9, 2.3 Hz, 1H), 4.39 (s, 1H), 2.22 (dd, *J* = 5.3, 2.8 Hz, 13H), 2.08 (s, 7H).
HRMS APCI pos m/z for $C_{13}H_{16}N_3O_2$ [M+H$^+$] calculated 246.12370, found 246.12352.

To the mixture of anilinouracil (1.390 g, 5.60 mmol) and acetic acid (30 mL), sodium nitrite (1.89 g, 26.40 mmol) was added. The mixture was stirred at r.t. for 2 hours and after addition of water (20 mL), the mixture was stirred 10 minutes. Precipitated crystals were filtered off through Büchner funnel and washed with diethyl ether. It was obtained 0.990 g of orange crystals as mixture of alloxazine and alloxazinium-*N*-oxide (3:1, according $^{1}$H NMR).
Part (0.275 g) of the mixture of alloxazin and alloxazinium-*N*-oxide was dissolved in the mixture of solvents DMF (8 mL) and acetic acid (45 mL). Palladium on carbon was added (10%, 70 mg) and the mixture was stirred under hydrogen (6 atm) in autoclave at r.t. for 46 hours. The reaction mixture was heated until dissolving all solids and palladium on carbon was filtered off. After evaporation of solvents, it was obtained 0.255 g (64 %) of **3a-H**, m.p. 338-342 °C, lit.[5] >300 °C.
$^{1}$H NMR (400 MHz, $CD_3CN$ / DMSO-$d_6$) δ 11.49 (s, 1H), 7.93 (d, *J* = 1.3 Hz, 1H), 7.77 (s, 1H), 3.59 (s, 3H), 2.51 (d, *J* = 1.0 Hz, 3H), 2.48 (d, *J* = 1.1 Hz, 3H).
$^{1}$H NMR (600 MHz, DMSO-$d_6$) δ 7.93 (d, *J* = 1.2 Hz, 1H), 7.80 (d, *J* = 1.2 Hz, 1H), 3.52 (s, 3H), 2.48 (s, 3H), 2.46 (d, *J* = 1.0 Hz, 3H).
$^{13}$C NMR (151 MHz, DMSO-$d_6$) δ 159.73, 150.31, 146.61, 144.85, 141.00, 139.22, 137.60, 130.73, 128.56, 126.33, 28.27, 20.21, 19.62.
HRMS APCI pos m/z for $C_{13}H_{13}N_4O_2$ [M+H$^+$] calculated 257.10330, found 257.10294.

## Lumiflavin (7,8,10-trimethylisoalloxazine, 4a-H)

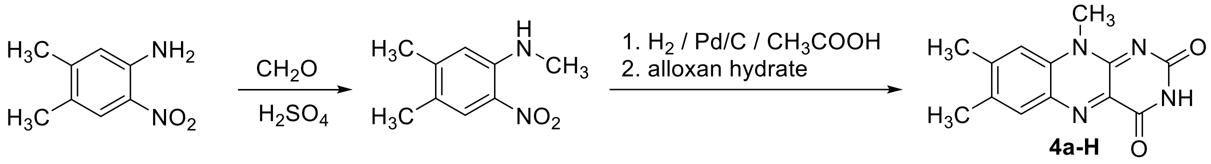


Lumiflavin **4a-H** was obtained starting from 3,4-dimethyl-6-nitroaniline using procedure described in literature.[6]

M.p. > 370 °C.

$^{1}H$ NMR (400 MHz, $CD_3CN$) δ 10.73 (s, 1H), 7.88 (s, 1H), 7.64 (s, 1H), 3.98 (s, 3H), 2.52 (s, 3H), 2.41 (s, 3H).

$^{13}C$ NMR (151 MHz, $CD_3CN$ / DMSO-$d_6$) δ 161.07, 156.67, 154.38, 138.10, 134.91, 132.87, 132.16, 117.09, 32.62, 21.12, 19.24.

HRMS APCI pos m/z for $C_{13}H_{13}N_4O_2$ [M+H$^+$] calculated 257.10330, found 257.10355.

## 1,3-Dimethyllumichrome (5a)

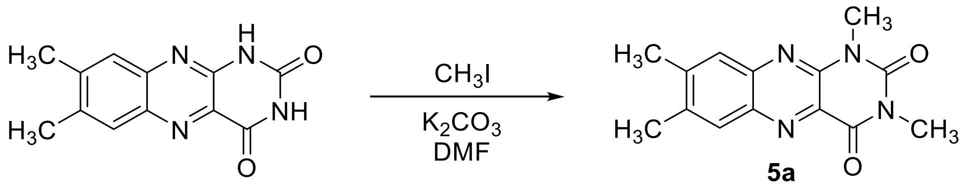


Lumichrome (0.080 g, 0.33 mmol) was dissolved in dry DMF (4 mL), potassium carbonate (0.300 g, 2.17 mmol) and methyl iodide (0.270 g, 0.525 mmol) were added. The mixture was stirred under argon atmosphere for 2 hours, then poured into water and extracted with ethyl acetate. Combined extracts were washed with water, brine and dried over magnesium sulfate. After evaporation of solvent was obtained 0.090 g (99 %) of 1,3-dimethyllumichrome, m.p. 242 – 245 °C, lit.[7] 246 – 248 °C.

$^{1}H$ NMR (400 MHz, $CD_3CN$ / DMSO-$d_6$) δ 7.94 (d, $J$ = 1.3 Hz, 1H), 7.79 – 7.76 (m, 1H), 3.68 (d, $J$ = 0.5 Hz, 3H), 3.43 (d, $J$ = 0.5 Hz, 3H), 2.52 (d, $J$ = 1.0 Hz, 4H), 2.51 – 2.48 (m, 4H).

$^{13}C$ NMR (151 MHz, $CD_3CN$ / DMSO-$d_6$) δ 160.84, 151.81, 146.53, 146.47, 142.74, 140.77, 139.44, 130.56, 129.68, 127.35, 29.67, 29.09, 20.75, 20.09.

HRMS APCI pos m/z for $C_{14}H_{15}N_4O_2$ [M+H$^+$] calculated 271.11895, found 271.11838.

## Lumichrome

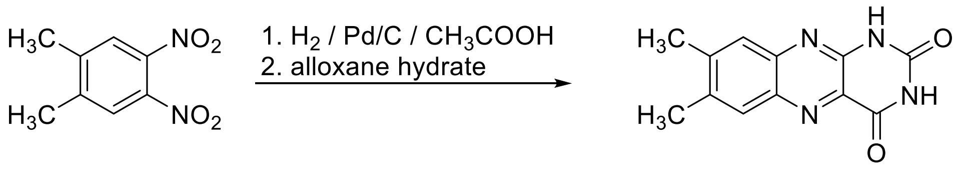


To the mixture of 1,2-dimethyl-4,5-dinitrobenzene (0.600 g, 3.06 mmol) in acetic acid (20 mL), palladium on carbon (10%, 160 mg) was added. The mixture was stirred under hydrogen atmosphere (balloon) for 24 hours. Palladium on carbon was filtered off through celite, washed with acetic acid and filtrate was immediately added to the hot solution of alloxane hydrate (0.490 g, 3.06 mmol, 1 equiv.) and boric acid (0.208 g, 3.36 mmol, 1.1 equiv.) in acetic acid (30 mL). The mixture was stirred at r.t. in

dark overnight. Precipitated orange crystals were filtered off through frit (S4), washed with acetic acid and diethyl ether. It was obtained 0.580 g (78 %) of lumichrome, m.p. 358 - 362°C., lit.[7] 368 – 370 °C.
$^{1}H$ NMR (400 MHz, $CD_3CN$ / DMSO-$D_6$) δ 11.32 (s, 2H), 7.90 (s, 1H), 7.69 (s, 1H), 2.50 (s, 3H), 2.47 (d, $J$ = 1.0 Hz, 3H).
$^{13}C$ NMR (151 MHz, $CD_3CN$ / DMSO-$D_6$) δ 161.84, 151.13, 147.53, 146.19, 143.09, 140.38, 139.87, 130.99, 129.81, 126.94, 20.68, 20.04.
HRMS APCI pos m/z for $C_{12}H_{11}N_4O_2$ [M+H$^+$] calculated 243.08765, found 243.08786.

# S3. Photoredox catalysis - experimental

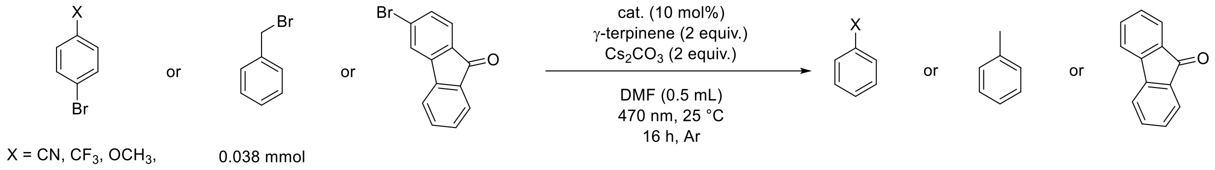


## Dehalogenation – inert conditions

Substrate (0.038 mmol), alloxazine or flavin derivative (10 mol%; 4×10$^{-3}$ mmol), γ-terpinene (2 equiv.; 0.076 mmol), and $Cs_2CO_3$ (2 equiv.; 0.076 mmol) were suspended in DMF (0.5 mL), placed into vial or Schlenk tube and bubbled with argon or degassed using the freeze-pump-thaw technique (3 × 5 min), respectively. The reaction mixture was stirred and irradiated with LED 470 nm (65 lm @ 700mA) or LED 505 nm for 3-bromo-*9H*-fluoren-9-one at 25 °C for 16 h. Conversion was determined by GC-MS.

## Dehalogenation – under air

4-Bromobenzonitrile (0.038 mmol), alloxazine derivative (10 mol%; 4×10$^{-3}$ mmol), γ-terpinene (2 equiv.; 0.076 mmol), and $Cs_2CO_3$ (2 equiv.; 0.076 mmol) were suspended in DMF (0.5 mL) and placed into vial. The reaction mixture was stirred and irradiated with LED 470 nm (65 lm @ 700mA) at 25 °C for 16 h. Conversion was determined by GC-MS.

## Dehalogenation – other bases

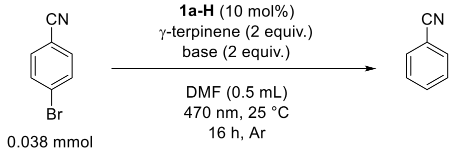


4-Bromobenzonitrile (0.038 mmol), **1a-H** (10 mol%; 4×10$^{-3}$ mmol), γ-terpinene (2 equiv.; 0.076 mmol), and CsOAc or $TBAH_2PO_4$ (2 equiv.; 0.076 mmol), were dissolved/suspended in DMF (0.5 mL), placed into vial or Schlenk tube and bubbled with argon or degassed using the freeze-pump-thaw technique (3 × 5 min), respectively. The reaction mixture was stirred and irradiated with LED 470 nm (65 lm @ 700mA) at 25 °C for 16 h. Conversion was determined by GC-MS.

## Coupling with $P(OMe)_3$ – inert conditions

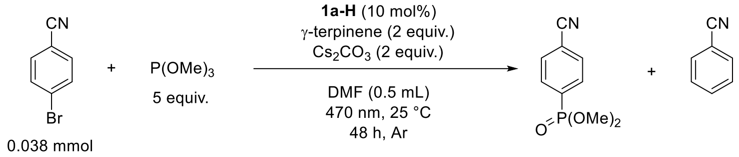


4-Bromobenzonitrile (0.038 mmol), trimethyl phosphite (5 equiv.; 0.190 mmol), **1a-H** (10 mol%; 4×10$^{-3}$ mmol), γ-terpinene (2 equiv.; 0.076 mmol), and $Cs_2CO_3$ (2 equiv.; 0.076 mmol), were suspended in DMF (0.5 mL), placed into vial or Schlenk tube and bubbled with argon or degassed using the freeze-pump-thaw technique (3 × 5 min). The reaction mixture was stirred and irradiated at 470 nm (65 lm @ 700mA) at 25 °C for 48 h. Conversion was determined by GC-MS.

## Coupling with $P(OMe)_3$ – under air

4-Bromobenzonitrile (0.038 mmol), trimethyl phosphite (5 equiv.; 0.190 mmol), **1a-H** (10 mol%; $4\times10^{-3}$ mmol), $\gamma$-terpinene (2 equiv.; 0.076 mmol), and $Cs_2CO_3$ (2 equiv.; 0.076 mmol) were suspended in DMF (0.5 mL) and placed into vial. The reaction mixture was stirred and irradiated at 470 nm (65 lm @ 700mA) at 25 °C for 48 h. Conversion was determined by GC-MS.

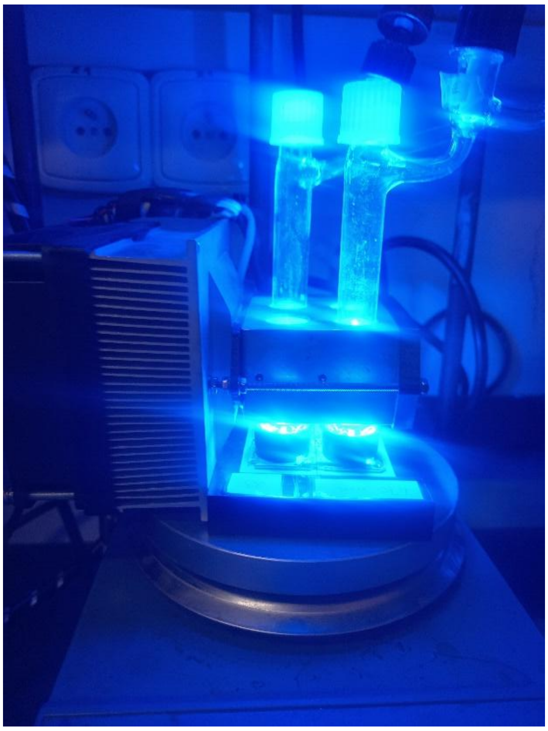

**FigureS3.1 T**ypical set-up of photochemical experiment

# S4. UV-Vis, fluorescence and excitation spectra, fluorescence lifetime and UV-Vis transient absorption spectra measurements

Anions of 3-methyllumichrome (**1a-H**), 1-methyllumichrome (**3a-H**) and lumiflavin (**4a-H**) were obtained *in situ*, adding salt tetrabutylammonium acetate (TBAOAc). TBAOAc was of ≥ 99% of purity and was from Sigma-Aldrich. Before use, TBAOAc was dried under vacuum and stored in a desiccator. Flavin derivatives were dissolved in dry acetonitrile (ACN). Only freshly prepared solutions were used in order to minimize possible photolytic reactions or hydrolysis. All experiments were carried out at room temperature.

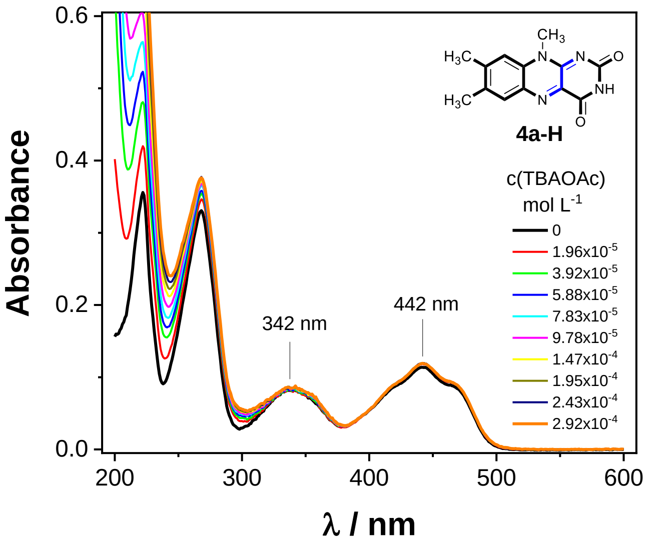


**Figure S4.1**. The absorption spectra of lumiflavin (**4a-H**) in ACN ($8.49×10^{-6}$ M) with TBAOAc.

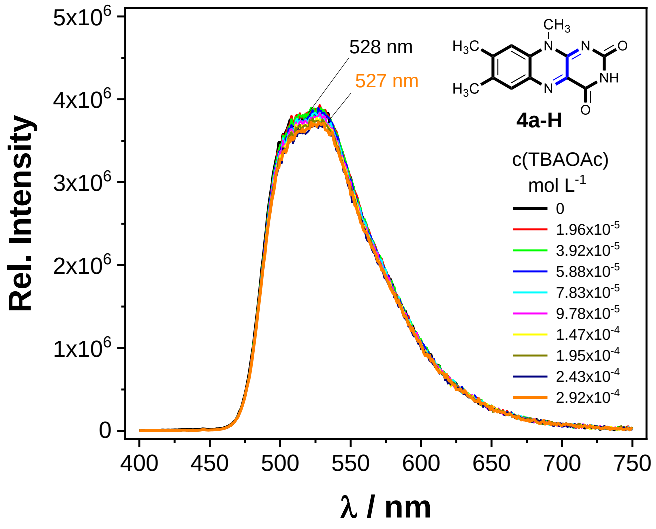


**Figure S4.2.** The emission spectra of lumiflavin (**4a-H**) in ACN ($8.49×10^{-6}$ M) with TBAOAc, excited at λ = 393 nm.

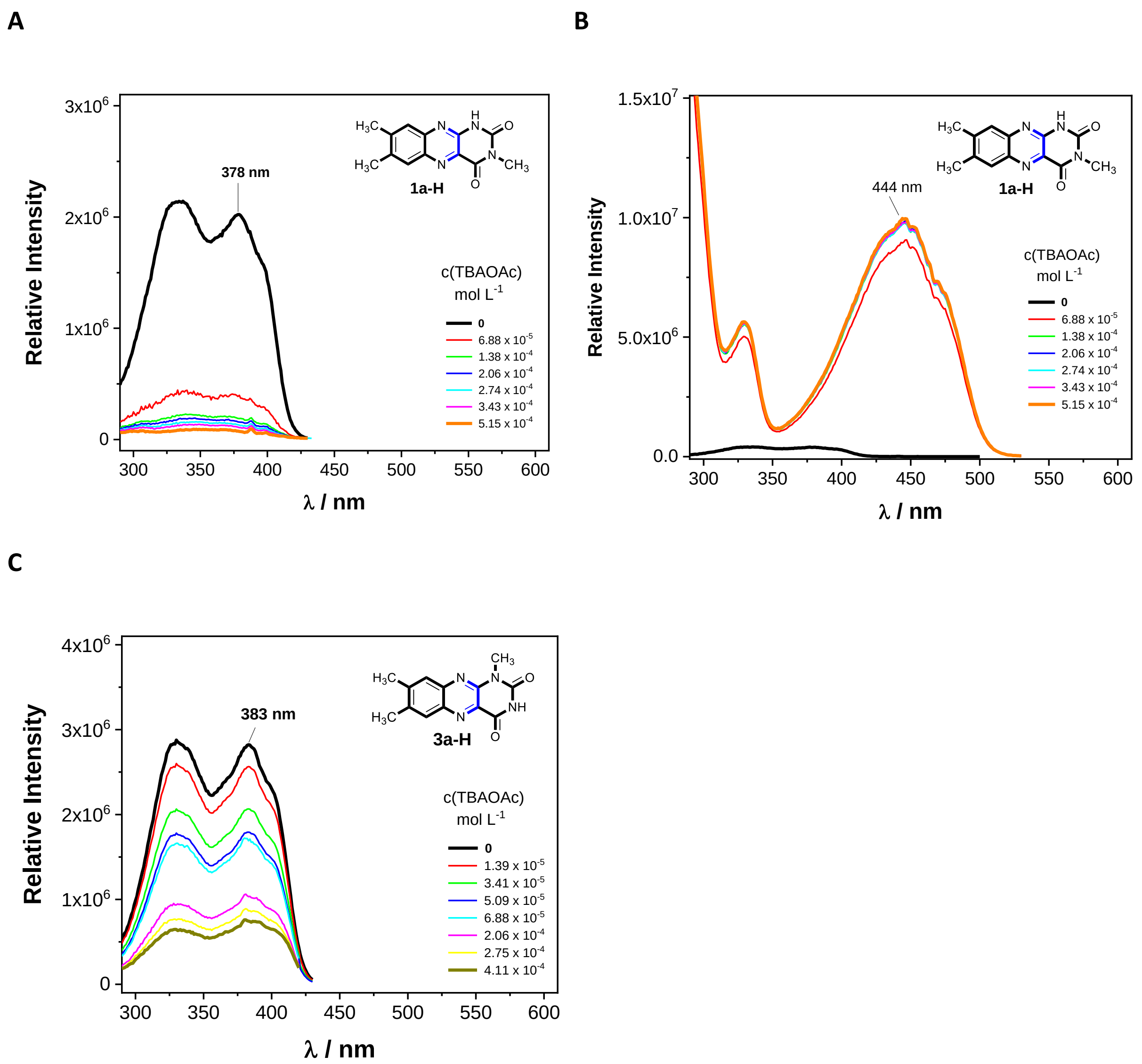


**Figure S4.3.** Fluorescence excitation spectra of (A) **1a-H** ($8.49\times10^{-6}$ M) with TBAOAc (observed at $\lambda_{Em}$ = 440 nm), (B) **1a-H** ($8.49\times10^{-6}$ M) with TBAOAc (observed at $\lambda_{Em}$ = 540 nm), and (C) **3a-H** ($1.24\times10^{-5}$ M) with TBAOAc (observed at $\lambda_{Em}$ = 450 nm).

## Fluorescence lifetime quenching

The measurements were conducted in the presence of varying concentrations of the quencher. Excitation was achieved using a LED diode with a nominal wavelength of 455 nm (actual wavelength 449nm) and fluorescence emission was monitored at 540 nm. The data illustrate the effect of quencher concentration on the fluorescence lifetime of **1a-H**.

**Table S4.1** Fluorescence lifetime measurement of **1a-H** in dimethylformamide (DMF) containing tetrabutylammonium acetate (TBAOAc, 29 mM) and 4-bromobenzonitrile (**6a**) in different concentrations.

| c(**6a**) [M] | τ [ns] |
|---|---|
| **0** | **8.66** |
| 0.076 | 8.62 |
| 0.092 | 8.58 |
| 0.112 | 8.49 |
| 0.131 | 8.46 |
| 0.168 | 8.45 |
| 0.201 | 8.43 |
| 0.232 | 8.38 |
| 0.261 | 8.37 |
| 0.288 | 8.33 |

$\tau_0/\tau = 1 + k_q\tau_0[Q]$

$1/\tau = 1/\tau_0 + k_q[Q]$

$\mathbf{k_q = 1.64\times10^{7} M^{-1}s^{-1}}$

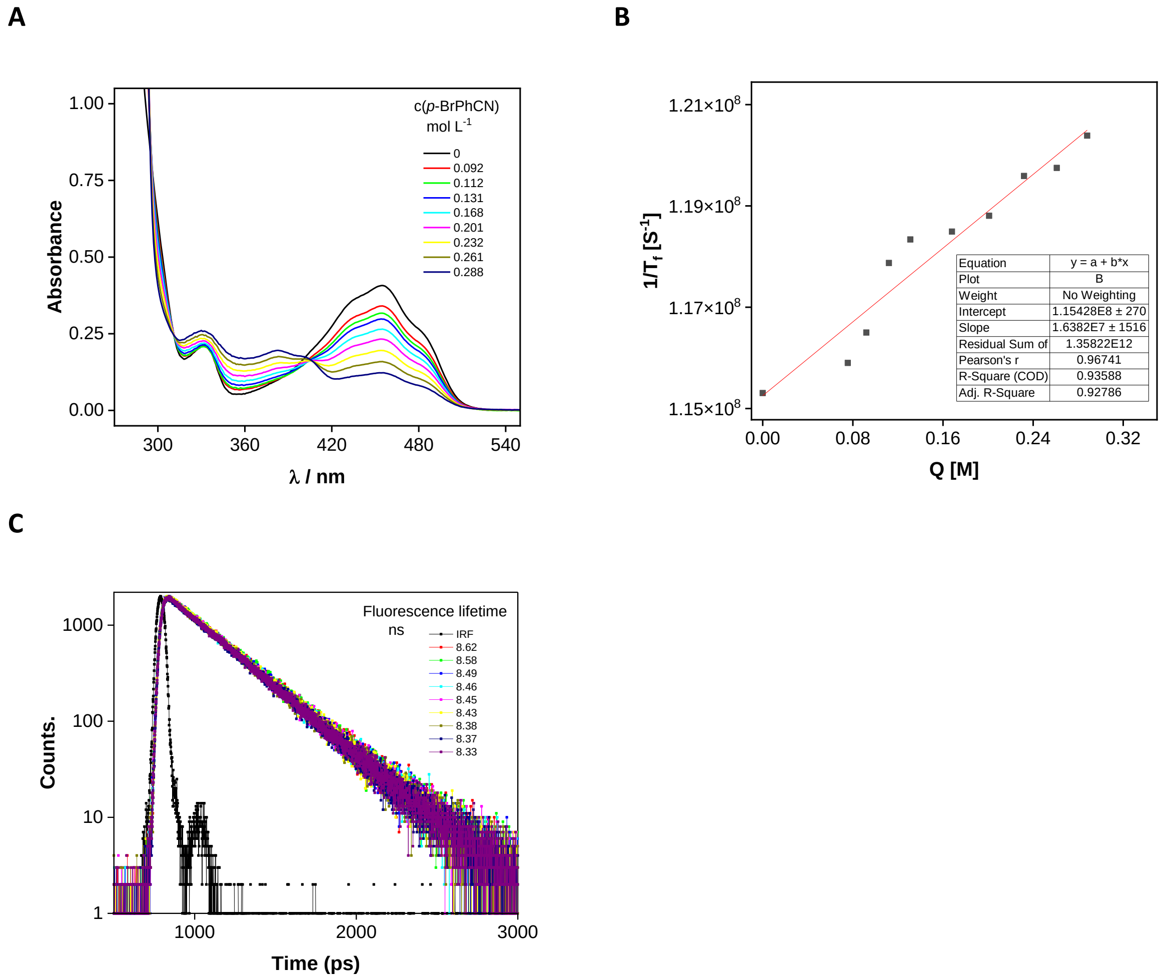


**Figure S4.4.** Absorption spectra of **1a-H** in DMF in the presence of TBAOAc (29 mM) at different concentration of 4-bromobenzonitrile (**6a**) as a quencher (A). Linear plot of $1/\tau_f$ versus quencher concentration [Q] (B). Fluorescence decay of **1a-H** in the presence of TBAOAc (29 mM) at different concentration of **6a** as a quencher (C).

**Table S4.2** Fluorescence lifetime measurement of **1a-H** in dimethylformamide (DMF) containing tetrabutylammonium acetate (TBAOAc, 29 mM) and 1-bromo-4-nitrobenzene (**6f**) at different concentrations.

| c(**6f**) [M] | τ [ns] |
|---|---|
| **0** | **8.48** |
| 3.32 | 7.48 |
| 6.60 | 6.63 |
| 9.85 | 5.92 |
| 13.07 | 5.40 |
| 16.26 | 4.91 |
| 19.42 | 4.56 |
| 22.54 | 4.24 |
| 25.64 | 3.96 |
| 28.71 | 3.71 |
| 31.75 | 3.50 |
| 60.61 | 2.32 |

$\tau_0/\tau = 1 + k_q\tau_0[Q]$

$1/\tau = 1/\tau_0 + k_q[Q]$

$\mathbf{k_q = 5.21\times10^9 M^{-1}s^{-1}}$

**A**

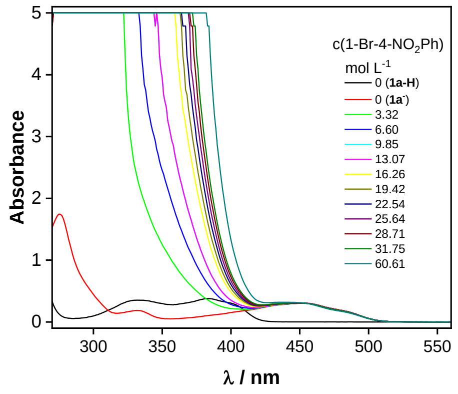


**B**

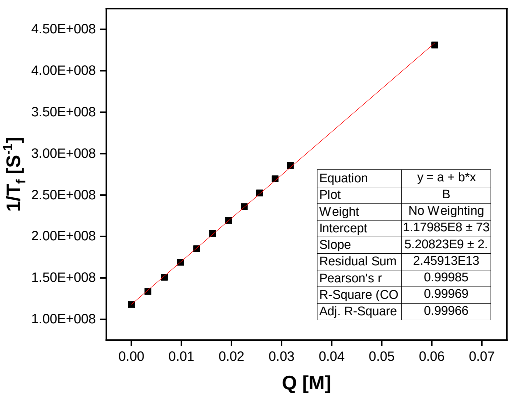


**C**

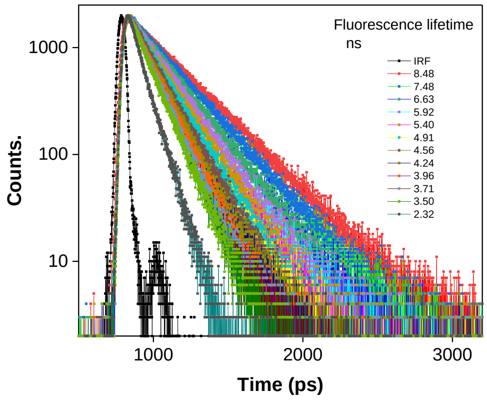


**Figure S4.5.** Absorption spectra of **1a-H** in DMF in the presence of TBAOAc (29 mM) at different concentration of 1-bromo-4-nitrobenzene (**6f**) as a quencher (A). Linear plot of $1/\tau_f$ versus quencher concentration [Q] (B). Fluorescence decay of **1a-H** in the presence of TBAOAc (29 mM) at different concentration of **6f** as a quencher (C).

**Table S4.3** Fluorescence lifetime measurement of **1a-H** in dimethylformamide (DMF) containing tetrabutylammonium acetate (TBAOAc, 29 mM) and 1-bromo-9-H-fluoren-9-one (**6e**) in different concentrations.

| c(**6e**) [M] | τ [ns] |
|---|---|
| **0** | **8.48** |
| 3.32 | 7.42 |
| 6.60 | 6.58 |
| 9.85 | 5.93 |
| 13.07 | 5.46 |
| 16.26 | 5.06 |
| 19.42 | 4.70 |
| 22.54 | 4.37 |
| 25.64 | 4.28 |
| 28.71 | 3.92 |
| 31.75 | 3.73 |
| 60.61 | 2.57 |

$\tau_0/\tau = 1 + k_q\tau_0[Q]$

$1/\tau = 1/\tau_0 + k_q[Q]$

$\mathbf{k_q = 4.47\times10^9 M^{-1}s^{-1}}$

**A**

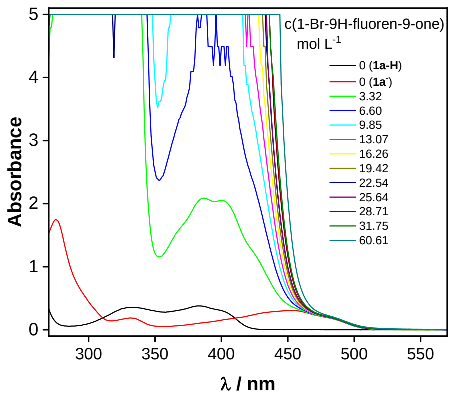


**B**

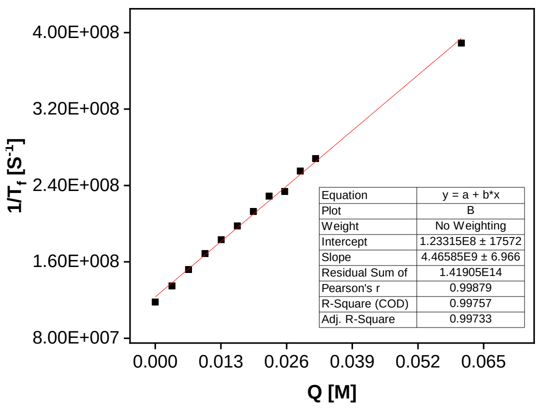


**C**

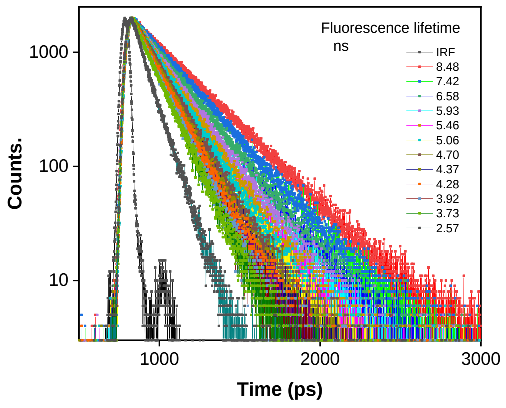


**Figure S4.6.** Absorption spectra of **1a-H** in DMF in the presence of TBAOAc (29 mM) at different concentration of 1-bromo-9-H-fluorene-9-one (**6e**) as a quencher (A). Linear plot of $1/\tau_f$ versus quencher concentration [Q] (B). Fluorescence decay of **1a-H** in the presence of TBAOAc (29 mM) at different concentration of **6e** as a quencher (C).

## Triplet lifetime quenching in DMF

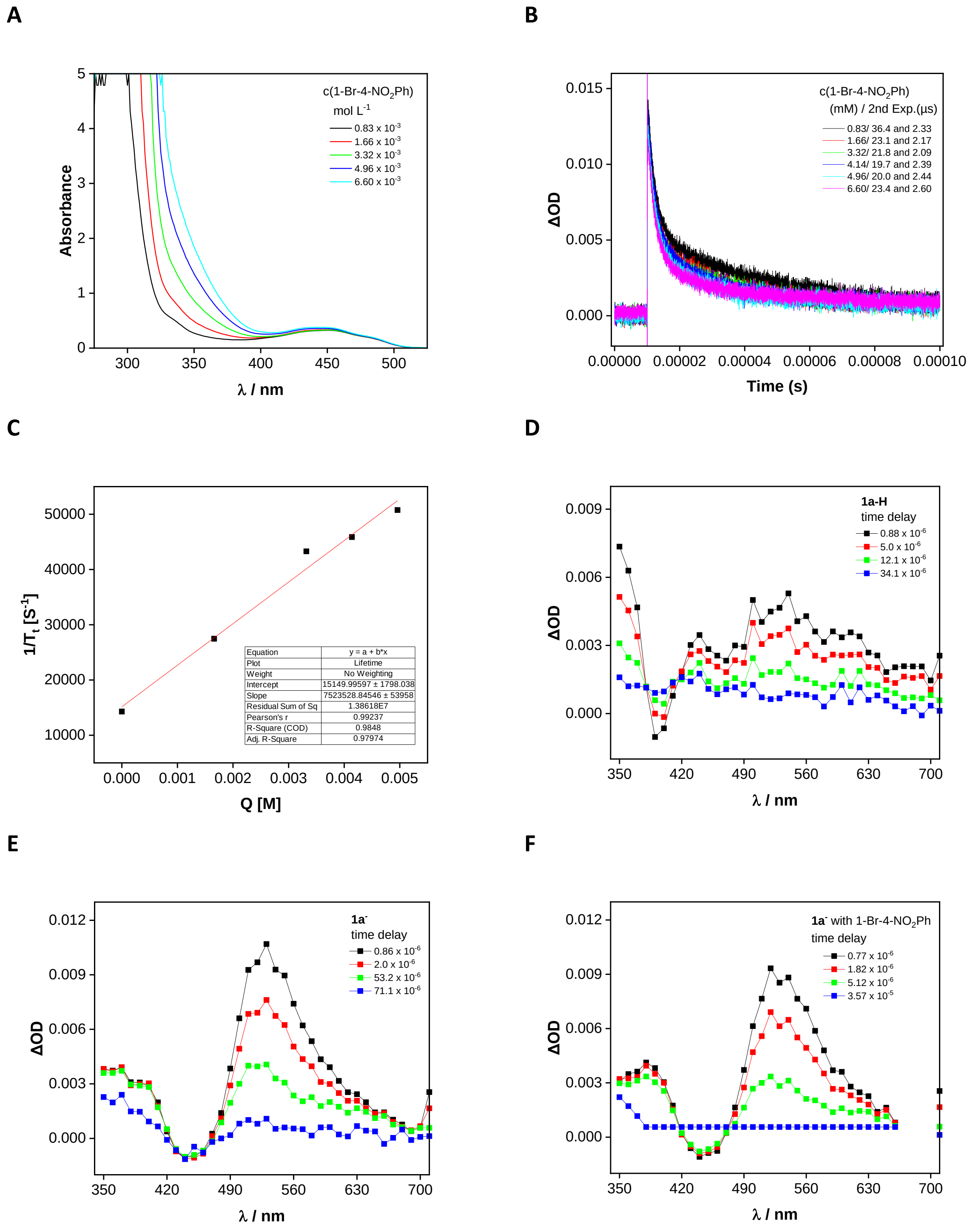


**Figure S4.7.** Absorption spectra of **1a-H** in the presence of TBAOAc (29 mM) at varying concentration of 1-bromo-4-nitrobenzene (**6f**) (A). Triplet decay of **1a-H** in the presence of TBAOAc at different concentration of **6f**, monitored at 530nm with laser excitation at 470 nm. Notably, multiexponential decay is due to triplet-triplet annihilation in the first microseconds followed by triplet population decay over tens of microseconds. (B). Linear plot of $1/\tau_t$ versus quencher **6f** concentration [Q] (C). The transient absorption spectra of **1a-H** at different time delays, wavelength of excitation 405 nm (D). The transient absorption spectra of **1a-H** with TBAOAc at different time delays, wavelength of excitation 470 nm (E). The transient absorption spectra of **1a-H** with TBAOAc and 1-bromo-4-nitrobenzene (**6f**) at different time delays, wavelength of excitation 470 nm (F).

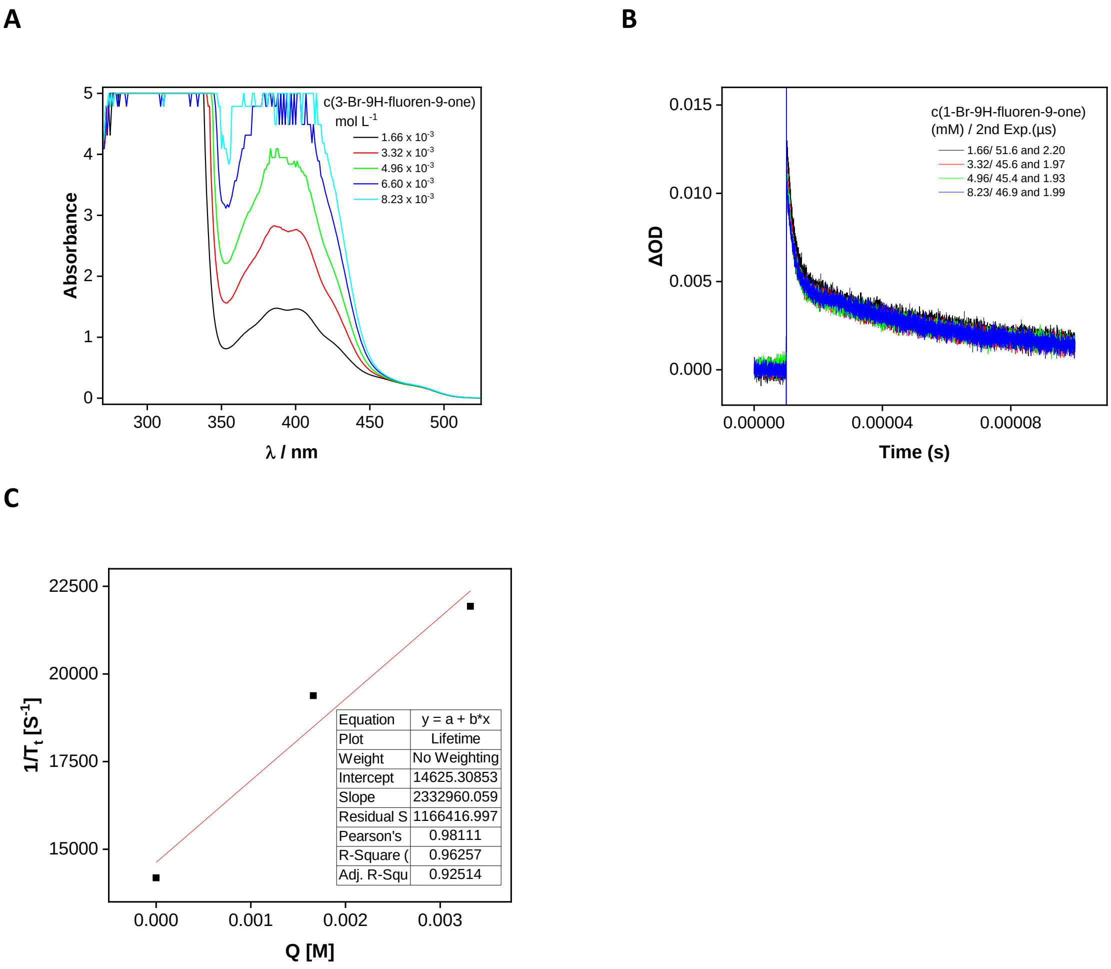


**Figure S4.8.** Absorption spectra of **1a-H** in the presence of TBAOAc (29 mM) at varying concentration of 1-bromo-9H-fluorene-9-one (**6e**) (A). Triplet decay of **1a-H** with TBAOAc at different concentration of **6e**, monitored at 530nm with laser excitation at 470 nm. Notably, multiexponential decay is due to triplet-triplet anihilation in the first microseconds followed by triplet population decay over tens of microseconds. (B). Linear plot of $1/\tau_t$ versus quencher **6e** concentration [Q] (C).

## Triplet lifetime of anion formed from 1a-H in DMF under argon and air

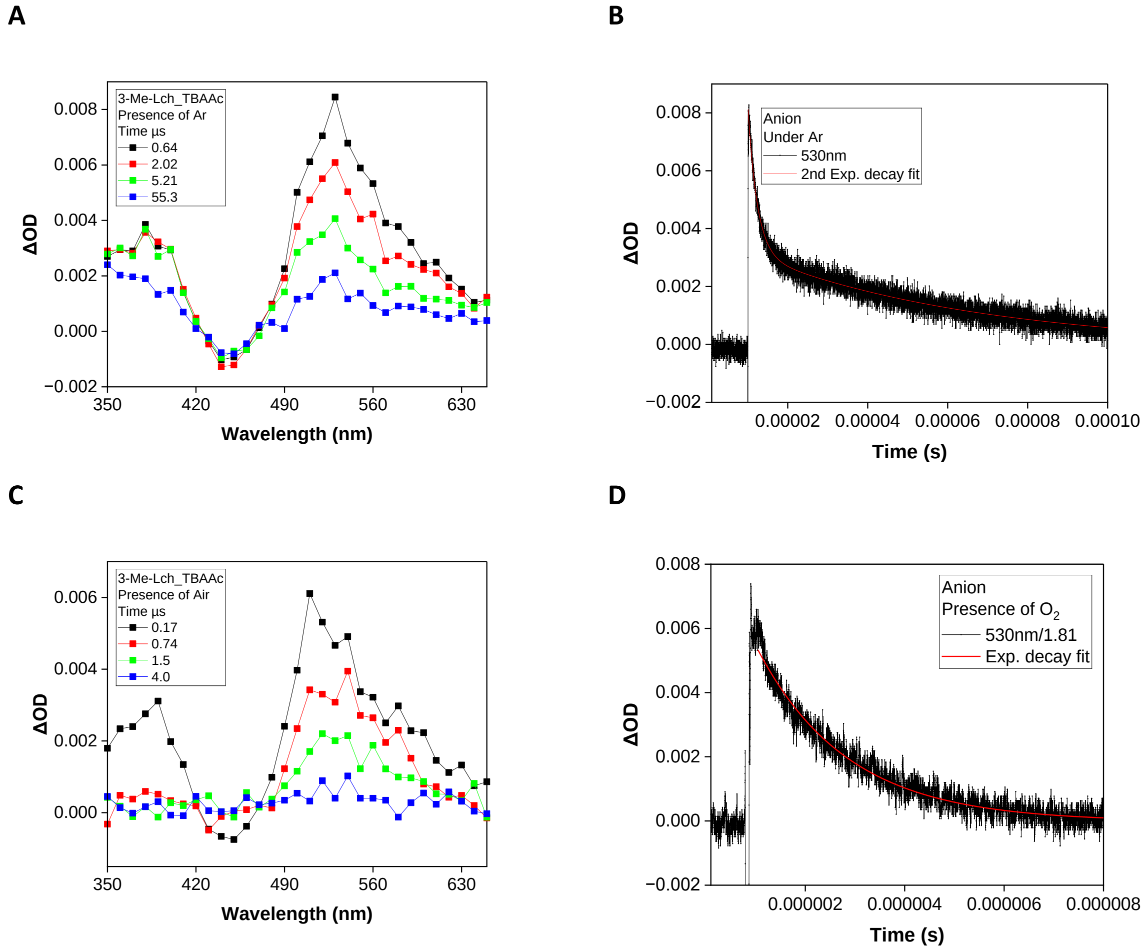


**Figure S4.9.** Transient absorption spectra of **1a-H** with TBAOAc (29 mM) **under argon** (A) and triplet decay at 530 nm with laser excitation at 470 nm (B); $\tau_T = 57.3$ µs (short component of 2.32 µs is due to triplet-triplet annihilation). Transient absorption spectra of **1a-H** with TBAOAc (29 mM) **under air** (C) and triplet decay at 530 nm with laser excitation at 470 nm; $\tau_T = 1.81$ µs (D). Laser power energy 0.5 mJ.

## Experiments aimed at triplet anion photophysics characterization: $\Phi_{ISC}$, $\varepsilon_{max}(T_1, 520\ nm)$

### Relative actinometry

Comparative experiment using $Ru(bpy)_3^{2+}$ was performed for two solutions:

sample 1: solution of anion in DMF

sample 2: solution of $Ru(bpy)_3^{2+}$ in $H_2O$

Both samples were prepared with the same absorption at excitation wavelength:

A(470 nm, 1 cm)=0.30.

Transient absorption experiment was executed in row: sample 1, sample 2. The laser pulse energy 0.5 mJ. The obtained initial amplitudes signals are:

$\Delta$A(Ru(bpy)$_3^{2+}$, 450 nm) = - 0.01866 (dominated by $S_0$ bleaching)

$\Delta$A(anion, 520 nm) = 0.0069 (the band peak of triplet-triplet anion absorption).

Accordingly with literature[8] for Ru(bpy)$_3^{2+}$, the difference in the epsilon values at 450 nm of the triplet excited state and ground state state is
$\Delta\varepsilon$( Ru(bpy)$_3^{2+}$, 450nm) = -1.0 $\times 10^4$ M$^{-1}$cm$^{-1}$ (accuracy 9 %).

This allows us to estimate the $\varepsilon_{T1}$(anion, 520 nm)$\times\Phi_{ISC}$ from following equation:

$$\varepsilon_{T1}(anion, 520\,nm) \times \Phi_{ISC} = \frac{\Delta A(anion\,T_1, 520\,nm) \times \Delta\varepsilon(Ru(bpy)_3^{2+}, 450\,nm)}{\Delta A(Ru(bpy)_3^{2+}, 450\,nm)} \approx 3700\,M^{-1}cm^{-1}$$

With accuracy of about 15 %.

Since anion fluorescence quantum yield $\Phi_f$ is 0.26, it is clear that expected value $\Phi_{ISC}$ is, at best, limited to 1-0.26 = 0.74, thus $\varepsilon_{T1}(anion, 520\,nm) \geq \frac{3700}{0.74} = 5000\,M^{-1}cm^{-1}$.

**Triplet-triplet energy transfer from anion-$T_1$ to retinol-$T_1$**

Experiment was performed for the mixture anion and retinol in DMF, deoxygenated by 15 min Ar bubbling. As the excitation wavelength 470 nm was chosen for a selective excitation of the anion (retinol shows an absorption band peaking at 329 nm in DMF).

In transient absorption experiment, at delay of 300 ns a positive band is observed, peaking at 520 nm, corresponding to $T_1$ state of anion ($T_1 \rightarrow T_n$, Figure 1A).

(A) (B)

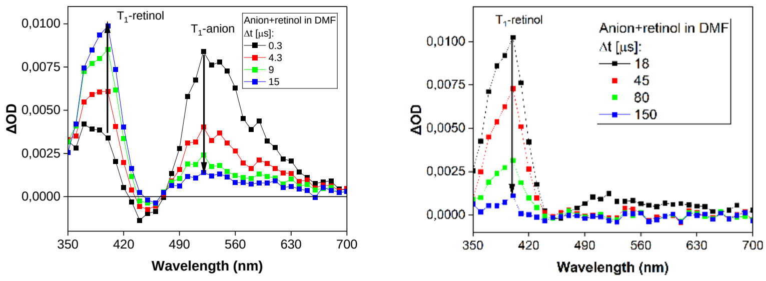


Figure 1. (A) *UV-vis transient absorption spectra determined for mixture of anion and retinol in DMF deoxygenated solution in time windows (A) 300 ns–15 μs and (B) 18 –150 μs. Excitation at 470 nm, 0.5 mJ per pulse, c(anion) = 1.3 $\times 10^{-4}$ M, c(retinol) = 2.8 $\times 10^{-5}$ M.*

In time-window from 300 ns to 15 μs (Figure 1A) a decay of population of anion in the $T_1$ state takes place, and the rise of the band peaking at 400 nm, corresponding to retinol in the $T_1$ triplet state, occurs. Triplet-triplet energy takes place. Over a longer time-window, from 18 to 150 μs (Figure 1B), positive band at 400 nm undergoes a decay. It is related to decay of retinol population in the $T_1$ state.

Triplet-triplet energy transfer from anion to retinol is also confirmed by shortening of the triplet state lifetime of anion upon going from a pure deoxygenated anion solution ($\tau$ = 32.3 μs, amplitude averaged lifetime) to the mixture of anion with retinol ($\tau$ = 6.8 μs, amplitude averaged lifetime). Amplitude

averaged lifetimes ($\tau = \frac{A_1\tau_1 + A_2\tau_2}{A_1 + A_2}$) were calculated to take into account a biexponential character of the kinetics.

To estimate the efficiency $\Phi_{T\to T}$ for triplet-triplet energy process:

$\Phi_{T\to T} = k_{T\to T}/ (k_{T\to T} + k_0) = 0.82$

Where:

the rate $k_0$ corresponds to the decay of triplet of anion in a pure argon-saturated DMF

($k_0$=1/32.3 μs), and

$k_{T\to T}$ is the rate of the triplet-triplet energy transfer in the presence of retinol

($k_{T\to T} + k_0$=1/6.8 μs).

Let us estimate the molar absorption coefficient $\varepsilon_{T1}(anion, 520\ nm)$.

The kinetics at 520 nm shows a decrease in ΔA amplitude by 0.0084 (triplet anion population, Figure 1A). This is accompanied by the rise of band at 400 nm with amplitude of 0.00987 (triplet retinol population).

The molar absorption coefficient for retinol in the triplet state is ε(retinol $T_1$, 400nm) ≈ 6.6 ×$10^4$ $M^{-1}cm^{-1}$ (see ref.[9]).

These findings lead us to following equation:

$$\varepsilon_{T1}(anion, 520\ nm) = \frac{\varepsilon(\text{ retinol } T_1, 400\text{ nm}) \times \Delta A(anion\ T_1, 520\ nm)\ \times \Phi_{T\to T}}{\Delta A(retinol\ T_1, 400\ nm)}$$
$$= \frac{6.6 \times 10^4 \times 0.0084 \times 0.82}{0.00987} \approx 46000\ M^{-1}cm^{-1}$$

Since the comparative experiment lead to

$$\varepsilon_{T1}(anion, 520\ nm) \times \Phi_{ISC} \approx 3700\ M^{-1}cm^{-1}$$

**The resulting $\boldsymbol{\Phi_{ISC}}$ is close to 0.08**.

The accuracy in that estimation is not high, taking into account all assumptions undertaken. Further studies should be performed to bring us more accurate characterization of photophysics for the triplet anion.

# S5. CV measurements

An undivided 10 ml cell with three-electrode system: work - GCE ø 3 mm, reference - SCE, auxiliary - Pt plate, scan rate 100 $mV \cdot s^{-1}$ in ACN deoxygenated by argon. 0.1 mol $L^{-1}$. Tetrabutylammonium hexafluorophosphate ($TBAPF_6$) as the supporting electrolyte; samples concentrations 0.5 x $10^{-3}$ mol $L^{-1}$. The concentration of added base change from $0.25 \cdot 10^{-3}$ mol $L^{-1}$ to $25 \cdot 10^{-3}$ mol $L^{-1}$. The maximum concentration of added base (TBAOAc, respective $TBAH_2PO_4$, $25 \cdot 10^{-3}$ mol $L^{-1}$) correspond to 50 equivalents.

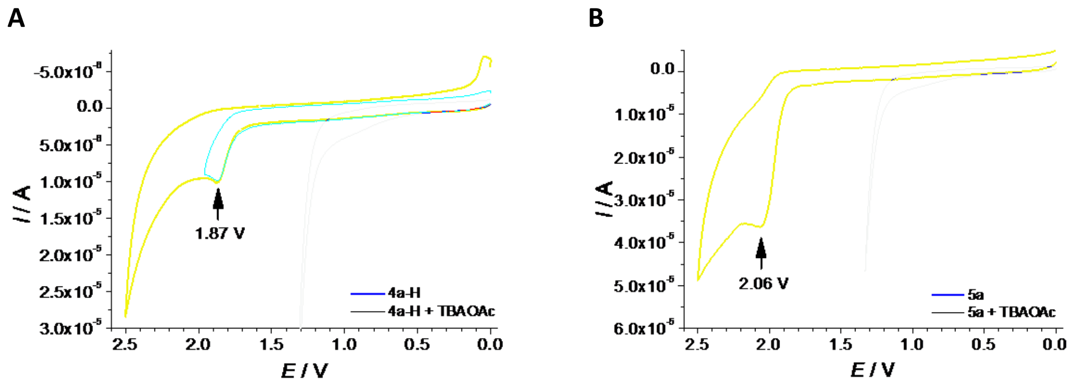


**Figure S5.1** Cyclic voltammograms of (A) **4a-H** and (B) **5a** in the absence and in the presence of TBAOAc (50 equiv.). Data obtained in acetonitrile in the presence of $TBAPF_6$ ($c$ = 0.1 mol $L^{-1}$) using glassy carbon working, saturated calomel reference and platinum auxiliary electrodes.

# S6. Estimation of $E^*_{ox}$ of **2a**⁻ in singlet excited state

$E^*_{ox}$ of **2a**$^-$ in single excited state was estimated from ground state redox potential ($E_{p/2}$ = 1.02 V; i.e. value from CV measurement of **1a-H** in the presence of TBAOAc) and the value $E^{0-0}$ = 2.52 eV corresponding to intersection of the normalized absorption and emission bands (see Figure S6.1).

$$E^*_{ox}(\mathbf{2a}^-) = E_{p/2} - E^{0-0} = 1.02 - 2.52 = -1.5 \text{ V}$$

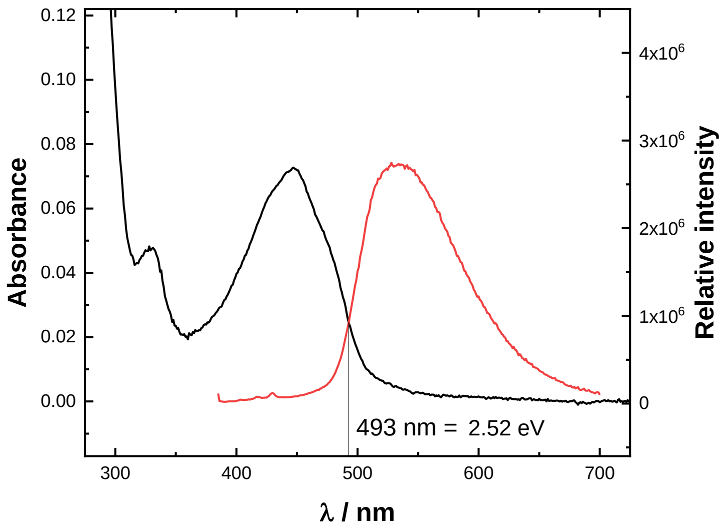


**Figure S6.1** Normalized UV-VIS and fluorescence spectra of anion **2a**$^-$.

# S7. Experimental and theoretical NMR data in the presence of base

**NMR analysis of mixture 3-methyllumichrome (1a-H) with tetrabutylammonium acetate in mixture of solvents $CD_3CN$/DMSO-$d_6$ 85/15**

Stock solution (c = 17 mM) of **1a-H** was prepared from 6.58 mg of 3-methyllumichrome (**1a-H**) and 1500 μl of DMSO-$d_6$. Stock solution (c = 34 mM) of tetrabutylammonium acetate (TBAOAc) was prepared from 30.50 mg of TBAOAc and 3000 μl of $CD_3CN$. Into NMR tube was added solution of flavine, TBAOAc and $CD_3CN$ in different ratio.

**Table S7.1** Preparation and NMR analysis of mixture **1a-H** with TBAOAc

| | **1:0** | **1:0.5** | **1:1** | **1:2** | **1.3** | **1:4** | **1:5** | **1:6** | **1:7** | **1:8** | **1.9** | **1:10** | **1:100** |
|---|---|---|---|---|---|---|---|---|---|---|---|---|---|
| **V(1a-H)** | 100 | 100 | 100 | 100 | 100 | 100 | 100 | 100 | 100 | 100 | 100 | 100 | |
| **c(1a-H)** | 2.4 | 2.4 | 2.4 | 2.4 | 2.4 | 2.4 | 2.4 | 2.4 | 2.4 | 2.4 | 2.4 | 2.4 | 2.7 |
| **V(TBAOAc)** | 0 | 25 | 50 | 100 | 150 | 200 | 250 | 300 | 350 | 400 | 450 | 500 | |
| **C(TBAOAc)** | 0 | 1.2 | 2.4 | 4.8 | 7.2 | 9.6 | 12.0 | 14.4 | 16.8 | 19.2 | 21.6 | 24 | 300 |
| **V($CD_3CN$)** | 600 | 575 | 550 | 500 | 450 | 400 | 350 | 300 | 250 | 200 | 150 | 100 | |
| **ppm** | 7.918 | 7.901 | 7.864 | 7.847 | 7.837 | 7.833 | 7.820 | 7.816 | 7.815 | 7.806 | 7.807 | 7.815 | 7.749 |
| **ppm** | 7.702 | 7.687 | 7.653 | 7.637 | 7.628 | 7.624 | 7.612 | 7.609 | 7.618 | 7.600 | 7.608 | 7.600 | 7.547 |
| **ppm** | 2.499 | 2.493 | 2.479 | 2.472 | 2.468 | 2.467 | 2.462 | 2.460 | 2.461 | 2.457 | 2.457 | 2.461 | 2.432 |
| **pm** | 2.476 | 2.470 | 2.456 | 2.452 | 2.447 | 2.446 | 2.440 | 2.438 | 2.438 | 2.435 | 2.436 | 2.437 | 2.411 |

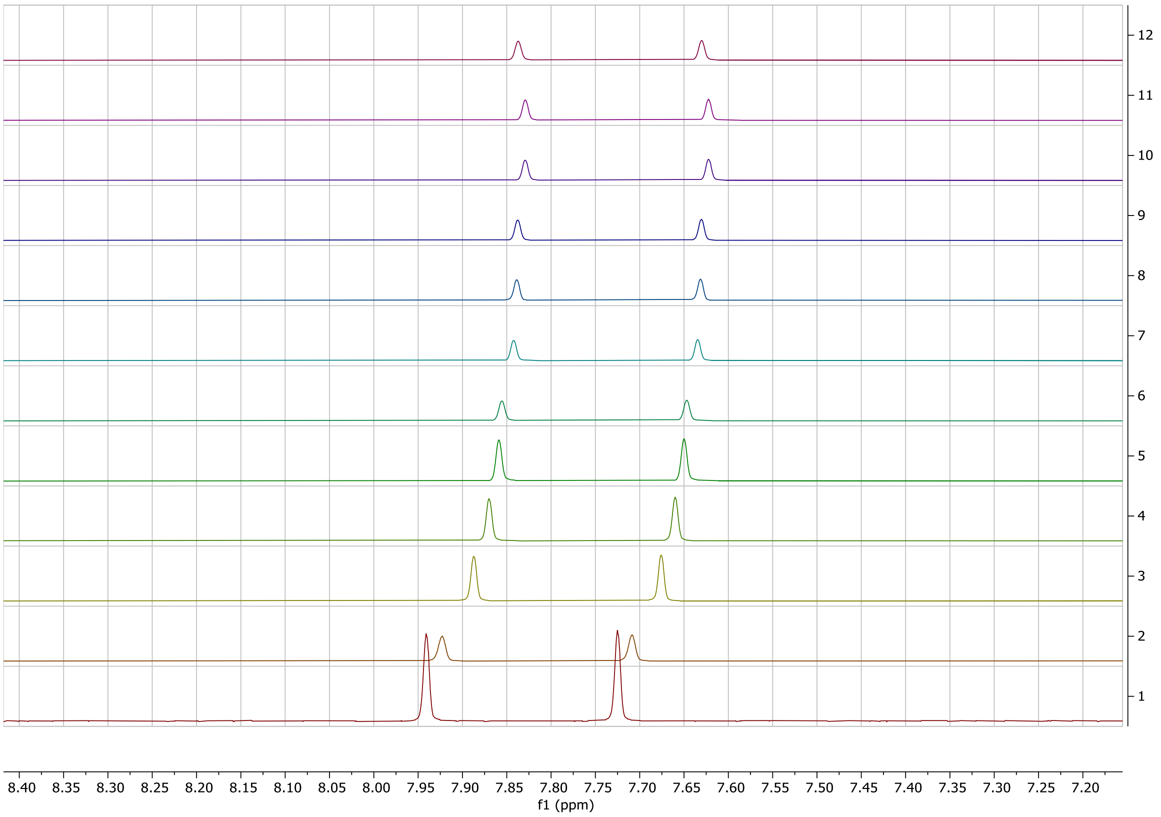


**Figure S7.1** Changes in chemical shifts of aromatic protons H6 and H9 after addition of TBAOAc

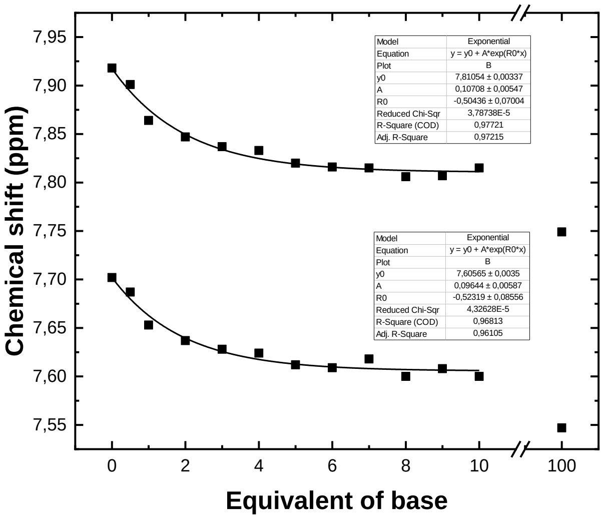


**Figure S7.2** Changes in chemical shifts of aromatic protons H6 and H9 after addition of TBAOAc

**NMR analysis of mixture 3-methyllumichrome (1a-H) with tetrabutylammonium dihydrogenphosphate in mixture of solvents $CD_3CN$/DMSO-$d_6$ 85/15.**

Stock solution (c = 17.5 mM) of **1a-H** was prepared from 6.75 mg of 3-methyllumichrome (**1a-H**) and 1500 µl of DMSO-$d_6$. Stock solution (c = 36 mM) of tetrabutylammonium dihydrogenphosphate ($TBAH_2PO_4$) was prepared from 36.70 mg of $TBAH_2PO_4$ and 3000 µl of $CD_3CN$. Into NMR tube was added solution of flavine, $TBAH_2PO_4$ and $CD_3CN$ in different ratio.

**Table S7.2** Preparation and NMR analysis of mixture **1a-H** with $TBAH_2PO_4$

| | **1:0** | **1:0.5** | **1:1** | **1:2** | **1.3** | **1:4** | **1:5** | **1:6** | **1:7** | **1:8** | **1.9** | **1:10** | **1:85** |
|---|---|---|---|---|---|---|---|---|---|---|---|---|---|
| **V(1a-H)** | 100 | 100 | 100 | 100 | 100 | 100 | 100 | 100 | 100 | 100 | 100 | 100 | |
| **c(1a-H)** | 2.5 | 2.5ř | 2.5 | 2.5 | 2.5 | 2.5 | 2.5 | 2.5 | 2.5 | 2.5 | 2.5 | 2.5 | |
| **V($TBAH_2PO_4$)** | 0 | 25 | 50 | 100 | 150 | 200 | 250 | 300 | 350 | 400 | 450 | 500 | |
| **C($TBAH_2PO_4$)** | 0 | 1.25 | 2.5 | 5.1 | 7.7 | 10.3 | 12.85 | 15.4 | 18.0 | 20.6 | 23.1 | 25.7 | 210.4 |
| **V($CD_3CN$)** | 600 | 575 | 550 | 500 | 450 | 400 | 350 | 300 | 250 | 200 | 150 | 100 | |
| **ppm** | 7.918 | 7.890 | 7.863 | 7.827 | 7.799 | 7.778 | 7.765 | 7.750 | 7.738 | 7.730 | 7.724 | 7.719 | 7.652 |
| **ppm** | 7.702 | 7.676 | 7.652 | 7.619 | 7.595 | 7.575 | 7.563 | 7.549 | 7.538 | 7.531 | 7.525 | 7.520 | 7.449 |
| **ppm** | 2.500 | 2.488 | 2.479 | 2.466 | 2.457 | 2.448 | 2.444 | 2.438 | 2.433 | 2.430 | 2.428 | 2.426 | 2.395 |
| **ppm** | 2.476 | 2.465 | 2.456 | 2.443 | 2.433 | 2.426 | 2.420 | 2.415 | 2.410 | 2.407 | 2.405 | 2.403 | 2.373 |

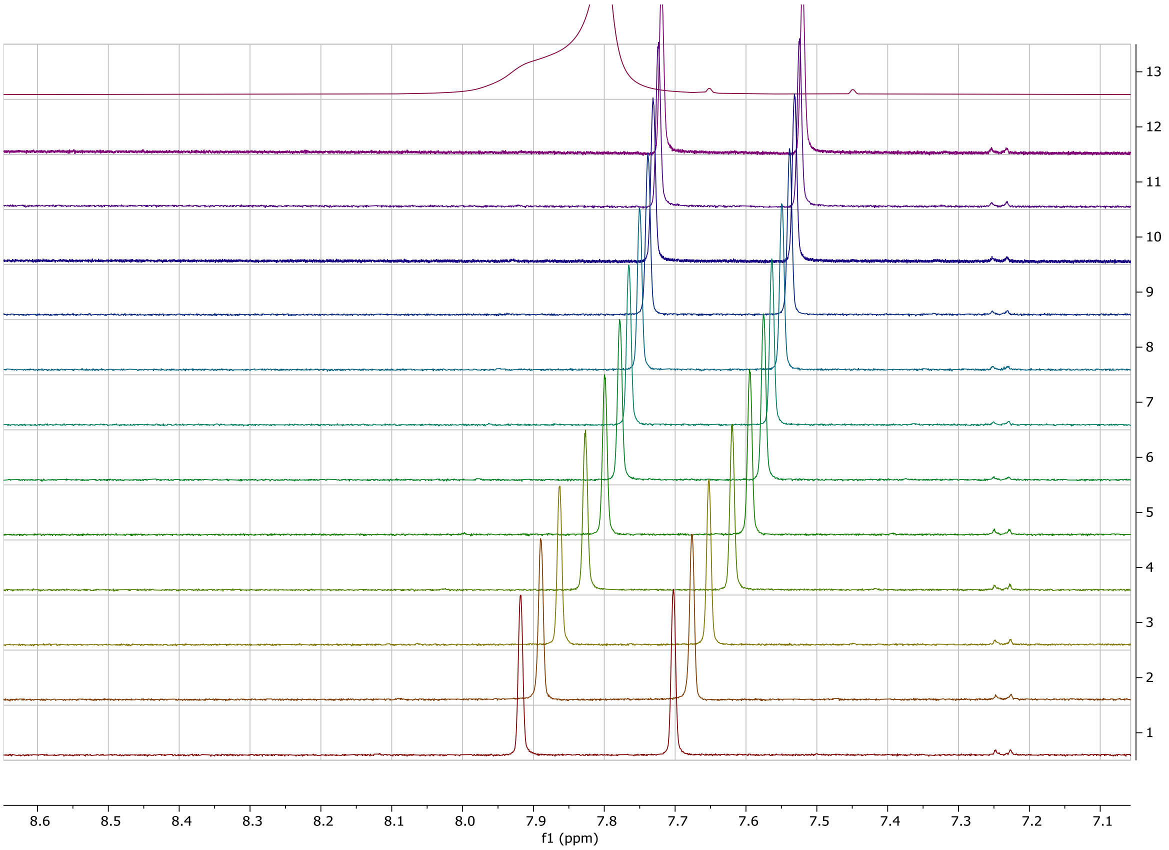


**Figure S7.3** Changes in chemical shifts of aromatic protons H6 and H9 after addition of $TBAH_2PO_4$.

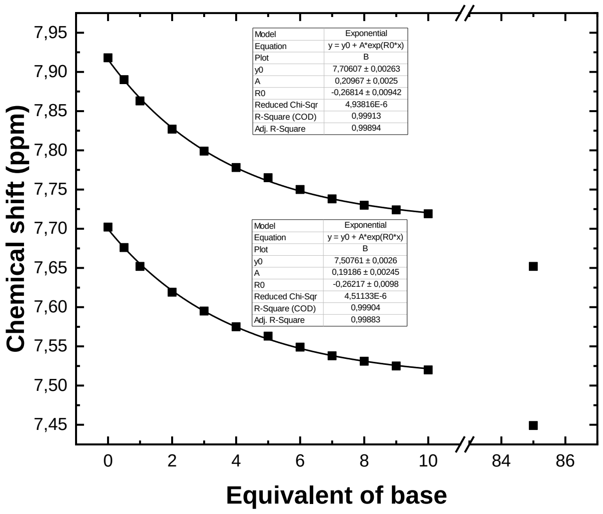


**Figure S7.4** Changes in chemical shifts of aromatic protons H6 and H9 after addition of $TBAH_2PO_4$.

**Table S7.3:** Chemical shifts of selected protons ($C^7$-$CH_3$, $C^8$-$CH_3$, $H^6$, $H^9$) of flavin derivatives in mixture of solvents $CD_3CN$/DMSO-$d_6$ 85/15 or in common solvents after addition of base (and acid).

| **Flavine derivative** | ppm | ppm | ppm | ppm |
|---|---|---|---|---|
| 3-Methyllumichrome (**1a-H**) | 2.476 | 2.500 | 7.701 | 7.917 |
| 3-Methyllumichrome (**1a-H**) + TBAOAc | 2.429 | 2.451 | 7.588 | 7.793 |
| 3-Methyllumichrome (**1a-H**) + TBAOAc + $CF_3COOD$ | 2.474 | 2.497 | 7.699 | 7.913 |
| 3-Methyllumichrome (**1a-H**) + $TBAH_2PO_4$ | 2.400 | 2.423 | 7.511 | 7.710 |
| 3-Methyllumichrome (**1a-H**) + $TBAH_2PO_4$ + $CF_3COOD$ | 2.479 | 2.502 | 7.706 | 7.920 |
| 3-Methyllumichrome (**1a-H**) + TBAOH | 2.392 | 2.415 | 7.499 | 7.703 |
| 3-Methyllumichrome (**1a-H**) + excess of TBAOAc | 2.411 | 2.432 | 7.547 | 7.749 |
| 3-Methyllumichrome (**1a-H**) + excess of $TBAH_2PO_4$ | 2.373 | 2.395 | 7.449 | 7.652 |
| 3-Methyllumichrome (**1a-H**) + TBAOAc in DMF-$D_6$ | 2.427 | 2.449 | 7.532 | 7.753 |
| 3-Methyllumichrome (**1a-H**) + TBAOAc + $CF_3COOD$ in DMF-$D_6$ | 2.515 | 2.538 | 7.740 | 7.950 |
| 3-Methyllumichrome (**1a-H**) in $CD_3CN$ | 2.502 | 2.525 | 7.714 | 7.947 |
| 3-Methyllumichrome (**1a-H**) in DMSO-$d_6$ | 2.454 | 2.478 | 7.705 | 7.930 |
| 3-Methyllumichrome (**1a-H**) in $CD_3COOD$ | 2.550 | 2.590 | 7.903 | 8.087 |
| 3-Methyllumichrome (**1a-H**) in DMF-$D_6$ | 2.517 | 2.539 | 7.742 | 7.955 |
| 1-Methyllumichrome (**3a-H**) | 2.484 | 2.514 | 7.768 | 7.929 |
| 1-Methyllumichrome (**3a-H**) + TBAOAc | 2.470 | 2.497 | 7.728 | 7.902 |
| 1-Methyllumichrome (**3a-H**) + TBAOAc + $CF_3COOD$ | 2.483 | 2.513 | 7.767 | 7.929 |
| 1-Methyllumichrome (**3a-H**) + $TBAH_2PO_4$ | 2.453 | 2.478 | 7.673 | 7.863 |
| 1-Methyllumichrome (**3a-H**) + $TBAH_2PO_4$ + $CF_3COOD$ | 2.481 | 2.511 | 7.753 | 7.917 |
| 1-Methyllumichrome (**3a-H**) + TBAOH | 2.436 | 2.457 | 7.637 | 7.844 |
| 1-Methyllumichrome (**3a-H**) + NaH | n.v. | n.v. | 7.625 | 7.856 |
| Lumiflavin (**4a-H**) | 2.413 | 2.516 | 7.642 | 7.881 |
| Lumiflavin (**4a-H**) + TBAOAc | 2.412 | 2.515 | 7.645 | 7.879 |
| Lumiflavin (**4a-H**) + TBAOAc + $CF_3COOD$ | 2.413 | 2.517 | 7.646 | 7.882 |
| Lumiflavin (**4a-H**) + $TBAH_2PO_4$ | 2.411 | 2.517 | 7.647 | 7.881 |
| Lumiflavin (**4a-H**) + TBAOH | Aduct and decomposition | | | |
| 1,3-Dimethyllumichrome (**5a**) | 2.493 | 2.522 | 7.776 | 7.944 |
| 1,3-Dimethyllumichrome (**5a**) + TBAOAc | 2.486 | 2.515 | 7.776 | 7.937 |
| 1,3-Dimethyllumichrome (**5a**) + TBAOAc + $CF_3COOD$ | 2.492 | 2.516 | 7.779 | 7.941 |
| 1,3-Dimethyllumichrome (**5a**) + $TBAH_2PO_4$ | 2.482 | 2.511 | 7.765 | 7.930 |
| 1,3-Dimethyllumichrome (**5a**)+ $TBAH_2PO_4$ + $CF_3COOD$ | 2.484 | 2.512 | 7.766 | 7.931 |
| 1,3-Dimethyllumichrome (**5a**)+ TBAOH – ADUCT! | 2.109 | 2.133 | 6.591 | 6.812 |
| Lumichrome | 2.469 | 2.495 | 7.689 | 7.901 |

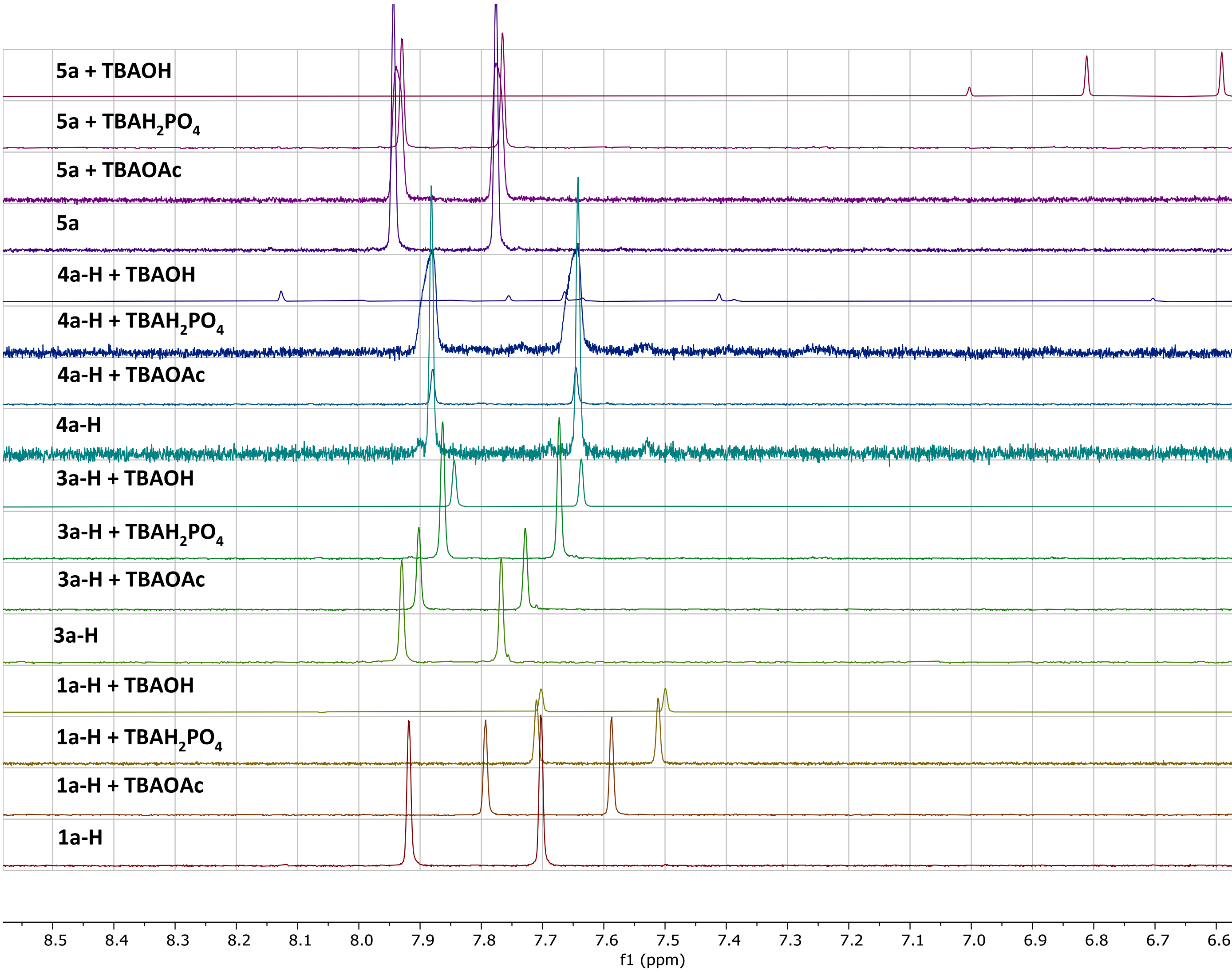


**Figure S7.5** NMR spectra of flavin and formation of their anions after addition of TBAOAc, $TBAH_2PO_4$ or TBAOH.

**Table S7.4:** Changes in chemical shift 3-methyllumichrome (1a-H) in mixture of solvents $CD_3CN$/DMSO-$d_6$ 85/15 after addition of different bases (10 eq) and $CF_3COOD$ (10 eq).

| | ppm | ppm | ppm | ppm |
|---|---|---|---|---|
| 3-Methyllumichrome **1a-H** | 2.476 | 2.499 | 7.702 | 7.918 |
| + TBAOAc | 2.429 | 2.451 | 7.588 | 7.793 |
| + TBAOAc + $CF_3COOD$ | 2.474 | 2.497 | 7.699 | 7.913 |
| + excess (100 eq) TBAOAc | 2.411 | 2.432 | 7.546 | 7.749 |
| + NaOH | 2.467 | 2.490 | 7.680 | 7.899 |
| + NaOH + $CF_3COOD$ | 2.479 | 2.502 | 7.708 | 7.926 |
| + $TBAHCO_3$ | 2.474 | 2.298 | 7.700 | 7.912 |
| + $TBAHCO_3$ + $CF_3COOD$ | 2.474 | 2.499 | 7.702 | 7.914 |
| + DBU | 2.392 | 2.413 | 7.473 | 7.693 |
| + DBU + $CF_3COOD$ | 2.477 | 2.500 | 7.703 | 7.916 |
| + $K_2CO_3$ | 2.455 | 2.479 | 7.655 | 7.867 |
| + $K_2CO_3$ + $CF_3COOD$ | 2.475 | 2.499 | 7.703 | 7.919 |
| + NaH | 2.387 | 2.408 | 7.484 | 7.710 |
| + NaH + $CF_3COOD$ | 2.478 | 2.508 | 7.710 | 7.941 |
| + $TBAH_2PO_4$ | 2.400 | 2.423 | 7.511 | 7.710 |
| + $TBAH_2PO_4$ + $CF_3COOD$ | 2.479 | 2.502 | 7.706 | 7.920 |
| +excess (85 eq) $TBAH_2PO_4$ | 2.373 | 2.395 | 7.449 | 7.652 |
| + TBAOH | 2.392 | 2.415 | 7.499 | 7.703 |

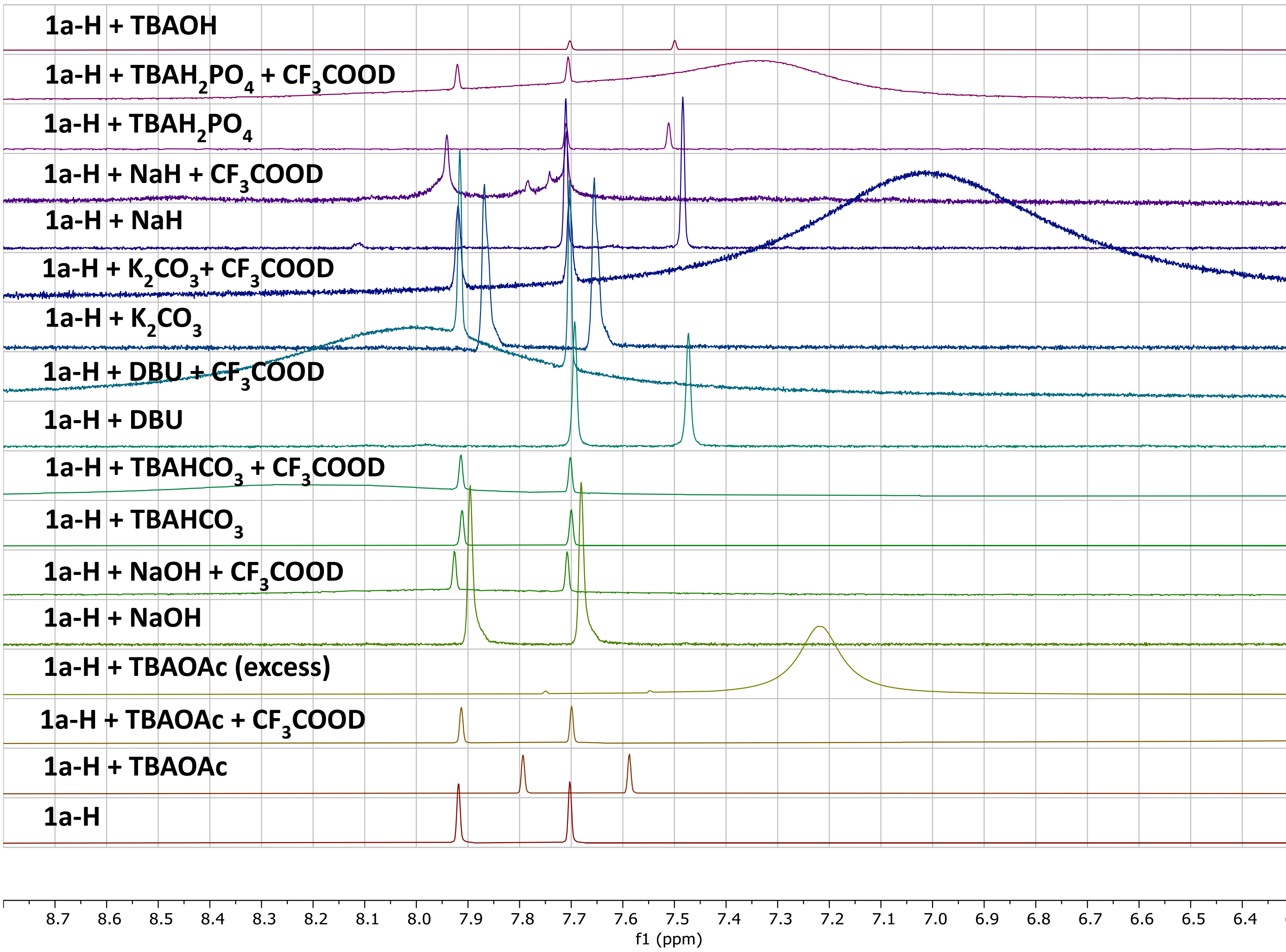


**Figure S7.6** Changes in chemical shifts of aromatic protons H6 and H6 of 3-methyllumichrome (**1a-H**) after addition of different bases and $CF_3COOD$.

**Table S7.5** Theoretical NMR chemical shifts calculated using GIAO/B3LYP/cc-pVTZ (with solvent - acetonitrile)

| | **1a-H** | | **1a⁻** | | **2a-H** | | **3a-H** | | **3a⁻** | | **4a-H** | | **4a⁻** | |
|---|---|---|---|---|---|---|---|---|---|---|---|---|---|---|
| | isotropic (shielding constant) | **sigma (chemical shift)** | isotropic (shielding constant) | **sigma (chemical shift)** | isotropic (shielding constant) | **sigma (chemical shift)** | isotropic (shielding constant) | **sigma (chemical shift)** | isotropic (shielding constant) | **sigma (chemical shift)** | isotropic (shielding constant) | **sigma (chemical shift)** | isotropic (shielding constant) | **sigma (chemical shift)** |
| TMS | 31.7369 | | | | | | | | | | | | | |
| $H^6$ | 23.4623 | **8.2746** | 23.793 | **7.9439** | 23.5404 | **8.1965** | 23.4604 | **8.2765** | 23.6383 | **8.0986** | 23.5485 | **8.1884** | 23.7998 | **7.9371** |
| $H^7$ | 23.6817 | **8.0552** | 24.0339 | **7.703** | 24.1789 | **7.558** | 23.5878 | **8.1491** | 23.8006 | **7.9363** | 23.9368 | **7.8001** | 24.2554 | **7.4815** |
| $N^1$-$CH_3$ | | | | | | | 29.0408 | **2.6961** | 29.123 | **2.6139** | | | | |
| $N^1$-$CH_3$ | | | | | | | 29.0954 | **2.6415** | 29.2074 | **2.5295** | | | | |
| $N^1$-$CH_3$ | | | | | | | 29.0407 | **2.6962** | 29.1232 | **2.6137** | | | | |
| $N^3$-$CH_3$ | 27.3091 | **4.4278** | 27.1245 | **4.6124** | 27.2646 | **4.4723** | | | | | | | | |
| $N^3$-$CH_3$ | 28.6593 | **3.0776** | 28.8716 | **2.8653** | 28.7607 | **2.9762** | | | | | | | | |
| $N^3$-$CH_3$ | 28.6611 | **3.0758** | 28.8715 | **2.8654** | 28.7626 | **2.9743** | | | | | | | | |
| $N^{10}$-$CH_3$ | | | | | | | | | | | 29.0444 | **2.6925** | 29.1599 | **2.577** |
| $N^{10}$-$CH_3$ | | | | | | | | | | | 29.1153 | **2.6216** | 29.2597 | **2.4772** |
| $N^{10}$-$CH_3$ | | | | | | | | | | | 29.0446 | **2.6923** | 29.1599 | **2.577** |
| $C^8$-$CH_3$ | 29.0544 | **2.6825** | 29.1682 | **2.5687** | 29.0981 | **2.6388** | 29.0878 | **2.6491** | 29.1535 | **2.5834** | 29.1832 | **2.5537** | 29.2647 | **2.4722** |
| $C^8$-$CH_3$ | 29.1154 | **2.6215** | 29.2699 | **2.467** | 29.1969 | **2.54** | 29.0878 | **2.6491** | 29.1537 | **2.5832** | 29.1834 | **2.5535** | 29.2648 | **2.4721** |
| $C^8$-$CH_3$ | 29.0548 | **2.6821** | 29.1684 | **2.5685** | 29.098 | **2.6389** | 29.1164 | **2.6205** | 29.2131 | **2.5238** | 29.2073 | **2.5296** | 29.33 | **2.4069** |
| $C^7$-$CH_3$ | 29.0945 | **2.6424** | 29.1982 | **2.5387** | 29.1897 | **2.5472** | 28.4337 | **3.3032** | 28.7001 | **3.0368** | 28.3682 | **3.3687** | 28.7133 | **3.0236** |
| $C^7$-$CH_3$ | 29.095 | **2.6419** | 29.1982 | **2.5387** | 29.1898 | **2.5471** | 28.4338 | **3.3031** | 28.6993 | **3.0376** | 28.3692 | **3.3677** | 28.7139 | **3.023** |
| $C^7$-$CH_3$ | 29.1263 | **2.6106** | 29.284 | **2.4529** | 29.2129 | **2.524** | 27.1606 | **4.5763** | 26.9696 | **4.7673** | 25.9269 | **5.81** | 26.0117 | **5.7252** |
| $N^1$-H | 23.8598 | **7.8771** | | | | | | | | | | | | |
| $N^3$-H | | | | | | | 23.8013 | **7.9356** | | | 23.9056 | **7.8313** | | |
| $N^{10}$-H | | | | | 22.6767 | **9.0602** | | | | | | | | |

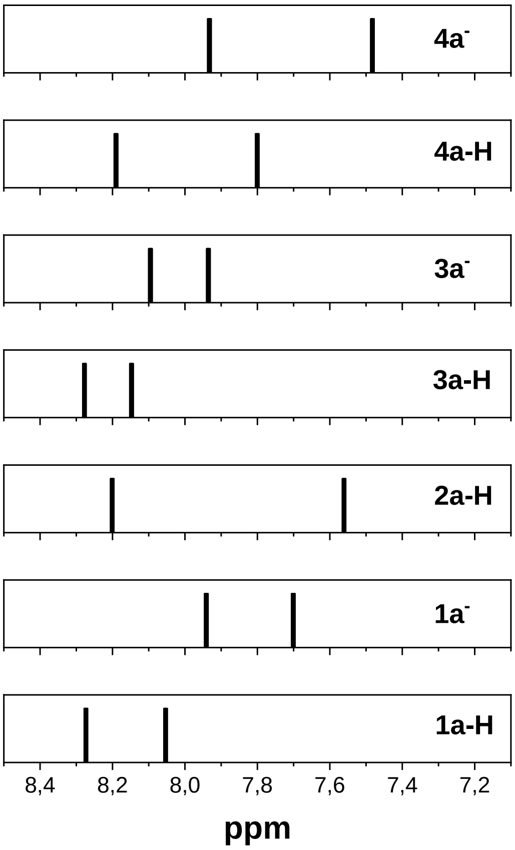


**Figure S7.7** Visualization of theoretical chemical shift of aromatic protons $H^6$ and $H^9$ GIAO/B3LYP/cc-pVTZ (with solvent - acetonitrile)

**Table S7.6** Theoretical NMR chemical shift calculated using GIAO/B3LYP/aug-CC-pVZT (without solvent – acetonitrile)

| | **1a-H** | | **$1a^-$** | | **2a-H** | | **3a-H** | | **$3a^-$** | | **4a-H** | | **$4a^-$** | |
|---|---|---|---|---|---|---|---|---|---|---|---|---|---|---|
| | isotropic (shielding constant) | **sigma (chemical shift)** | isotropic (shielding constant) | **sigma (chemical shift)** | isotropic (shielding constant) | **sigma (chemical shift)** | isotropic (shielding constant) | **sigma (chemical shift)** | isotropic (shielding constant) | **sigma (chemical shift)** | isotropic (shielding constant) | **sigma (chemical shift)** | isotropic (shielding constant) | **sigma (chemical shift)** |
| TMS | 31.7369 | | | | | | | | | | | | | |
| $H^6$ | 23.4371 | **8.2998** | 24.0536 | **7.6833** | 23.59 | **8.1469** | 23.4334 | **8.3035** | 23.8034 | **7.9335** | 23.5857 | **8.1512** | 24.0843 | **7.6526** |
| $H^9$ | 23.7968 | **7.9401** | 24.2957 | **7.4412** | 24.7464 | **6.9905** | 23.7168 | **8.0201** | 24.1716 | **7.5653** | 24.4386 | **7.2983** | 25.116 | **6.6209** |
| $N^1$-$CH_3$ | | | | | | | 28.5709 | **3.166** | 29.0462 | **2.6907** | | | | |
| $N^1$-$CH_3$ | | | | | | | 28.5708 | **3.1661** | 29.0467 | **2.6902** | | | | |
| $N^1$-$CH_3$ | | | | | | | 27.0164 | **4.7205** | 26.6123 | **5.1246** | | | | |
| $N^3$-$CH_3$ | 27.2621 | **4.4748** | 26.9345 | **4.8024** | 27.1797 | **4.5572** | | | | | | | | |
| $N^3$-$CH_3$ | 28.7171 | **3.0198** | 29.1058 | **2.6311** | 28.8384 | **2.8985** | | | | | | | | |
| $N^3$-$CH_3$ | 28.7191 | **3.0178** | 29.1077 | **2.6292** | 28.8392 | **2.8977** | | | | | | | | |
| $N^{10}$-$CH_3$ | | | | | | | | | | | 28.8326 | **2.9043** | 29.5071 | **2.2298** |
| $N^{10}$-$CH_3$ | | | | | | | | | | | 28.8334 | **2.9035** | 29.5072 | **2.2297** |
| $N^{10}$-$CH_3$ | | | | | | | | | | | 25.57 | **6.1669** | 25.4437 | **6.2932** |
| $C^8$-$CH_3$ | 29.1614 | **2.5755** | 29.4097 | **2.3272** | 29.2256 | **2.5113** | 29.1467 | **2.5902** | 29.3478 | **2.3891** | 29.1686 | **2.5683** | 29.4166 | **2.3203** |
| $C^8$-$CH_3$ | 29.2225 | **2.5144** | 29.4933 | **2.2436** | 29.4334 | **2.3035** | 29.2075 | **2.5294** | 29.4624 | **2.2745** | 29.3495 | **2.3874** | 29.6384 | **2.0985** |
| $C^8$-$CH_3$ | 29.1618 | **2.5751** | 29.4101 | **2.3268** | 29.2256 | **2.5113** | 29.1467 | **2.5902** | 29.3478 | **2.3891** | 29.1685 | **2.5684** | 29.4168 | **2.3201** |
| $C^{7-}$ $CH_3$ | 29.2011 | **2.5358** | 29.3936 | **2.3433** | 29.3293 | **2.4076** | 29.1948 | **2.5421** | 29.3685 | **2.3684** | 29.3174 | **2.4195** | 29.5183 | **2.2186** |
| $C^7$ -$CH_3$ | 29.2015 | **2.5354** | 29.394 | **2.3429** | 29.3296 | **2.4073** | 29.1949 | **2.542** | 29.3685 | **2.3684** | 29.3181 | **2.4188** | 29.5177 | **2.2192** |
| $C^7$ -$CH_3$ | 29.1803 | **2.5566** | 29.4957 | **2.2412** | 29.2963 | **2.4406** | 29.1697 | **2.5672** | 29.391 | **2.3459** | 29.2855 | **2.4514** | 29.5399 | **2.197** |
| $N^1$-H | 24.3606 | **7.3763** | | | | | | | | | | | | |
| $N^3$-H | | | | | | | 24.2241 | **7.5128** | | | 24.2745 | **7.4624** | | |
| $N^{10}$-H | | | | | 23.7653 | **7.9716** | | | | | | | | |

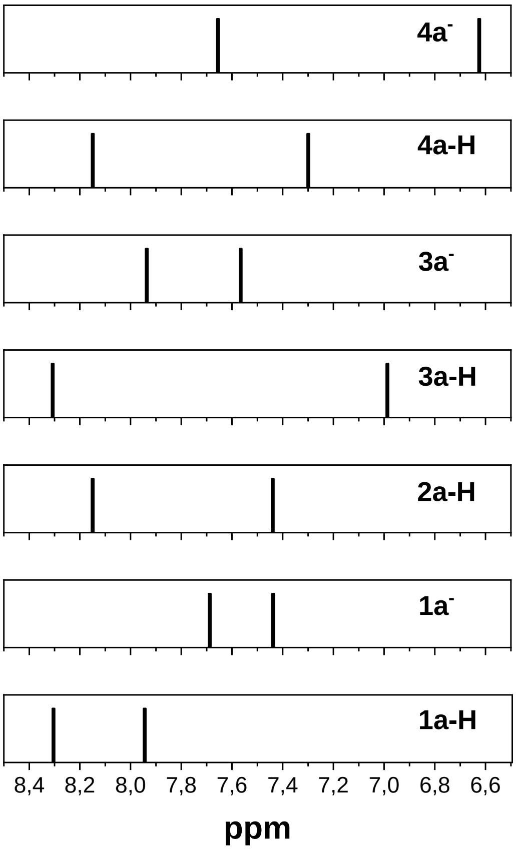


**Figure S7.8** Visualization of theoretical chemical shift of aromatic protons $H^6$ and $H^9$ GIAO/B3LYP/aug-cc-pVTZ (without solvent - acetonitrile)

**Table S7.7** Theoretical NMR chemical shift calculated using GIAO/B3LYP/aug-cc-pVZT (with solvent - acetonitrile)

| | **1a-H** | | **1a$^-$** | | **2a-H** | | **3a-H** | | **3a$^-$** | | **4a-H** | | **4a$^-$** | |
|---|---|---|---|---|---|---|---|---|---|---|---|---|---|---|
| | isotropic (shielding constant) | **sigma (chemical shift)** | isotropic (shielding constant) | **sigma (chemical shift)** | isotropic (shielding constant) | **sigma (chemical shift)** | isotropic (shielding constant) | **sigma (chemical shift)** | isotropic (shielding constant) | **sigma (chemical shift)** | isotropic (shielding constant) | **sigma (chemical shift)** | isotropic (shielding constant) | **sigma (chemical shift)** |
| TMS | 31.7315 | | | | | | | | | | | | | |
| $H^6$ | 23.3959 | **8.3356** | 23.6896 | **8.0419** | 23.4571 | **8.2744** | 23.3995 | **8.332** | 23.5497 | **8.1818** | 23.4573 | **8.2742** | 23.6641 | **8.0674** |
| $H^9$ | 23.6155 | **8.116** | 23.9384 | **7.7931** | 24.1025 | **7.629** | 23.4949 | **8.2366** | 23.6736 | **8.0579** | 23.8444 | **7.8871** | 24.1178 | **7.6137** |
| $N^1$-$CH_3$ | | | | | | | 28.351 | **3.3805** | 28.5503 | **3.1812** | | | | |
| $N^1$-$CH_3$ | | | | | | | 28.351 | **3.3805** | 28.5505 | **3.181** | | | | |
| $N^1$-$CH_3$ | | | | | | | 27.0748 | **4.6567** | 26.874 | **4.8575** | | | | |
| $N^3$-$CH_3$ | 27.2166 | **4.5149** | 27.01 | **4.7215** | 27.1659 | **4.5656** | | | | | | | | |
| $N^3$-$CH_3$ | 28.5926 | **3.1389** | 28.7536 | **2.9779** | 28.6844 | **3.0471** | | | | | | | | |
| $N^3$-$CH_3$ | 28.5916 | **3.1399** | 28.7524 | **2.9791** | 28.6792 | **3.0523** | | | | | | | | |
| $N^{10}$-$CH_3$ | | | | | | | | | | | 28.2786 | **3.4529** | 28.5722 | **3.1593** |
| $N^{10}$-$CH_3$ | | | | | | | | | | | 28.2789 | **3.4526** | 28.5733 | **3.1582** |
| $N^{10}$-$CH_3$ | | | | | | | | | | | 25.8366 | **5.8949** | 25.903 | **5.8285** |
| $C^8$-$CH_3$ | 29.0236 | **2.7079** | 29.1282 | **2.6033** | 29.0612 | **2.6703** | 29.0062 | **2.7253** | 29.0798 | **2.6517** | 29.003 | **2.7285** | 29.1052 | **2.6263** |
| $C^8$-$CH_3$ | 29.0752 | **2.6563** | 29.2196 | **2.5119** | 29.147 | **2.5845** | 29.0473 | **2.6842** | 29.1466 | **2.5849** | 29.0665 | **2.665** | 29.1931 | **2.5384** |
| $C^8$-$CH_3$ | 29.0233 | **2.7082** | 29.1288 | **2.6027** | 29.061 | **2.6705** | 29.0061 | **2.7254** | 29.0797 | **2.6518** | 29.0031 | **2.7284** | 29.1052 | **2.6263** |
| $C^{7-}$ $CH_3$ | 29.0669 | **2.6646** | 29.1607 | **2.5708** | 29.1568 | **2.5747** | 29.0556 | **2.6759** | 29.1142 | **2.6173** | 29.1484 | **2.5831** | 29.2177 | **2.5138** |
| $C^7$ -$CH_3$ | 29.0667 | **2.6648** | 29.1609 | **2.5706** | 29.1566 | **2.5749** | 29.0556 | **2.6759** | 29.1141 | **2.6174** | 29.1483 | **2.5832** | 29.218 | **2.5135** |
| $C^7$ -$CH_3$ | 29.0893 | **2.6422** | 29.2314 | **2.5001** | 29.1678 | **2.5637** | 29.0785 | **2.653** | 29.1617 | **2.5698** | 29.1594 | **2.5721** | 29.2647 | **2.4668** |
| $N^1$-H | 23.5899 | **8.1416** | | | | | | | | | | | | |
| $N^3$-H | | | | | | | 23.5247 | **8.2068** | | | 23.5591 | **8.1724** | | |
| $N^{10}$-H | | | | | 22.4269 | **9.3046** | | | | | | | | |

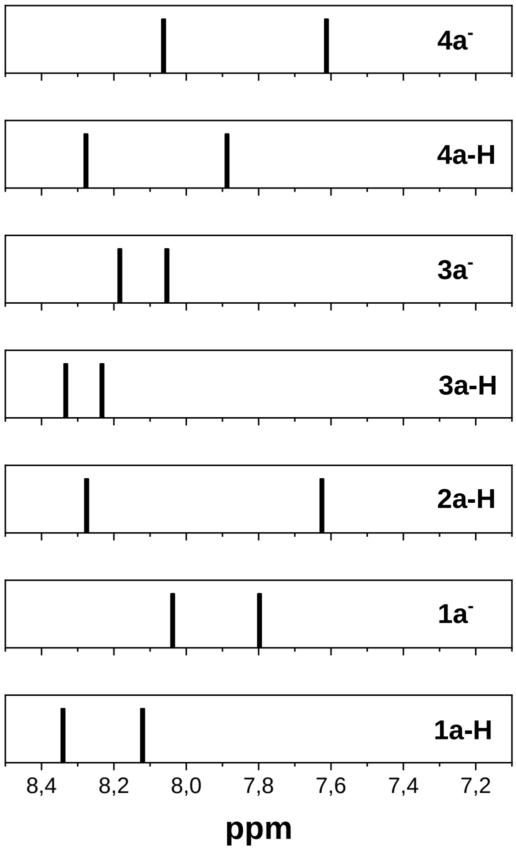


**Figure S7.9** Visualization of theoretical chemical shift of aromatic protons $H^6$ and $H^9$ GIAO/B3LYP/aug-cc-pVTZ (with solvent - acetonitrile)

# S8. Photoredox catalysis – analysis of reaction mixtures

Analysis were performed by GC-MS analysis. The results are ordered according to entries in Table 1 and Scheme 1.

### Entry 1

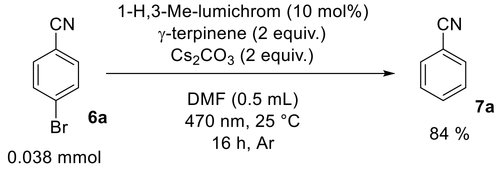


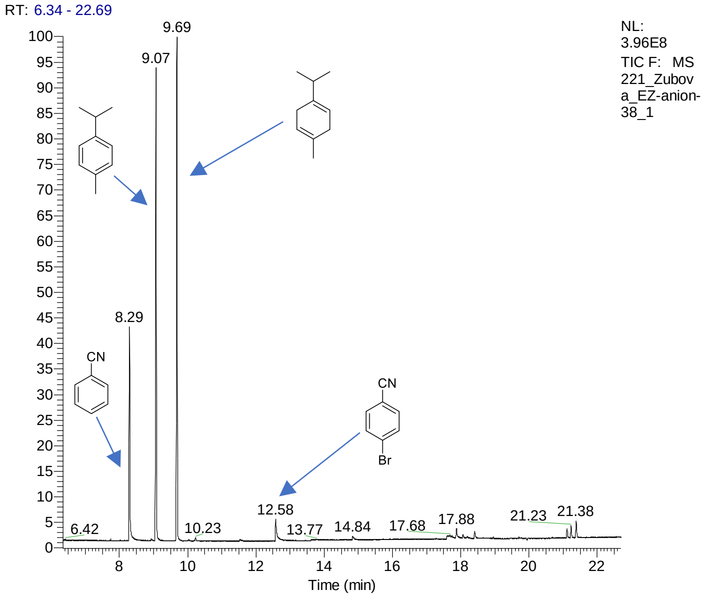


| Apex RT | % Area | | MW Da |
|---|---|---|---|
| 8.29 | 84.37 | $C_7H_5N$ | 103 |
| 12.58 | 15.63 | $C_7H_4BrN$ | 181 |

## Entry 2

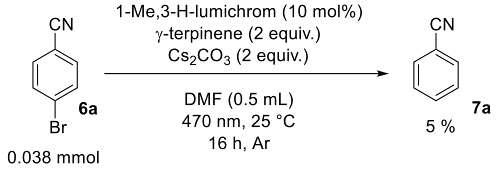


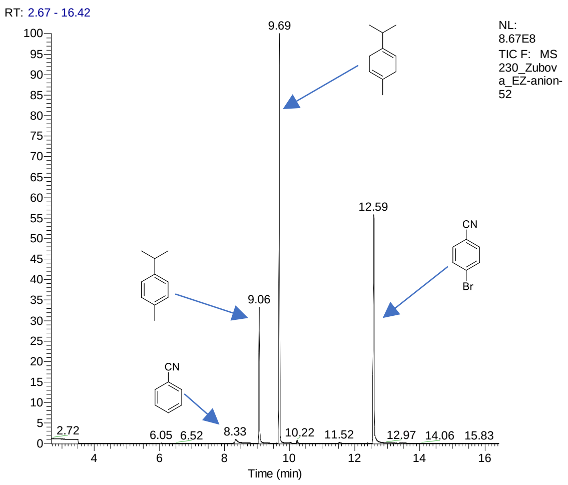


| Apex RT | % Area | | MW Da |
|---|---|---|---|
| 8.33 | 5.1 | $C_7H_5N$ | 103 |
| 12.59 | 94.9 | $C_7H_4BrN$ | 181 |

## Entry 3

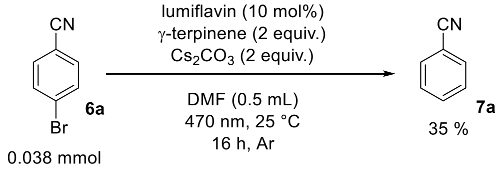


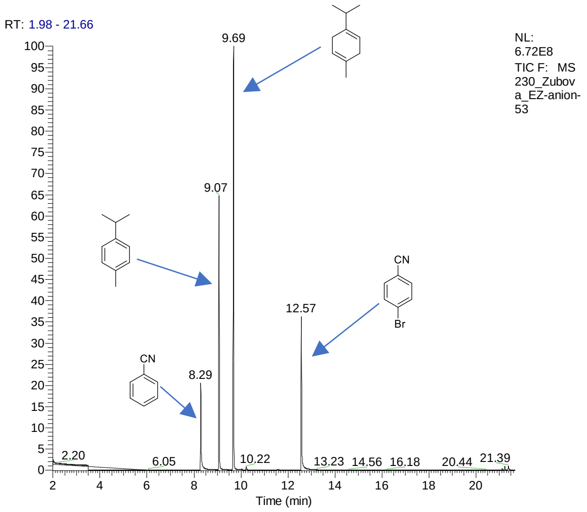


| Apex RT | % Area | | MW Da |
|---|---|---|---|
| 8.29 | 34.69 | $C_7H_5N$ | 103 |
| 12.57 | 65.31 | $C_7H_4BrN$ | 181 |

## Entry 4

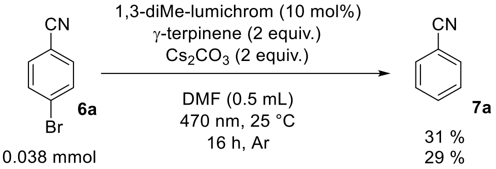


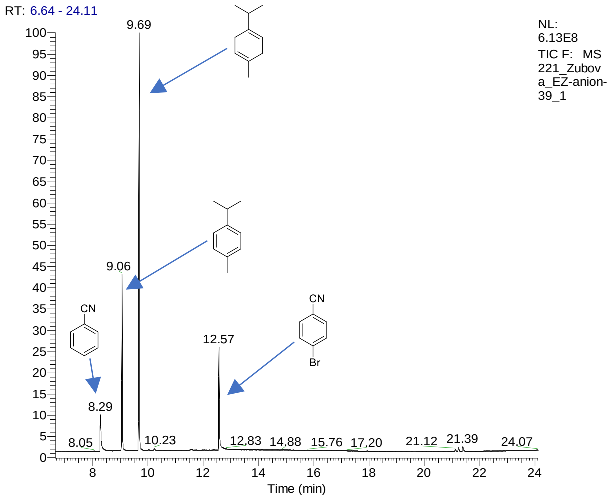


| Apex RT | % Area | | MW Da |
|---|---|---|---|
| 8.29 | 30.63 | $C_7H_5N$ | 103 |
| 12.57 | 69.37 | $C_7H_4BrN$ | 181 |

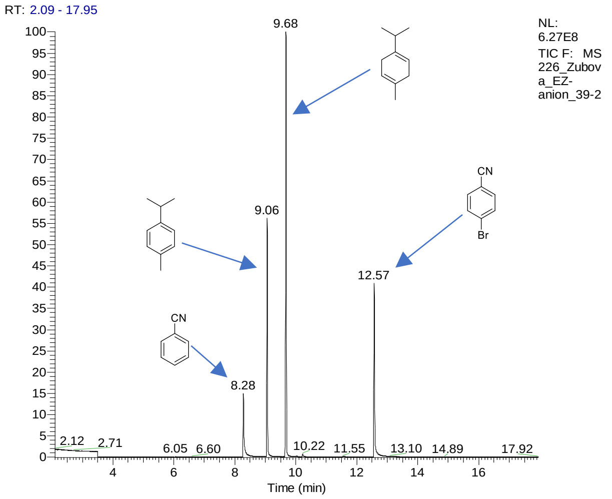


| Apex RT | % Area | | MW Da |
|---|---|---|---|
| 8.28 | 29.26 | $C_7H_5N$ | 103 |
| 12.57 | 70.74 | $C_7H_4BrN$ | 181 |

## Entry 5

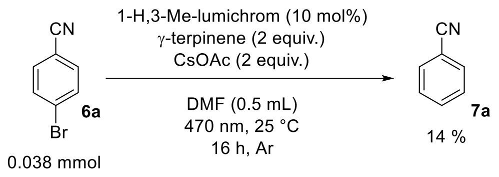


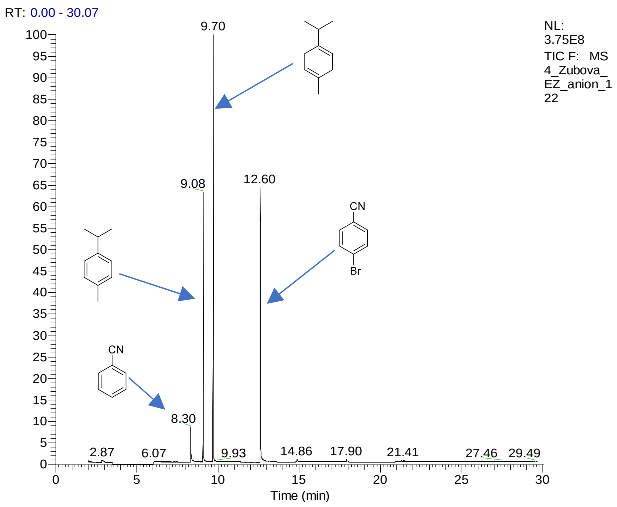


| Apex RT | % Area | | MW Da |
|---|---|---|---|
| 8.30 | 14.28 | $C_7H_5N$ | 103 |
| 12.60 | 85.72 | $C_7H_4BrN$ | 181 |

## Entry 6

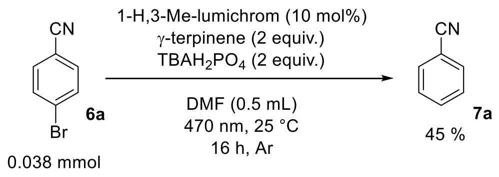


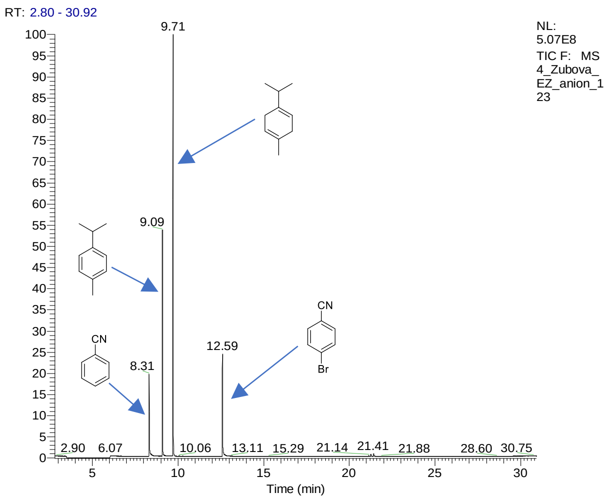


| Apex RT | % Area | | MW Da |
|---|---|---|---|
| 8.31 | 45.23 | $C_7H_5N$ | 103 |
| 12.59 | 54.77 | $C_7H_4BrN$ | 181 |

## Entry 7

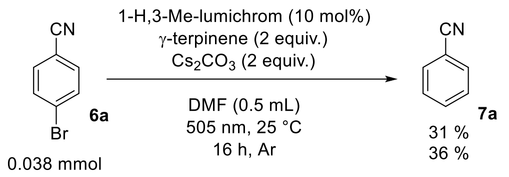


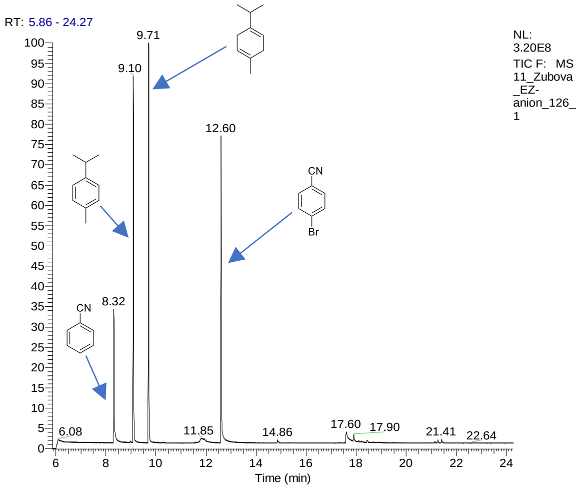


| Apex RT | % Area | | MW Da |
|---|---|---|---|
| 8.32 | 31.22 | $C_7H_5N$ | 103 |
| 12.6 | 68.78 | $C_7H_4BrN$ | 181 |

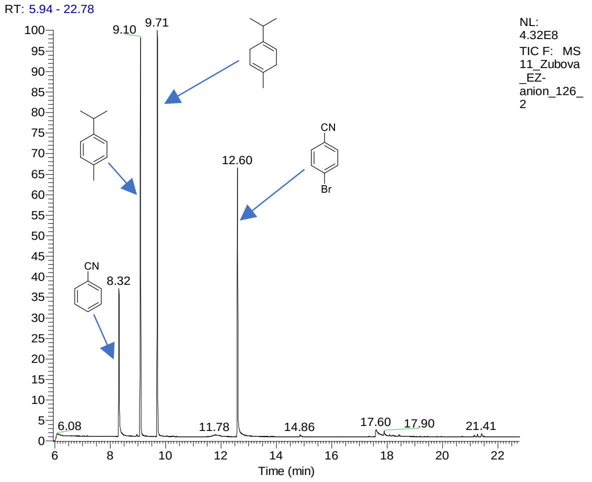


| Apex RT | % Area | | MW Da |
|---|---|---|---|
| 8.32 | 36.27 | $C_7H_5N$ | 103 |
| 12.6 | 63.73 | $C_7H_4BrN$ | 181 |

## Entry 8

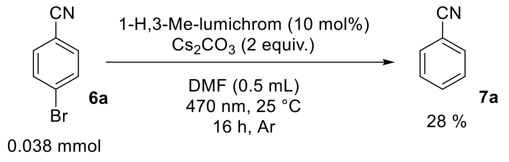


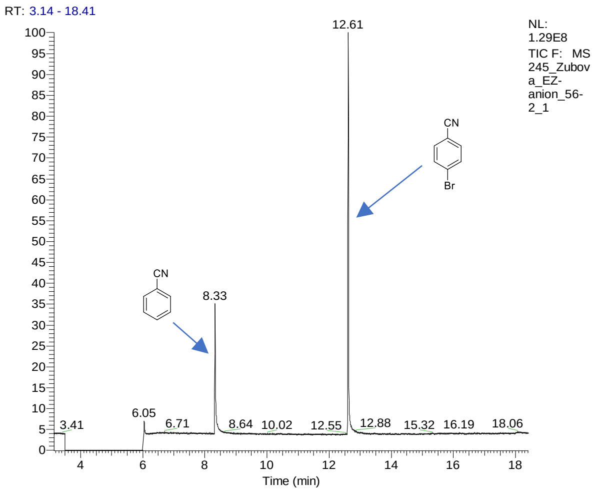


| Apex RT | % Area | | MW Da |
|---|---|---|---|
| 8.33 | 28.32 | $C_7H_5N$ | 103 |
| 12.61 | 71.68 | $C_7H_4BrN$ | 181 |

## Entry 9

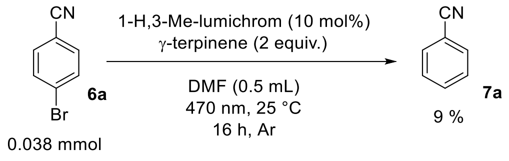


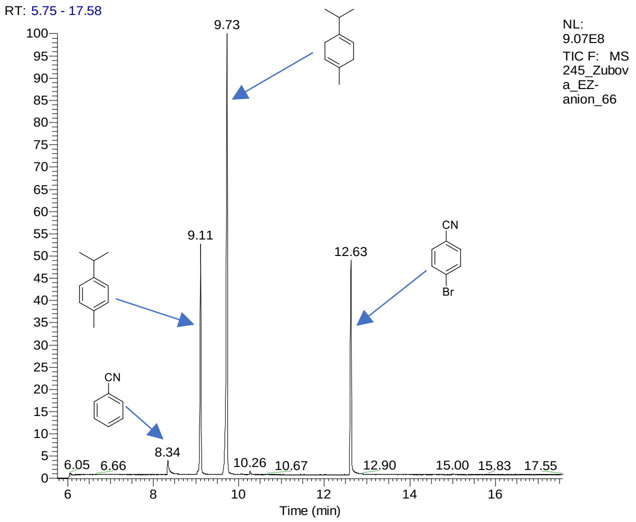


| Apex RT | % Area | | MW Da |
|---|---|---|---|
| 8.34 | 9.15 | $C_7H_5N$ | 103 |
| 12.63 | 90.85 | $C_7H_4BrN$ | 181 |

## Entry 10

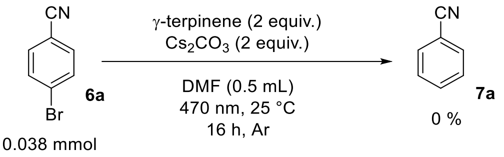


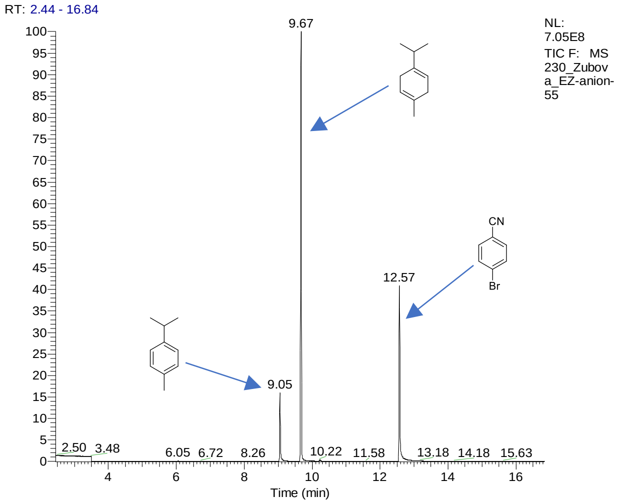


| Apex RT | % Area | | MW Da |
|---|---|---|---|
| 8.29 | not found | $C_7H_5N$ | 103 |
| 12.57 | 100 | $C_7H_4BrN$ | 181 |

## Entry 11

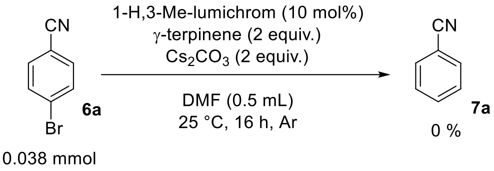


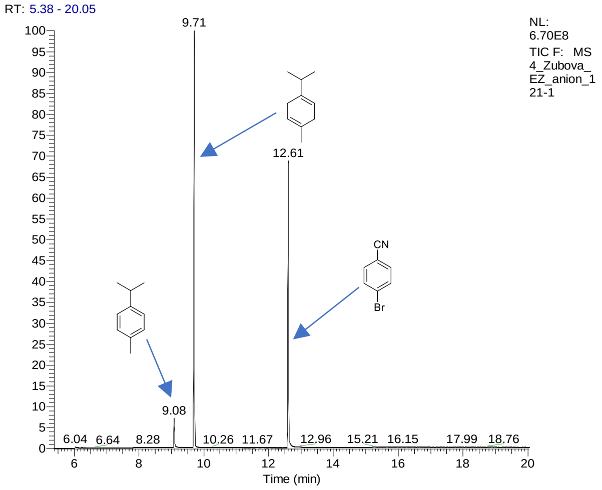


| Apex RT | % Area | | MW Da |
|---|---|---|---|
| 8.31 | not found | $C_7H_5N$ | 103 |
| 12.61 | 100 | $C_7H_4BrN$ | 181 |

## Entry 12

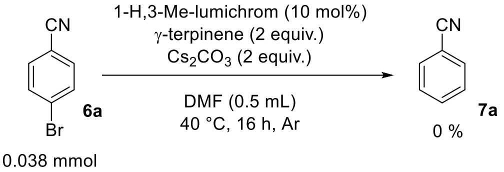


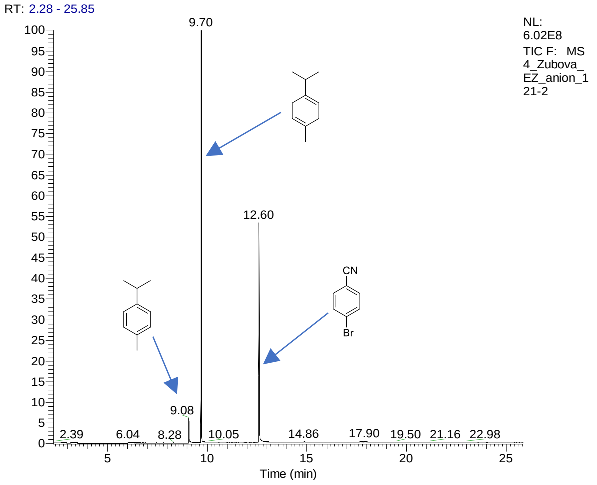


| Apex RT | % Area | | MW Da |
|---|---|---|---|
| 8.31 | not found | $C_7H_5N$ | 103 |
| 12.6 | 100 | $C_7H_4BrN$ | 181 |

## Entry 13

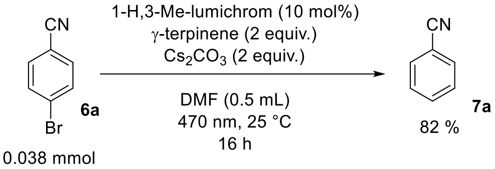


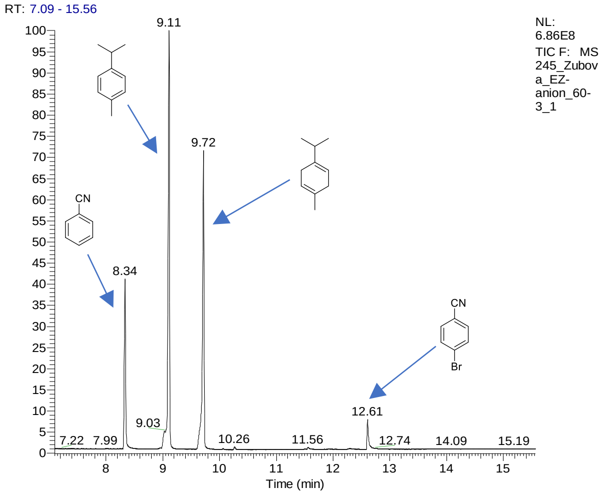


| Apex RT | % Area | | MW Da |
|---|---|---|---|
| 8.34 | 82.41 | $C_7H_5N$ | 103 |
| 12.61 | 17.59 | $C_7H_4BrN$ | 181 |

## Entry 14

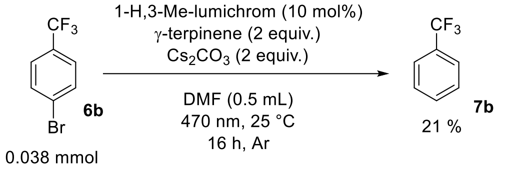


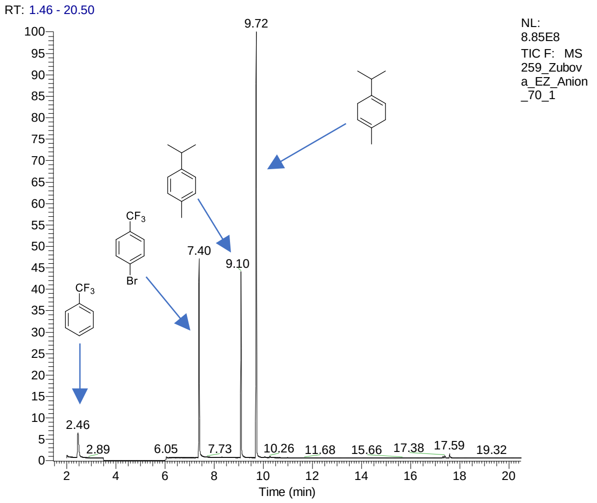


| Apex RT | % Area | | MW Da |
|---|---|---|---|
| 2.46 | 21.05 | $C_7H_5F3$ | 146 |
| 7.4 | 78.95 | $C_7H_4BrF3$ | 224 |

## Entry 15

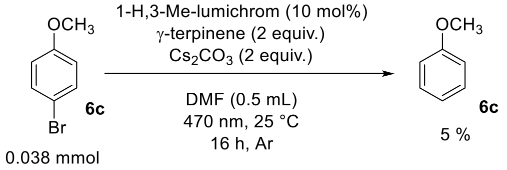


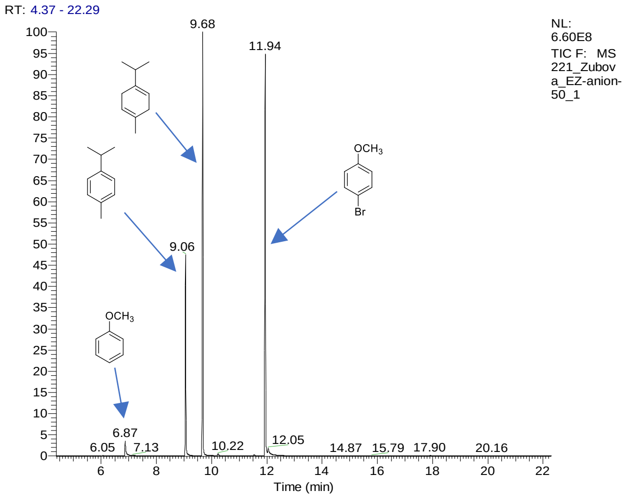


| Apex RT | % Area | | MW Da |
|---|---|---|---|
| 6.87 | 4.63 | $C_7H_8O$ | 108 |
| 11.94 | 95.37 | $C_7H_7BrO$ | 186 |

## Entry 16

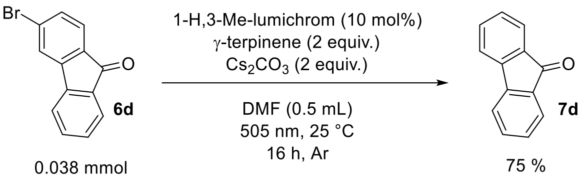


**Table S8.1** Calibration of mass spectroscopy analysis of 3-bromo-9*H*-fluoren-9-one debromination

| **7d**:**6d** | **7d** | **6d** | **6d** | **6d** | **6d** and **7d** | **7d**/(**6d** and **7d**) | **7d** |
|---|---|---|---|---|---|---|---|
| | m/z 180.06 | m/z 255.9714 | m/z 259.9697 | sum | sum | ratio | % |
| 1:0 | 2200000 | 16000 | 16000 | 32000 | 2232000 | 0.985663 | 100 |
| 1:1 | 1400000 | 835000 | 840000 | 1675000 | 3075000 | 0.455285 | 50 |
| 10:1 | 2200000 | 196000 | 200000 | 396000 | 2596000 | 0.847458 | 90.9 |
| 1:10 | 242000 | 724000 | 730000 | 1454000 | 1696000 | 0.142689 | 9.1 |
| 0:1 | 96000 | 820000 | 800000 | 1620000 | 1716000 | 0.055944 | 0 |
| | | | | | | | |
| EZ133-blank | 13600000 | 47000000 | 47000000 | 94000000 | 107600000 | 0.126394 | 9.115525 |
| EZ133-1 | 15600000 | 2600000 | 2500000 | 5100000 | 20700000 | 0.753623 | 78.2361 |

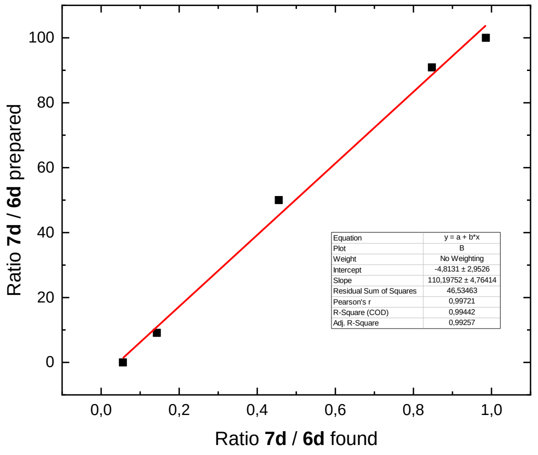


y = 110.1975x – 4.8131

**Figure S8.1** Calibration of mass spectroscopy analysis of 3-bromo-9*H*-fluoren-9-one debromination

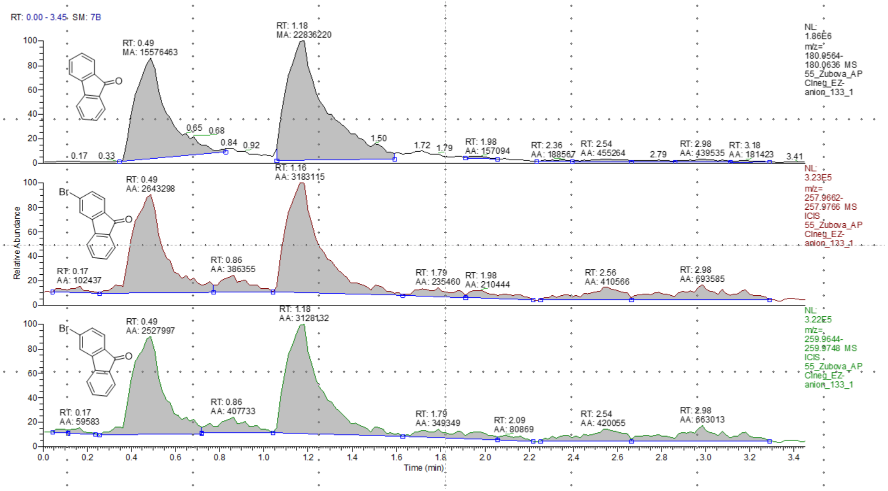


**Figure S8.2** 3-Bromo-9*H*-fluoren-9-one debromination analysis

## **Data on phosphonylations** (related to Scheme 1)

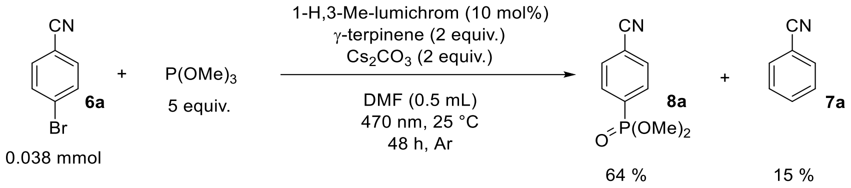


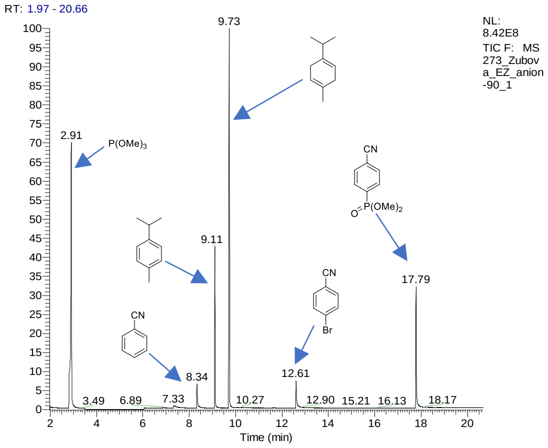


| Apex RT | % Area | | MW Da |
|---|---|---|---|
| 8.34 | 15.36 | $C_7H_5N$ | 103 |
| 12.61 | 20.67 | $C_7H_4BrN$ | 181 |
| 17.79 | 63.97 | $C_9H_{10}NO_3P$ | 211 |

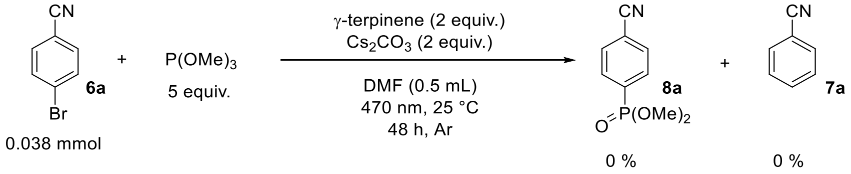


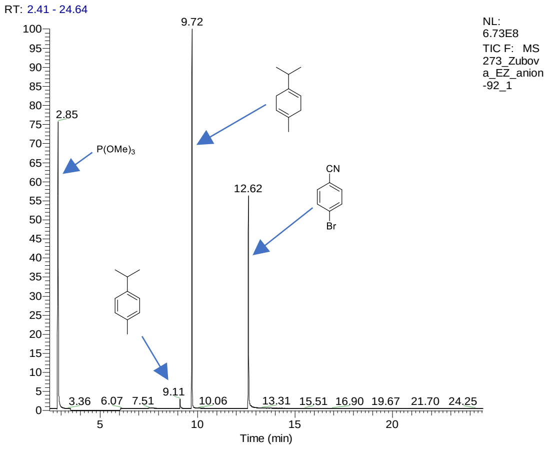


| Apex RT | % Area | | MW Da |
|---|---|---|---|
| 8.34 | 0 | $C_7H_5N$ | 103 |
| 12.62 | 100 | $C_7H_4BrN$ | 181 |
| 17.79 | 0 | $C_9H_{10}NO_3P$ | 211 |

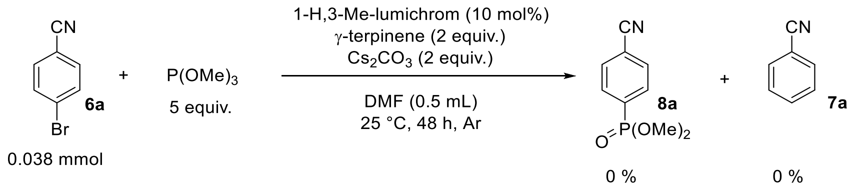


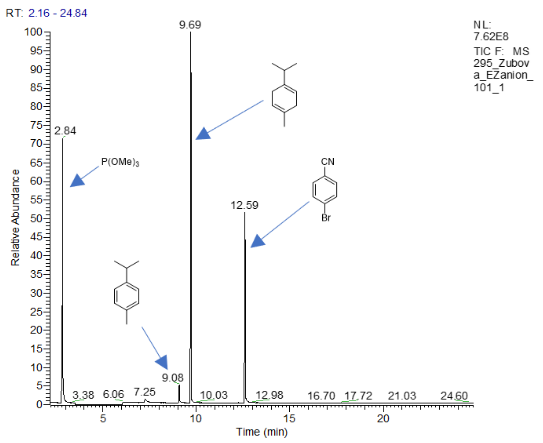


| Apex RT | % Area | | MW Da |
|---|---|---|---|
| 8.34 | 0 | $C_7H_5N$ | 103 |
| 12.59 | 100 | $C_7H_4BrN$ | 181 |
| 17.79 | 0 | $C_9H_{10}NO_3P$ | 211 |

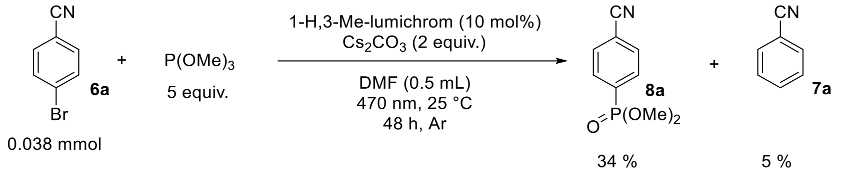


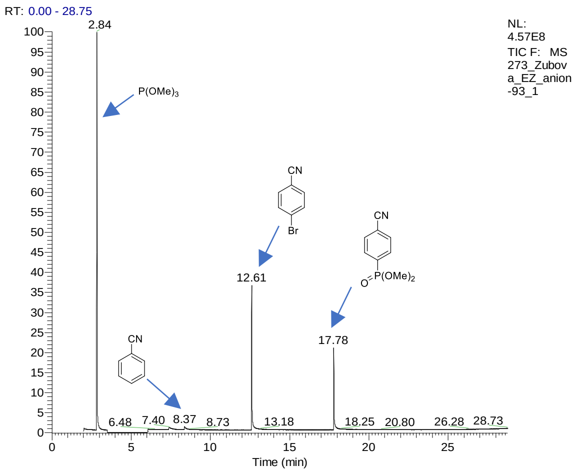


| Apex RT | % Area | | MW Da |
|---|---|---|---|
| 8.37 | 4.58 | $C_7H_5N$ | 103 |
| 12.61 | 61.67 | $C_7H_4BrN$ | 181 |
| 17.78 | 33.75 | $C_9H_{10}NO_3P$ | 211 |

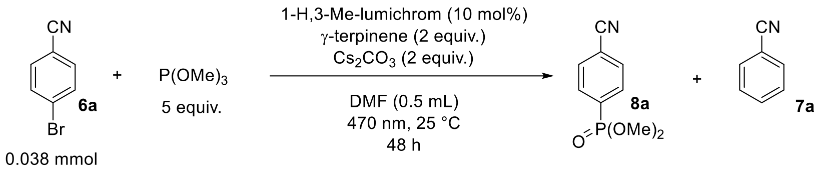


| | | |
|---|---|---|
| **134-2** | 49 % | 4 % |
| **134-3** | 51 % | 4 % |

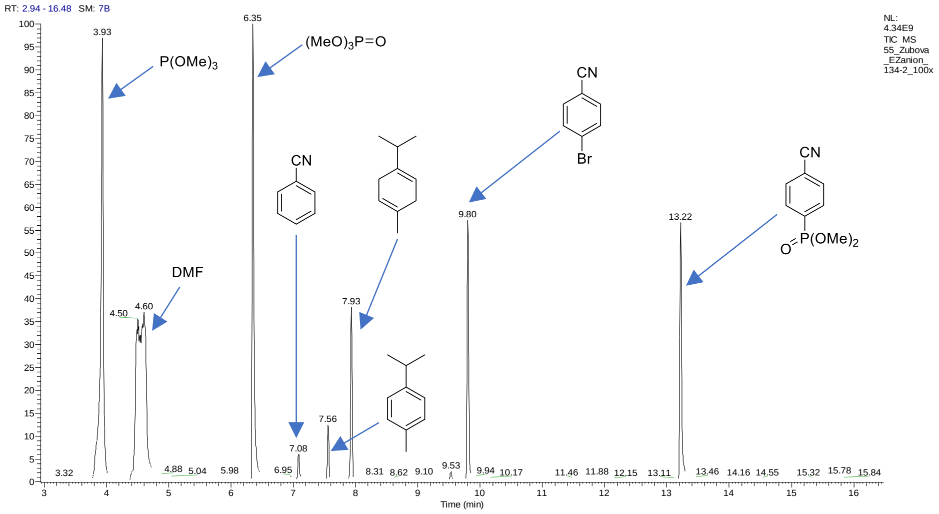


| Apex RT | % Area | | MW Da |
|---|---|---|---|
| 7.08 | 4.33 | $C_7H_5N$ | 103 |
| 9.8 | 46.43 | $C_7H_4BrN$ | 181 |
| 13.22 | 49.24 | $C_9H_{10}NO_3P$ | 211 |

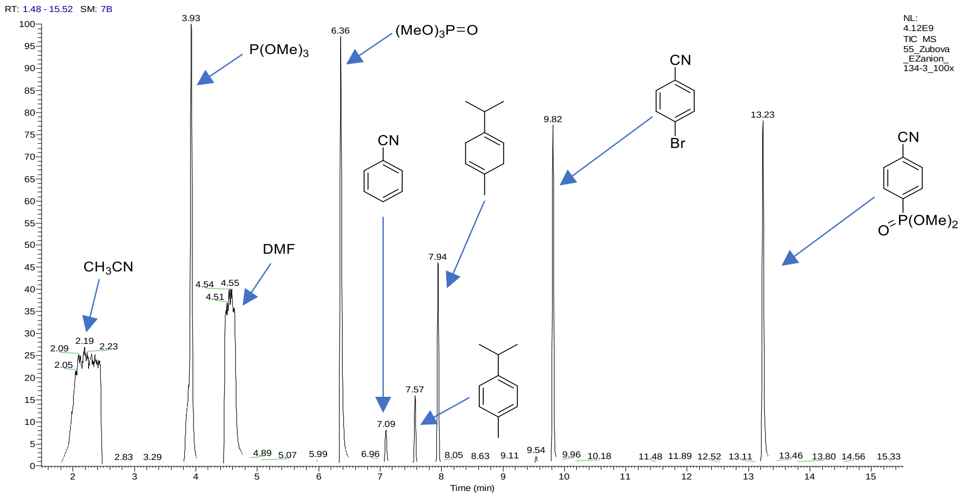


| Apex RT | % Area | | MW Da |
|---|---|---|---|
| 7.09 | 4.16 | $C_7H_5N$ | 103 |
| 9.82 | 44.23 | $C_7H_4BrN$ | 181 |
| 13.23 | 51.61 | $C_9H_{10}NO_3P$ | 211 |

# S9. NMR data for synthesized flavin derivatives

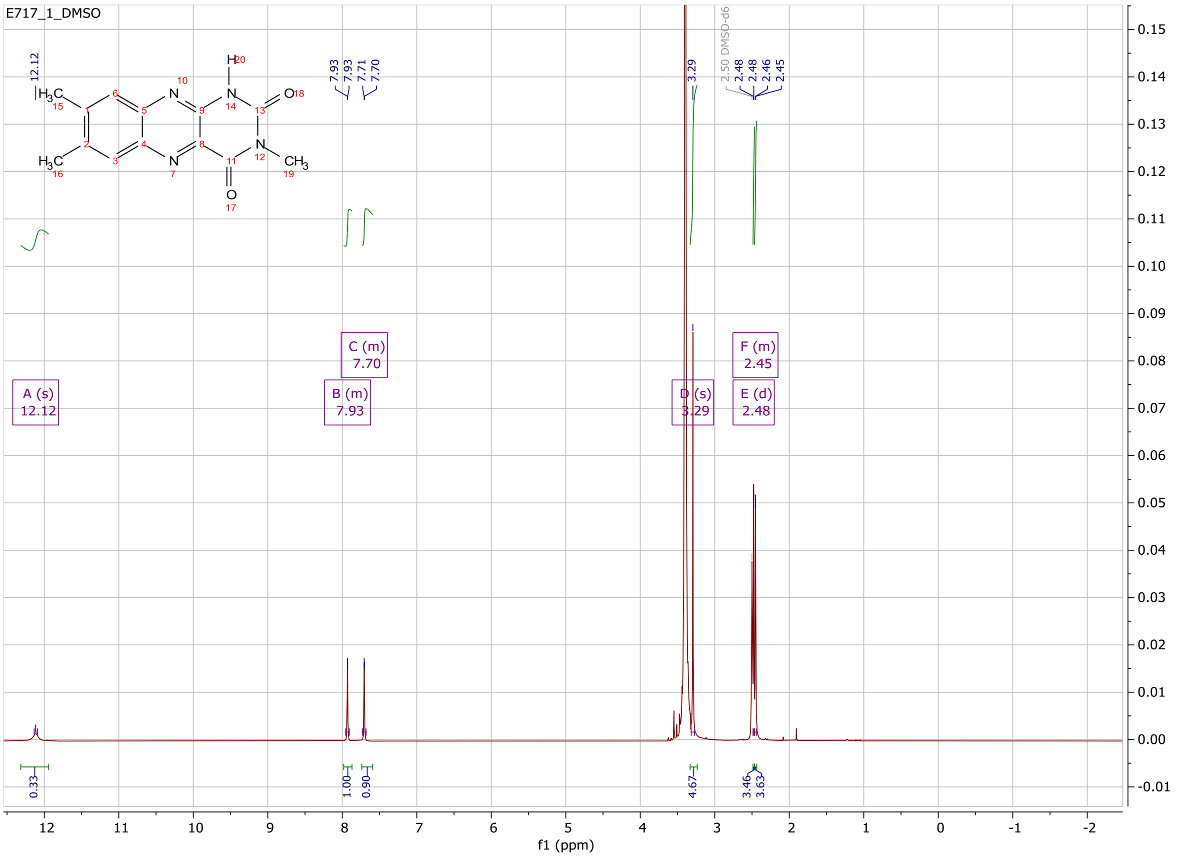


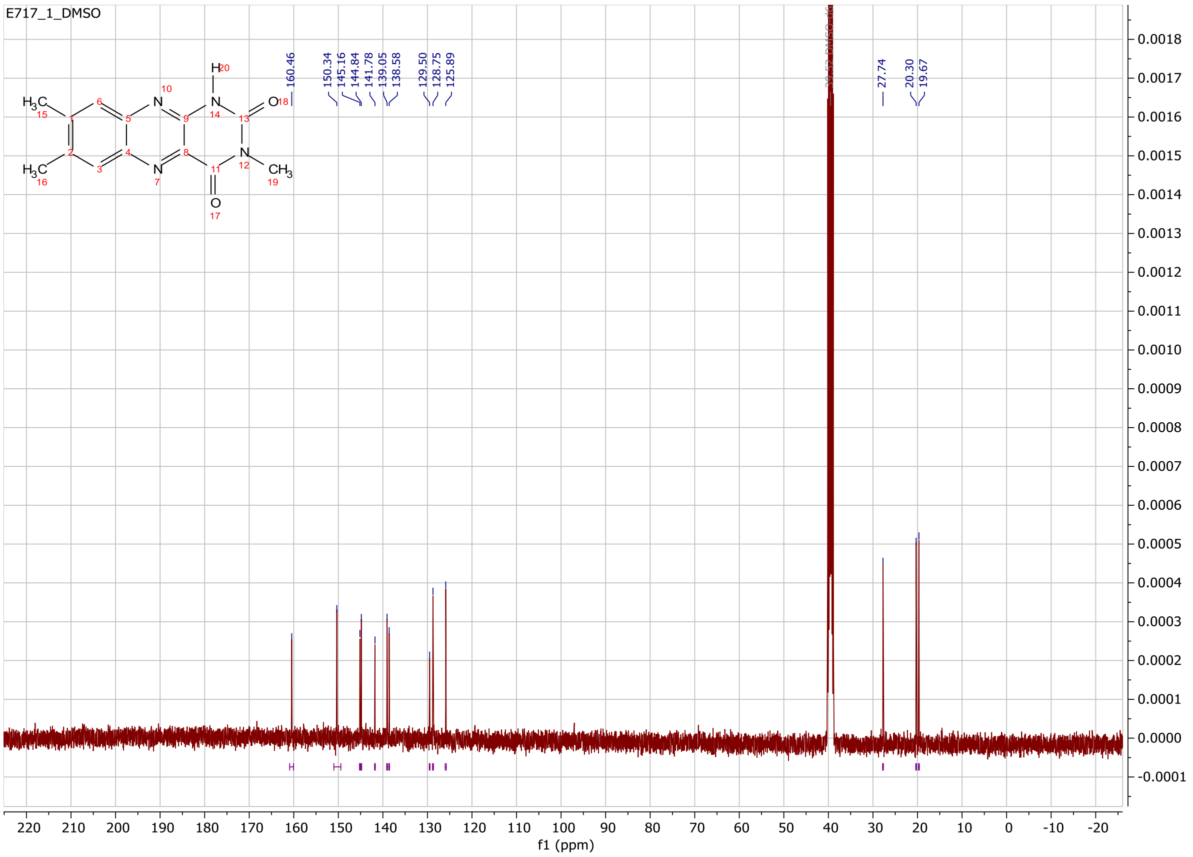


**Figure S9.1** $^{1}H$ and $^{13}C$ NMR spectra of 3-methyllumichrome (3,7,8-trimethylalloxazine (**1a-H**))

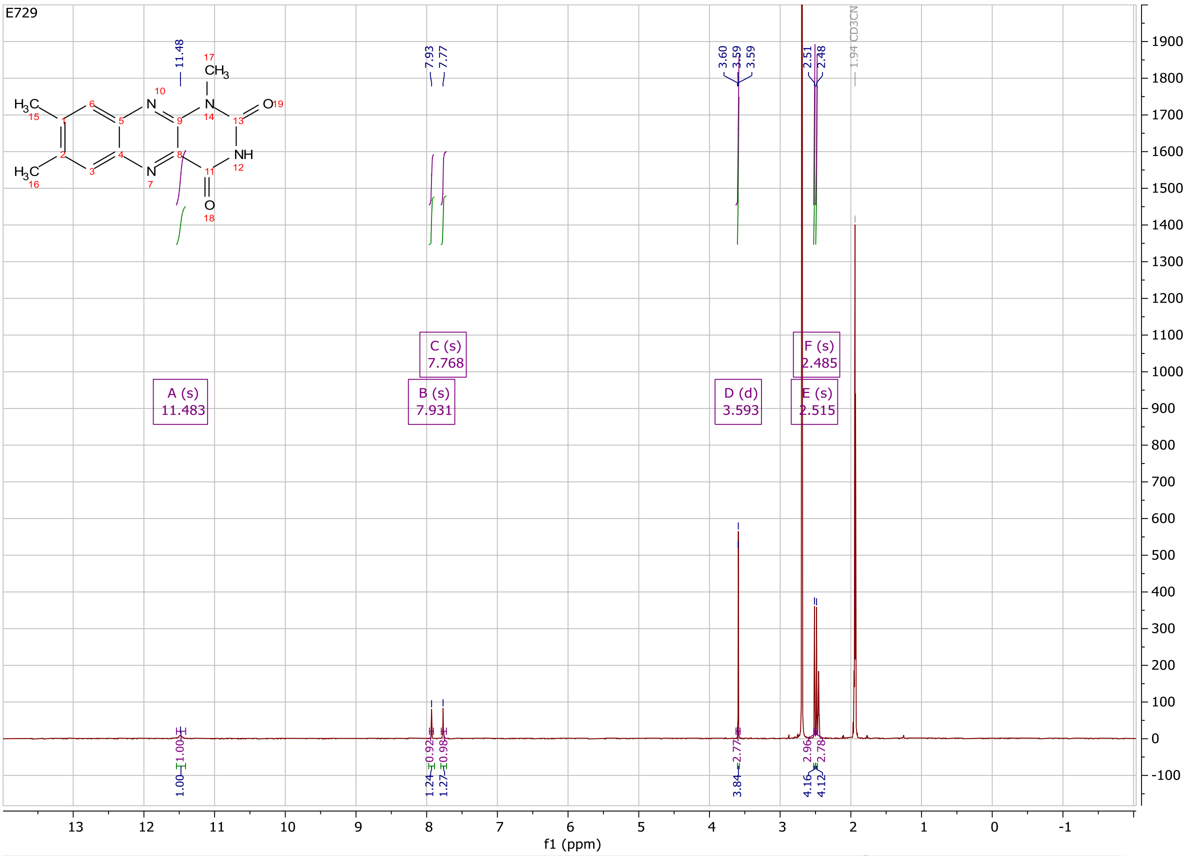


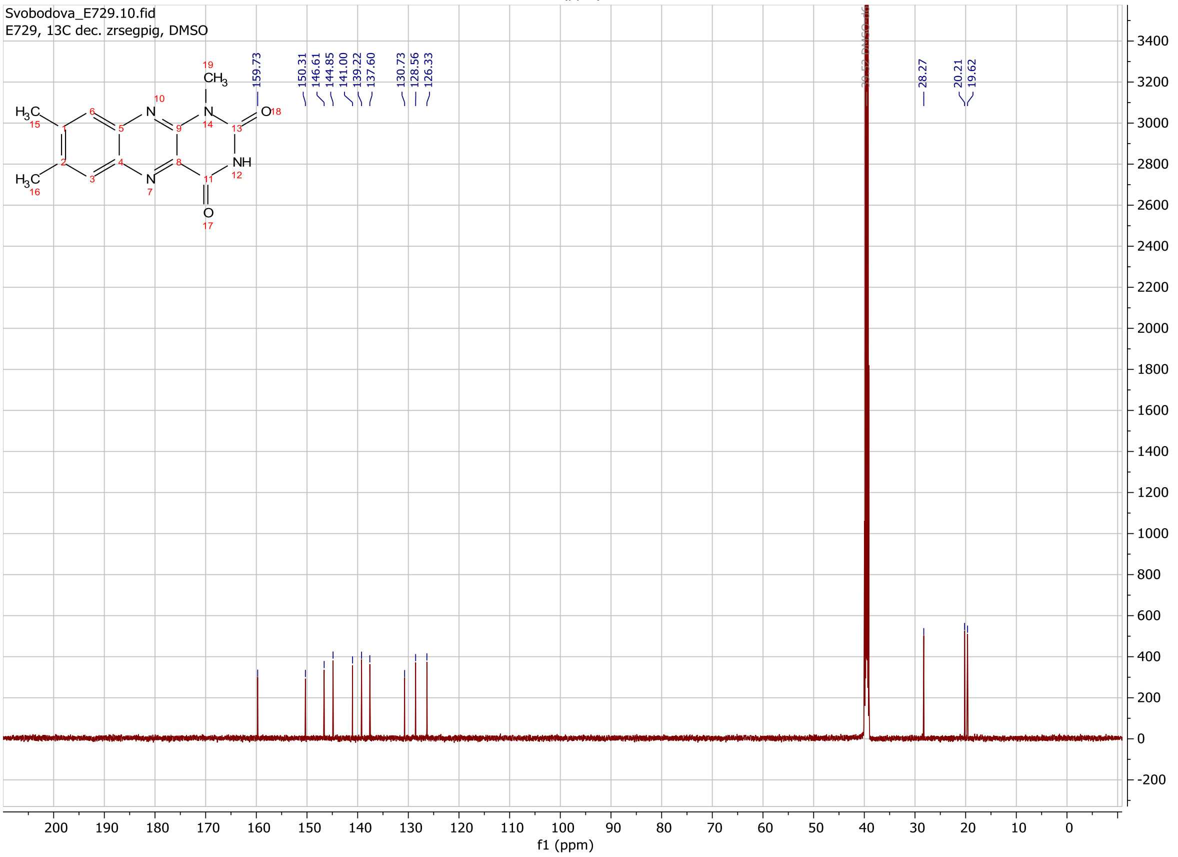


**Figure S9.2** $^{1}H$ and $^{13}C$ NMR spectra of 1-methyllumichrome (1,7,8-trimethylalloxazine (**3a-H**))

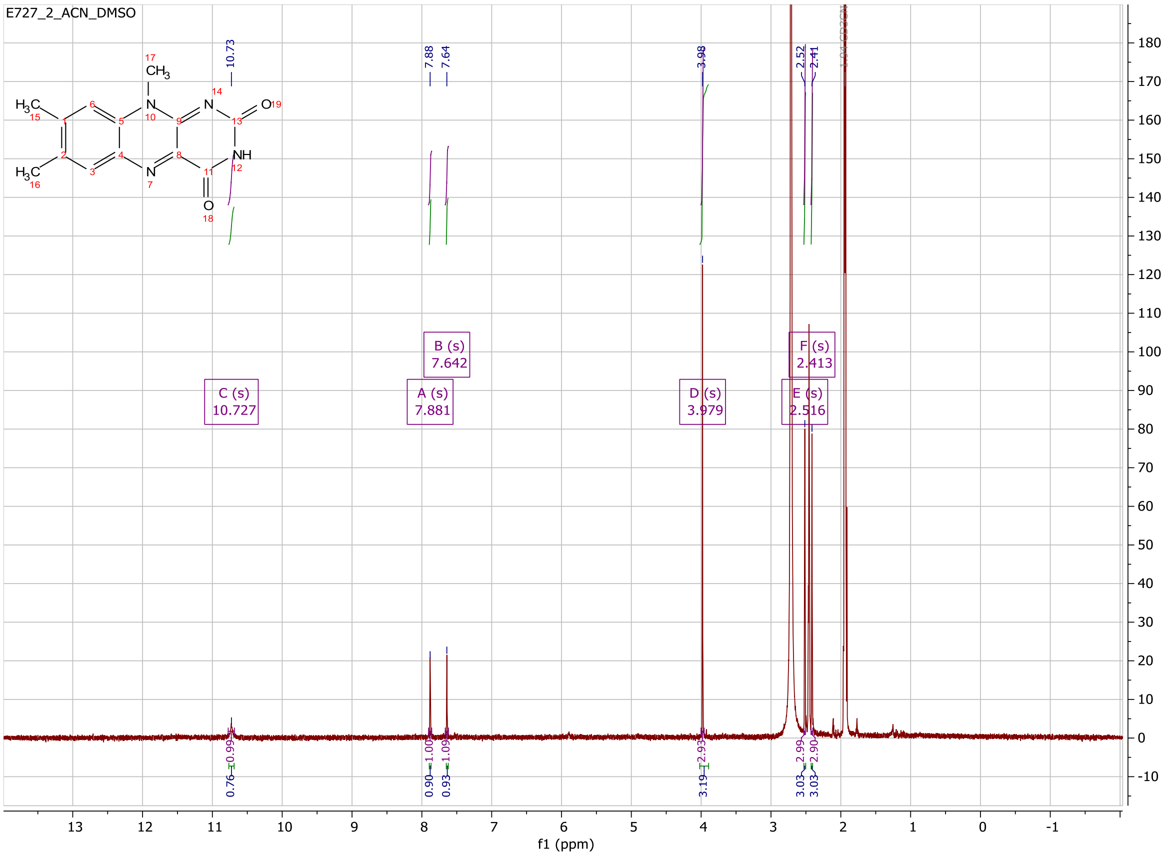


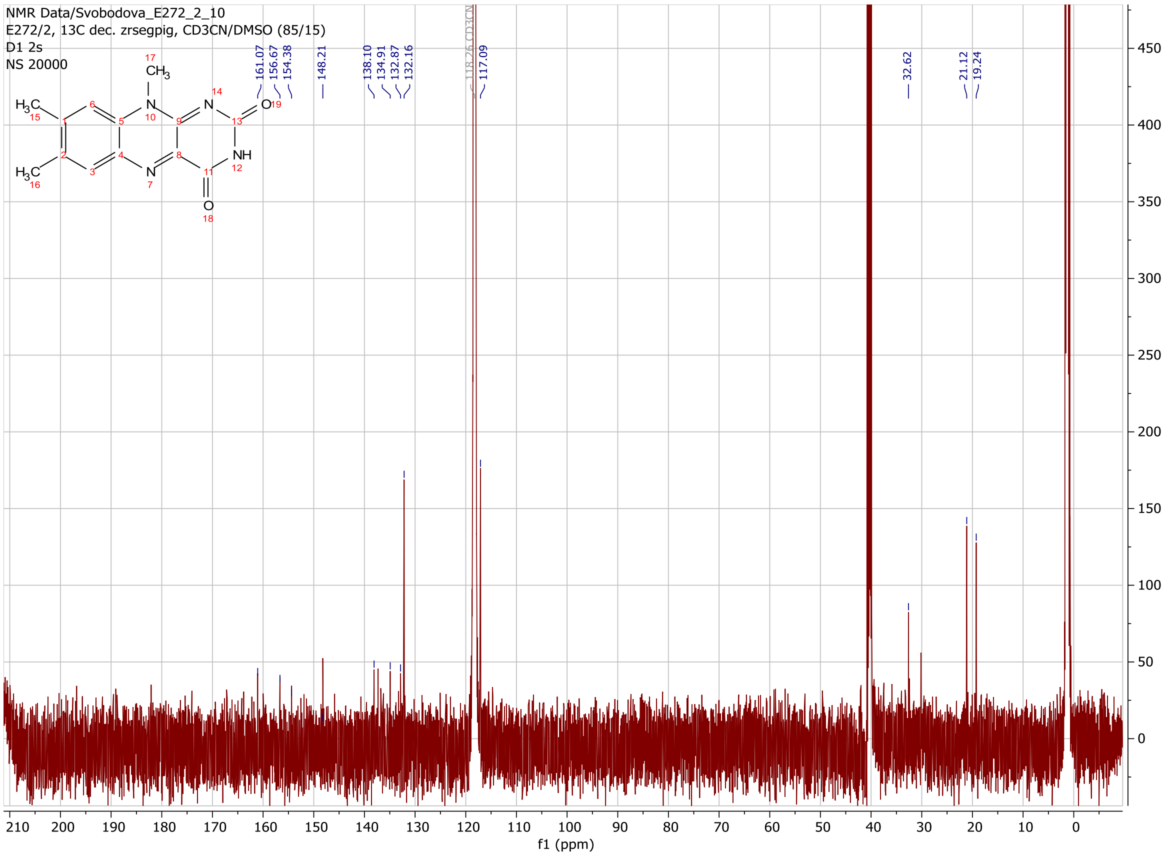


**Figure S9.3** $^{1}$H and $^{13}$C NMR spectra of lumiflavine (7,8,10-trimethylisoalloxazine (**4a-H**))

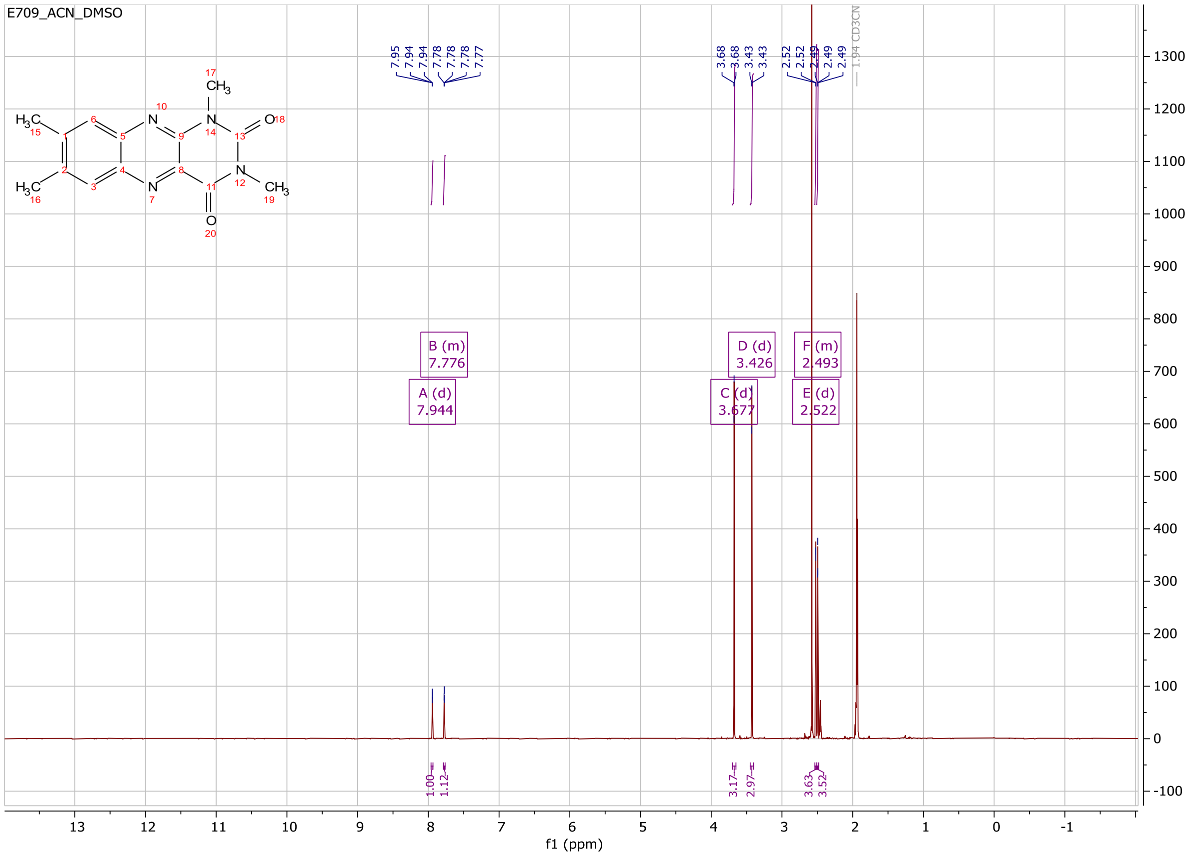


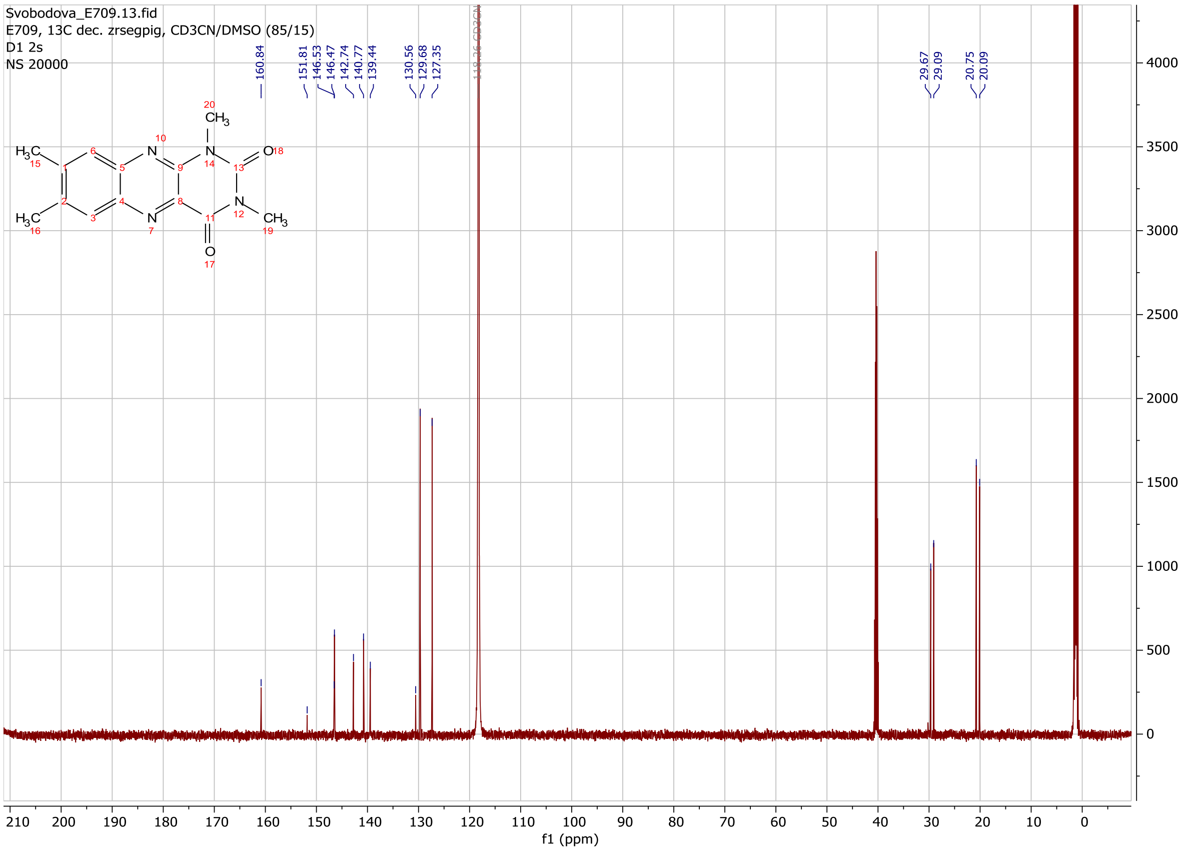


**Figure S9.4** $^{1}H$ and $^{13}C$ NMR spectra of 1.3-dimethyllumichrome (1,3,7,8-tetramethylalloxazine (**5a**))

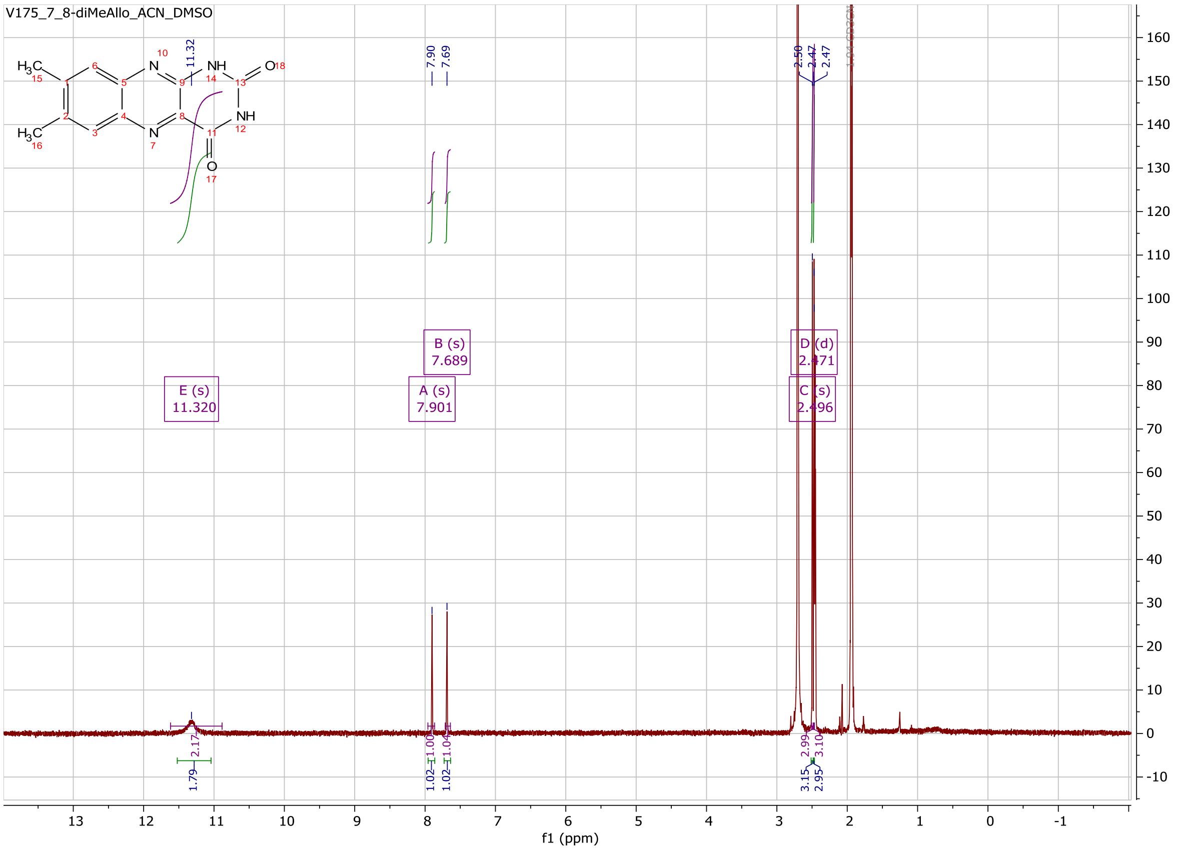


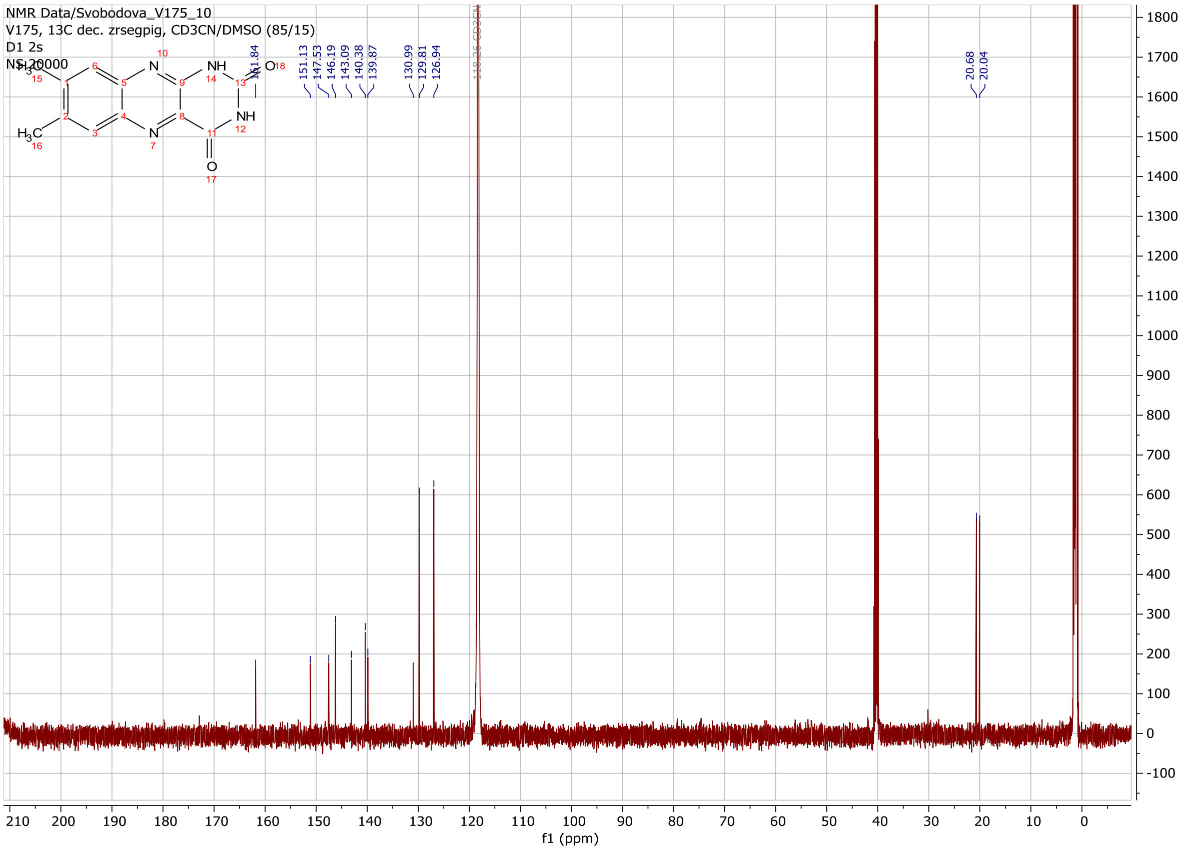


**Figure S9.5** $^{1}H$ and $^{13}C$ NMR spectra of lumichrome (7,8-dimethylalloxazine)

# S10. Natural population analysis data for anions

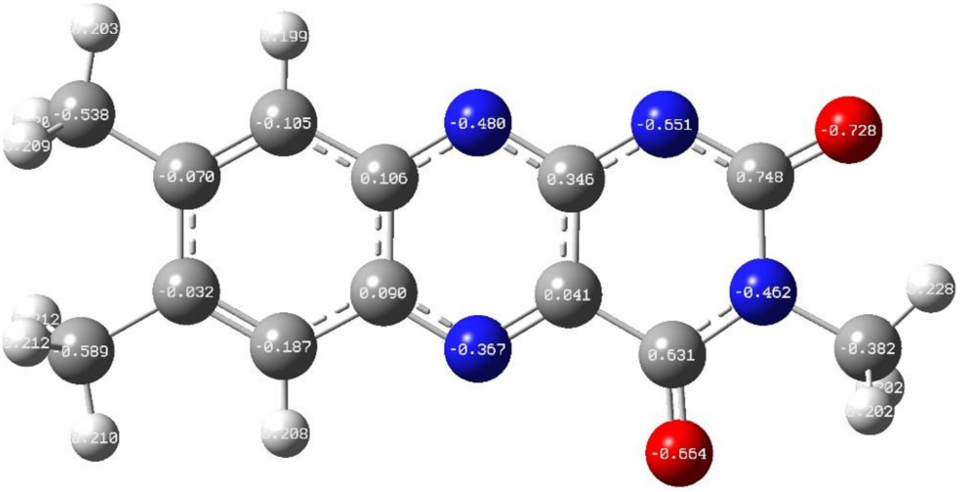


**Figure S10.1** The atomic charge distribution for an anion formed from **1a-H** based on NBO (Natural Bond Orbital) analysis.

**Table S10.1** Summary of natural population analysis for anion formed from **1a-H**

| | | | Natural Population | | | |
|---|---|---|---|---|---|---|
| Atom | No | Natural Charge | Core | Valence | Rydberg | Total |
| C | 1 | -0.18657 | 1.99906 | 4.16753 | 0.01999 | 6.18657 |
| C | 2 | 0.09038 | 1.99905 | 3.88146 | 0.02911 | 5.90962 |
| C | 3 | 0.10623 | 1.99914 | 3.84291 | 0.05172 | 5.89377 |
| C | 4 | -0.10470 | 1.99906 | 4.09301 | 0.01262 | 6.10470 |
| C | 5 | -0.06976 | 1.99921 | 4.01122 | 0.05933 | 6.06976 |
| C | 6 | -0.03169 | 1.99908 | 4.00959 | 0.02303 | 6.03169 |
| H | 7 | 0.20773 | 0.00000 | 0.78753 | 0.00474 | 0.79227 |
| H | 8 | 0.19870 | 0.00000 | 0.79122 | 0.01007 | 0.80130 |
| C | 9 | 0.04137 | 1.99902 | 3.93066 | 0.02895 | 5.95863 |
| C | 10 | 0.34558 | 1.99915 | 3.62040 | 0.03487 | 5.65442 |
| N | 11 | -0.48015 | 1.99941 | 5.44202 | 0.03872 | 7.48015 |
| C | 12 | 0.63069 | 1.99929 | 3.33400 | 0.03602 | 5.36931 |
| C | 13 | 0.74767 | 1.99938 | 3.21073 | 0.04222 | 5.25233 |
| N | 14 | -0.65107 | 1.99936 | 5.61198 | 0.03973 | 7.65107 |
| N | 15 | -0.46154 | 1.99933 | 5.43695 | 0.02526 | 7.46154 |
| O | 16 | -0.66417 | 1.99979 | 6.63891 | 0.02547 | 8.66417 |
| O | 17 | -0.72821 | 1.99979 | 6.70307 | 0.02535 | 8.72821 |
| C | 18 | -0.38239 | 1.99940 | 4.36740 | 0.01558 | 6.38239 |
| H | 19 | 0.22849 | 0.00000 | 0.76879 | 0.00272 | 0.77151 |
| H | 20 | 0.20241 | 0.00000 | 0.79485 | 0.00274 | 0.79759 |
| H | 21 | 0.20243 | 0.00000 | 0.79483 | 0.00274 | 0.79757 |
| C | 22 | -0.53776 | 1.99918 | 4.51858 | 0.02000 | 6.53776 |
| H | 23 | 0.20859 | 0.00000 | 0.78613 | 0.00528 | 0.79141 |
| H | 24 | 0.20289 | 0.00000 | 0.79182 | 0.00528 | 0.79711 |
| H | 25 | 0.20858 | 0.00000 | 0.78613 | 0.00528 | 0.79142 |
| C | 26 | -0.58945 | 1.99918 | 4.57721 | 0.01307 | 6.58945 |
| H | 27 | 0.21176 | 0.00000 | 0.78466 | 0.00358 | 0.78824 |
| H | 28 | 0.21176 | 0.00000 | 0.78466 | 0.00358 | 0.78824 |
| H | 29 | 0.21046 | 0.00000 | 0.78675 | 0.00279 | 0.78954 |
| N | 30 | -0.36706 | 1.99941 | 5.33717 | 0.03047 | 7.36706 |
| * Total * | | -0.99877 | 37.98627 | 95.39218 | 0.62031 | 133.99877 |

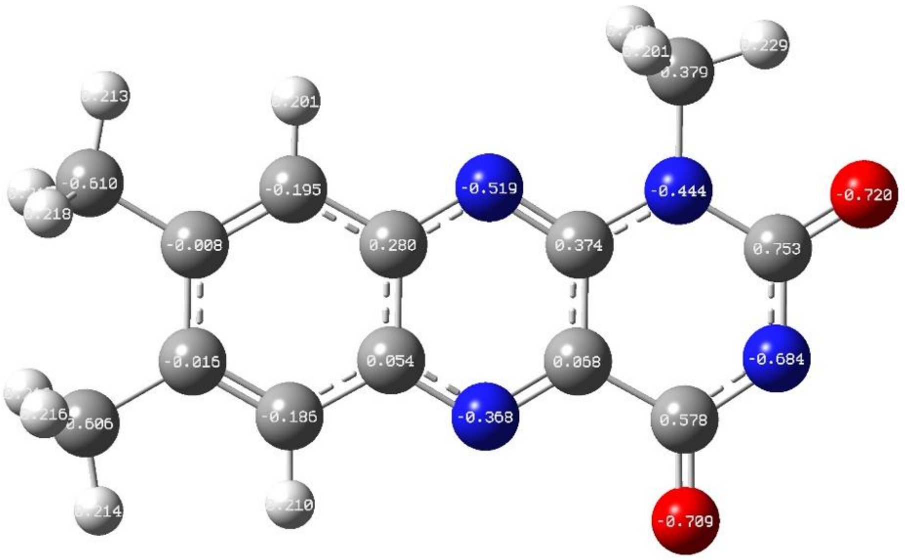


**Figure S10.2** The atomic charge distribution for an anion formed from **3a-H** based on NBO (Natural Bond Orbital) analysis

**Table S10.2** Summary of natural population analysis for anion formed from **3a-H**

| Atom | No | Natural Charge | Natural Population Core | Valence | Rydberg | Total |
|---|---|---|---|---|---|---|
| C | 1 | -0.18613 | 1.99906 | 4.16701 | 0.02006 | 6.18613 |
| C | 2 | 0.05383 | 1.99912 | 3.90064 | 0.04641 | 5.94617 |
| C | 3 | 0.27954 | 1.99909 | 3.67916 | 0.04222 | 5.72046 |
| C | 4 | -0.19517 | 1.99915 | 4.15753 | 0.03848 | 6.19517 |
| C | 5 | -0.00833 | 1.99913 | 3.97821 | 0.03099 | 6.00833 |
| C | 6 | -0.01585 | 1.99907 | 3.99398 | 0.02281 | 6.01585 |
| H | 7 | 0.20988 | 0.00000 | 0.78580 | 0.00433 | 0.79012 |
| H | 8 | 0.20115 | 0.00000 | 0.79177 | 0.00708 | 0.79885 |
| C | 9 | 0.06843 | 1.99901 | 3.90121 | 0.03135 | 5.93157 |
| C | 10 | 0.37363 | 1.99911 | 3.59642 | 0.03084 | 5.62637 |
| N | 11 | -0.51948 | 1.99944 | 5.45812 | 0.06192 | 7.51948 |
| C | 12 | 0.57774 | 1.99932 | 3.37714 | 0.04581 | 5.42226 |
| C | 13 | 0.75321 | 1.99939 | 3.20479 | 0.04261 | 5.24679 |
| N | 14 | -0.44374 | 1.99931 | 5.41657 | 0.02786 | 7.44374 |
| N | 15 | -0.68359 | 1.99936 | 5.64353 | 0.04069 | 7.68359 |
| O | 16 | -0.70892 | 1.99980 | 6.68423 | 0.02489 | 8.70892 |
| O | 17 | -0.72009 | 1.99979 | 6.69495 | 0.02536 | 8.72009 |
| C | 18 | -0.60958 | 1.99942 | 4.59803 | 0.01213 | 6.60958 |
| H | 19 | 0.21805 | 0.00000 | 0.77921 | 0.00275 | 0.78195 |
| H | 20 | 0.21335 | 0.00000 | 0.78387 | 0.00277 | 0.78665 |
| H | 21 | 0.21804 | 0.00000 | 0.77921 | 0.00275 | 0.78196 |
| C | 22 | -0.60573 | 1.99942 | 4.59484 | 0.01147 | 6.60573 |
| H | 23 | 0.21590 | 0.00000 | 0.78162 | 0.00248 | 0.78410 |
| H | 24 | 0.21590 | 0.00000 | 0.78162 | 0.00248 | 0.78410 |
| H | 25 | 0.21373 | 0.00000 | 0.78403 | 0.00224 | 0.78627 |
| N | 26 | -0.36752 | 1.99940 | 5.33741 | 0.03070 | 7.36752 |
| C | 27 | -0.37876 | 1.99940 | 4.36468 | 0.01468 | 6.37876 |
| H | 28 | 0.20116 | 0.00000 | 0.79586 | 0.00298 | 0.79884 |
| H | 29 | 0.20116 | 0.00000 | 0.79586 | 0.00298 | 0.79884 |
| H | 30 | 0.22886 | 0.00000 | 0.76847 | 0.00268 | 0.77114 |
| * Total * | | -0.99934 | 37.98678 | 95.37577 | 0.63679 | 133.99934 |

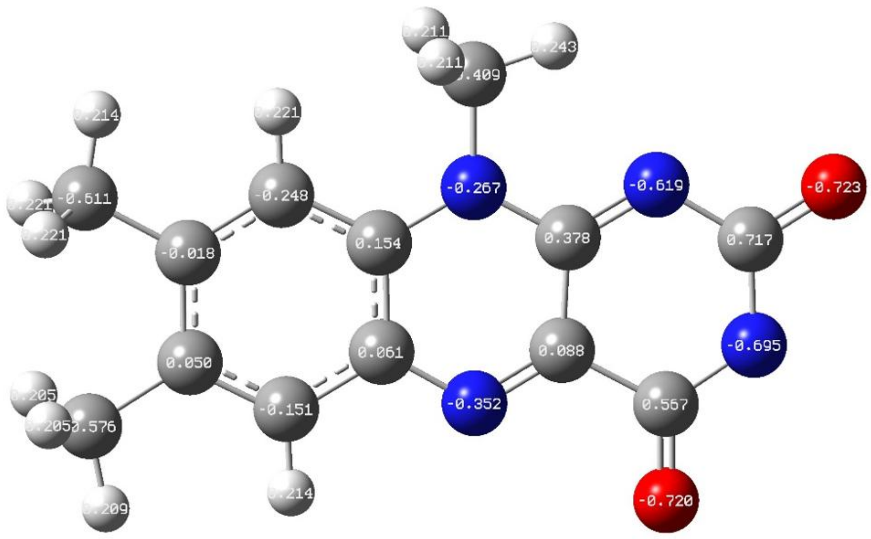


**Figure S10.3** The atomic charge distribution for an anion formed from **4a-H** based on NBO (Natural Bond Orbital) analysis

**Table S10.3** Summary of natural population analysis for anion formed from **4a-H**

| Atom | No | Natural Charge | Natural Population: Core | Valence | Rydberg | Total |
|---|---|---|---|---|---|---|
| C | 1 | -0.15053 | 1.99917 | 4.11343 | 0.03793 | 6.15053 |
| C | 2 | 0.06118 | 1.99905 | 3.90101 | 0.03876 | 5.93882 |
| C | 3 | 0.15448 | 1.99902 | 3.80975 | 0.03675 | 5.84552 |
| C | 4 | -0.24811 | 1.99906 | 4.22993 | 0.01911 | 6.24811 |
| C | 5 | -0.01821 | 1.99918 | 3.96954 | 0.04950 | 6.01821 |
| C | 6 | 0.05002 | 1.99914 | 3.88595 | 0.06489 | 5.94998 |
| H | 7 | 0.21356 | 0.00000 | 0.78161 | 0.00482 | 0.78644 |
| H | 8 | 0.22096 | 0.00000 | 0.77637 | 0.00267 | 0.77904 |
| C | 9 | 0.08756 | 1.99904 | 3.88072 | 0.03269 | 5.91244 |
| C | 10 | 0.37762 | 1.99915 | 3.58291 | 0.04032 | 5.62238 |
| N | 11 | -0.26667 | 1.99931 | 5.23301 | 0.03436 | 7.26667 |
| C | 12 | 0.56657 | 1.99933 | 3.38699 | 0.04712 | 5.43343 |
| C | 13 | 0.71696 | 1.99944 | 3.23875 | 0.04484 | 5.28304 |
| N | 14 | -0.61885 | 1.99933 | 5.58912 | 0.03041 | 7.61885 |
| N | 15 | -0.69452 | 1.99937 | 5.65388 | 0.04127 | 7.69452 |
| O | 16 | -0.72036 | 1.99980 | 6.69567 | 0.02489 | 8.72036 |
| O | 17 | -0.72264 | 1.99979 | 6.69736 | 0.02549 | 8.72264 |
| C | 18 | -0.61073 | 1.99942 | 4.59840 | 0.01292 | 6.61073 |
| H | 19 | 0.22103 | 0.00000 | 0.77655 | 0.00242 | 0.77897 |
| H | 20 | 0.21406 | 0.00000 | 0.78341 | 0.00252 | 0.78594 |
| H | 21 | 0.22104 | 0.00000 | 0.77655 | 0.00242 | 0.77896 |
| C | 22 | -0.57625 | 1.99950 | 4.54983 | 0.02692 | 6.57625 |
| H | 23 | 0.20469 | 0.00000 | 0.78883 | 0.00647 | 0.79531 |
| H | 24 | 0.20469 | 0.00000 | 0.78883 | 0.00647 | 0.79531 |
| H | 25 | 0.20867 | 0.00000 | 0.78664 | 0.00469 | 0.79133 |
| N | 26 | -0.35221 | 1.99940 | 5.32153 | 0.03129 | 7.35221 |
| C | 27 | -0.40888 | 1.99943 | 4.38796 | 0.02149 | 6.40888 |
| H | 28 | 0.21135 | 0.00000 | 0.78618 | 0.00247 | 0.78865 |
| H | 29 | 0.21135 | 0.00000 | 0.78618 | 0.00247 | 0.78865 |
| H | 30 | 0.24310 | 0.00000 | 0.75258 | 0.00432 | 0.75690 |
| * Total * | | -0.99906 | 37.98691 | 95.30947 | 0.70268 | 133.99906 |

## S11. Cartesian coordinates

Cartesian coordinates (in Å) of the ground state singlet equilibrium structure of anion from **1a-H**.

| | | | |
|---|---|---|---|
| C | -2.406395 | 1.359199 | -0.000116 |
| C | -1.204684 | 0.612161 | 0.000027 |
| C | -1.264716 | -0.813869 | 0.000167 |
| C | -2.543762 | -1.423617 | 0.000278 |
| C | -3.701528 | -0.684512 | 0.000106 |
| C | -3.635269 | 0.750106 | -0.000127 |
| H | -2.327054 | 2.438991 | -0.000213 |
| H | -2.585626 | -2.505283 | 0.000508 |
| C | 1.068619 | 0.494604 | 0.000050 |
| C | 1.032212 | -0.944853 | 0.000075 |
| N | -0.150574 | -1.570651 | 0.000187 |
| C | 2.386743 | 1.164149 | 0.000098 |
| C | 3.358700 | -1.120323 | -0.000199 |
| N | 2.150098 | -1.696631 | -0.000033 |
| N | 3.464448 | 0.313704 | 0.000161 |
| O | 2.533211 | 2.385448 | 0.000158 |
| O | 4.416942 | -1.766559 | -0.000646 |
| C | 4.788431 | 0.933833 | 0.000214 |
| H | 5.526391 | 0.142924 | 0.000719 |
| H | 4.908837 | 1.560233 | -0.882473 |
| H | 4.908387 | 1.561003 | 0.882395 |
| C | -5.037483 | -1.375001 | 0.000139 |
| H | -5.627856 | -1.097644 | -0.875902 |
| H | -4.918492 | -2.456553 | 0.000471 |
| H | -5.628254 | -1.097055 | 0.875680 |
| C | -4.896701 | 1.570250 | -0.000386 |
| H | -5.513143 | 1.357533 | -0.876664 |
| H | -5.513496 | 1.357733 | 0.875657 |
| H | -4.667365 | 2.634181 | -0.000506 |
| N | -0.010908 | 1.242157 | 0.000011 |

Cartesian coordinates (in Å) of the ground state singlet equilibrium structure of **1a-H**.

| | | | |
|---|---|---|---|
| C | -2.435661 | -1.368890 | -0.000053 |
| C | -1.231071 | -0.627081 | -0.000011 |
| C | -1.293964 | 0.801253 | 0.000028 |
| C | -2.559312 | 1.422880 | 0.000006 |
| C | -3.719935 | 0.686561 | -0.000038 |
| C | -3.658506 | -0.749538 | -0.000068 |
| H | -2.361799 | -2.448208 | -0.000073 |
| H | -2.593861 | 2.504026 | 0.000036 |
| C | 1.043238 | -0.526480 | 0.000031 |
| C | 0.970684 | 0.890120 | 0.000035 |
| N | -0.164365 | 1.546811 | 0.000051 |
| C | 2.369575 | -1.188418 | 0.000081 |
| C | 3.407094 | 1.061477 | 0.000001 |
| N | 2.146832 | 1.602561 | 0.000000 |
| N | 3.470499 | -0.334739 | -0.000059 |
| O | 2.521137 | -2.396391 | 0.000191 |
| O | 4.400385 | 1.767781 | 0.000004 |

| | | | |
|---|---|---|---|
| C | 4.793913 | -0.968118 | -0.000178 |
| H | 5.542190 | -0.186796 | -0.000844 |
| H | 4.901136 | -1.591043 | 0.884554 |
| H | 4.900476 | -1.591970 | -0.884313 |
| H | 2.096032 | 2.610987 | 0.000082 |
| C | -5.051521 | 1.380791 | 0.000011 |
| H | -5.641165 | 1.102401 | 0.875884 |
| H | -4.929819 | 2.461568 | -0.000119 |
| H | -5.641336 | 1.102181 | -0.875676 |
| C | -4.923785 | -1.560809 | -0.000041 |
| H | -5.536706 | -1.340580 | 0.876303 |
| H | -5.536909 | -1.340350 | -0.876181 |
| H | -4.702620 | -2.625920 | -0.000193 |
| N | -0.043294 | -1.264618 | 0.000030 |

Cartesian coordinates (in Å) of the ground state singlet equilibrium structure of **2a-H**.

| | | | |
|---|---|---|---|
| C | -2.405274 | -1.368265 | 0.000072 |
| C | -1.216673 | -0.612790 | 0.000115 |
| C | -1.312599 | 0.799293 | 0.000059 |
| C | -2.563308 | 1.417056 | -0.000065 |
| C | -3.720318 | 0.658476 | -0.000112 |
| C | -3.642773 | -0.767302 | -0.000017 |
| H | -2.312631 | -2.445690 | 0.000116 |
| H | -2.623252 | 2.497237 | -0.000155 |
| C | 1.076642 | -0.517277 | 0.000190 |
| C | 1.081531 | 0.924912 | 0.000145 |
| N | -0.141265 | 1.509080 | 0.000099 |
| C | 2.401796 | -1.185743 | 0.000170 |
| C | 3.383049 | 1.092656 | 0.000095 |
| N | 2.149995 | 1.679991 | 0.000193 |
| N | 3.480893 | -0.328451 | -0.000268 |
| O | 2.540315 | -2.399210 | 0.000428 |
| O | 4.410996 | 1.757699 | 0.000313 |
| C | 4.807842 | -0.950531 | -0.000872 |
| H | 5.548345 | -0.162314 | -0.003769 |
| H | 4.924111 | -1.572977 | 0.884040 |
| H | 4.921190 | -1.576950 | -0.883297 |
| C | -5.055903 | 1.342087 | -0.000312 |
| H | -5.642013 | 1.055836 | 0.875317 |
| H | -4.946381 | 2.423923 | -0.000380 |
| H | -5.641770 | 1.055677 | -0.876055 |
| C | -4.895055 | -1.599152 | -0.000011 |
| H | -5.511184 | -1.390208 | 0.876711 |
| H | -5.511382 | -1.389941 | -0.876525 |
| H | -4.655817 | -2.660292 | -0.000176 |
| N | -0.007872 | -1.235500 | 0.000181 |
| H | -0.165688 | 2.521432 | 0.000074 |

Cartesian coordinates (in Å) of the ground state singlet equilibrium structure of anion from **3a-H**.

| | | | |
|---|---|---|---|
| C | -2.393936 | -1.364772 | 0.000058 |
| C | -1.118360 | -0.757369 | -0.000050 |
| C | -1.024555 | 0.660755 | -0.000197 |
| C | -2.217611 | 1.415235 | -0.000165 |
| C | -3.453716 | 0.811129 | -0.000040 |
| C | -3.547311 | -0.619010 | 0.000065 |
| H | -2.437250 | -2.446387 | 0.000163 |
| H | -2.135726 | 2.494453 | -0.000216 |
| C | 1.163505 | -0.916327 | -0.000078 |
| C | 1.252000 | 0.514008 | -0.000114 |
| N | 0.180463 | 1.281488 | -0.000235 |
| C | 2.433219 | -1.711304 | -0.000070 |
| C | 3.673464 | 0.316231 | 0.000144 |
| N | 2.492200 | 1.100441 | -0.000026 |
| N | 3.595062 | -1.034007 | 0.000060 |
| O | 2.388693 | -2.953290 | -0.000196 |
| O | 4.761423 | 0.908508 | 0.000364 |
| C | -4.702737 | 1.648341 | 0.000049 |
| H | -5.320590 | 1.439306 | 0.876000 |
| H | -4.462471 | 2.709507 | -0.000107 |
| H | -5.320857 | 1.439087 | -0.875659 |
| C | -4.892162 | -1.293370 | 0.000249 |
| H | -5.480390 | -1.012198 | 0.876479 |
| H | -5.480536 | -1.012353 | -0.875937 |
| H | -4.783340 | -2.376152 | 0.000336 |
| N | 0.001530 | -1.520475 | -0.000025 |
| C | 2.581433 | 2.558369 | 0.000014 |
| H | 2.092853 | 2.968447 | -0.882762 |
| H | 2.092597 | 2.968459 | 0.882628 |
| H | 3.630617 | 2.822452 | 0.000135 |

Cartesian coordinates (in Å) of the ground state singlet equilibrium structure of **3a-H**.

| | | | |
|---|---|---|---|
| C | -2.426442 | -1.375742 | 0.000019 |
| C | -1.149692 | -0.767631 | 0.000008 |
| C | -1.054310 | 0.655895 | 0.000012 |
| C | -2.243519 | 1.413076 | -0.000003 |
| C | -3.478027 | 0.807916 | -0.000004 |
| C | -3.574381 | -0.626114 | 0.000022 |
| H | -2.470527 | -2.456708 | 0.000022 |
| H | -2.159706 | 2.491515 | -0.000019 |
| C | 1.124996 | -0.912201 | -0.000005 |
| C | 1.219401 | 0.511923 | 0.000011 |
| N | 0.149909 | 1.272874 | 0.000017 |
| C | 2.360674 | -1.726148 | -0.000019 |
| C | 3.640213 | 0.417439 | 0.000002 |
| N | 2.462248 | 1.133423 | 0.000016 |
| N | 3.511175 | -0.965172 | -0.000011 |
| O | 2.405263 | -2.942377 | -0.000037 |
| O | 4.746331 | 0.930526 | 0.000000 |
| C | -4.725827 | 1.643457 | -0.000069 |
| H | -5.342494 | 1.430902 | 0.875610 |
| H | -4.487242 | 2.704558 | -0.000042 |
| H | -5.342360 | 1.430927 | -0.875855 |

| | | | |
|---|---|---|---|
| C | -4.920780 | -1.294023 | 0.000043 |
| H | -5.505952 | -1.007956 | 0.876369 |
| H | -5.506051 | -1.007858 | -0.876182 |
| H | -4.817201 | -2.376907 | -0.000017 |
| N | -0.035437 | -1.524923 | -0.000004 |
| C | 2.525556 | 2.598359 | 0.000022 |
| H | 2.029389 | 2.989650 | -0.884593 |
| H | 2.029420 | 2.989647 | 0.884658 |
| H | 3.569320 | 2.884016 | 0.000006 |
| H | 4.388220 | -1.467613 | -0.000018 |

Cartesian coordinates (in Å) of the ground state singlet equilibrium structure of anion from **4a-H**.

| | | | |
|---|---|---|---|
| C | -2.128338 | -1.491367 | 0.000089 |
| C | -0.912857 | -0.787373 | -0.000035 |
| C | -0.935762 | 0.624298 | -0.000102 |
| C | -2.173921 | 1.278947 | -0.000092 |
| C | -3.365178 | 0.569203 | -0.000022 |
| C | -3.348744 | -0.849905 | 0.000119 |
| H | -2.073624 | -2.571972 | 0.000127 |
| H | -2.215843 | 2.356720 | -0.000131 |
| C | 1.380588 | -0.832464 | -0.000051 |
| C | 1.463329 | 0.620322 | -0.000014 |
| N | 0.267643 | 1.304482 | -0.000186 |
| C | 2.694731 | -1.568328 | -0.000163 |
| C | 3.780797 | 0.528750 | 0.000231 |
| N | 2.590335 | 1.268919 | 0.000197 |
| N | 3.818558 | -0.839495 | -0.000019 |
| O | 2.690972 | -2.813726 | -0.000402 |
| O | 4.843824 | 1.176905 | 0.000509 |
| C | -4.670693 | 1.313813 | -0.000035 |
| H | -5.270815 | 1.057507 | 0.875650 |
| H | -4.511936 | 2.390033 | 0.000089 |
| H | -5.270635 | 1.057678 | -0.875892 |
| C | -4.630016 | -1.638549 | 0.000335 |
| H | -5.240021 | -1.410679 | 0.877062 |
| H | -5.240541 | -1.410471 | -0.875950 |
| H | -4.427358 | -2.707757 | 0.000183 |
| N | 0.263034 | -1.486431 | -0.000081 |
| C | 0.272822 | 2.769019 | -0.000316 |
| H | -0.234746 | 3.142395 | -0.888187 |
| H | -0.233476 | 3.142602 | 0.888217 |
| H | 1.303075 | 3.097989 | -0.001066 |

Cartesian coordinates (in Å) of the ground state singlet equilibrium structure of **4a-H**.

| | | | |
|---|---|---|---|
| C | -2.164315 | 1.500543 | -0.000033 |
| C | -0.945907 | 0.794363 | 0.000012 |
| C | -0.965069 | -0.622117 | 0.000034 |
| C | -2.200227 | -1.279175 | 0.000041 |
| C | -3.389614 | -0.569068 | -0.000002 |
| C | -3.377886 | 0.855475 | -0.000061 |
| H | -2.109267 | 2.580543 | -0.000064 |
| H | -2.242700 | -2.356271 | 0.000074 |
| C | 1.337257 | 0.823435 | 0.000046 |

| | | | |
|---|---|---|---|
| C | 1.427866 | -0.622279 | 0.000000 |
| N | 0.238841 | -1.300104 | 0.000050 |
| C | 2.616198 | 1.584694 | 0.000080 |
| C | 3.741024 | -0.628160 | -0.000098 |
| N | 2.556110 | -1.297222 | -0.000063 |
| N | 3.722369 | 0.777736 | -0.000022 |
| O | 2.693669 | 2.801766 | 0.000174 |
| O | 4.830594 | -1.190485 | -0.000194 |
| C | -4.692956 | -1.312694 | 0.000042 |
| H | -5.291363 | -1.052707 | -0.875529 |
| H | -4.535778 | -2.388640 | 0.000037 |
| H | -5.291284 | -1.052689 | 0.875661 |
| C | -4.662366 | 1.635963 | -0.000155 |
| H | -5.269547 | 1.401956 | -0.876782 |
| H | -5.269580 | 1.402103 | 0.876492 |
| H | -4.466021 | 2.705922 | -0.000240 |
| N | 0.220382 | 1.485333 | 0.000037 |
| C | 0.238136 | -2.769493 | 0.000116 |
| H | -0.272489 | -3.132676 | 0.889258 |
| H | -0.272159 | -3.132768 | -0.889181 |
| H | 1.264455 | -3.107327 | 0.000330 |
| H | 4.624870 | 1.233202 | -0.000039 |

## S12. References


1. A. Golczak, D. Prukała, E. Sikorska, M. Gierszewski, V. Cherkas, D. Kwiatek, A. Kubiak, N. Varma, T. Pędziński, S. Murphree, R. Cibulka, L. Mrówczyńska, J. L. Kolanowski, M. Sikorski *Sci. Rep.* **2023**, *13,* 13426.
2. Gaussian 16, Revision C.01, M. J. Frisch, G. W. Trucks, H. B. Schlegel, G. E. Scuseria, M. A. Robb, J. R. Cheeseman, G. Scalmani, V. Barone, G. A. Petersson, H. Nakatsuji, X. Li, M. Caricato, A. V. Marenich, J. Bloino, B. G. Janesko, R. Gomperts, B. Mennucci, H. P. Hratchian, J. V. Ortiz, A. F. Izmaylov, J. L. Sonnenberg, D. Williams-Young, F. Ding, F. Lipparini, F. Egidi, J. Goings, B. Peng, A. Petrone, T. Henderson, D. Ranasinghe, V. G. Zakrzewski, J. Gao, N. Rega, G. Zheng, W. Liang, M. Hada, M. Ehara, K. Toyota, R. Fukuda, J. Hasegawa, M. Ishida, T. Nakajima, Y. Honda, O. Kitao, H. Nakai, T. Vreven, K. Throssell, J. A. Montgomery, Jr., J. E. Peralta, F. Ogliaro, M. J. Bearpark, J. J. Heyd, E. N. Brothers, K. N. Kudin, V. N. Staroverov, T. A. Keith, R. Kobayashi, J. Normand, K. Raghavachari, A. P. Rendell, J. C. Burant, S. S. Iyengar, J. Tomasi, M. Cossi, J. M. Millam, M. Klene, C. Adamo, R. Cammi, J. W. Ochterski, R. L. Martin, K. Morokuma, O. Farkas, J. B. Foresman, and D. J. Fox, Gaussian, Inc., Wallingford CT, 2016.
3. P. Chattopadhyay, R. Nagpal and P. S. Pandey, *Aust. J. Chem.*, 2008, **61**, 216-222.
4. R. Kuhn, K. Reinemund and F. Weygand, *Ber. Deutsch. Chem. Ges.*, 1934, **67**, 1460-1462.
5. F. Müller and K. H. Dudley, *Helv. Chim. Acta*, 1971, **54**, 1487-1497.
6. I. Jhulki, P. K. Chanani, S. H. Abdelwahed and T. P. Begley, *Journal of the American Chemical Society*, 2016, **138**, 8324-8327.
7. S. Chen and F. W. Foss, *Org. Lett.*, 2012, **14**, 5150-5153.
8. A. Yoshimura, M. Z. Hoffman, H. Sun, *J. Photochem. Photobiol. A: Chem.* **1993**, *70*, 29-33.
9. A. Sykes, T. G. Truscott, *Trans. Faraday Soc.*, **1971**, *67*, 679-686.
